# Bortezomib in combination with standard-of-care for late active antibody-mediated rejection with de novo donor-specific antibodies after kidney transplantation: a multicenter randomized trial

**DOI:** 10.3389/ti.2026.16702

**Published:** 2026-09-23

**Authors:** Renaud Snanoudj, Fabienne Marquant, Karine Renaudin, Valérie Dubois, Alice Koenig, Nadia Arzouk, Sophie Caillard, Magali Giral, Arwa Jalal Eddine, Hakim Mazouz, Nassim Kamar, Vincent Pernin, Marie Matignon, Nacera Ouali, Dany Anglicheau, Caroline Elie

**Affiliations:** 1 Nephrology Dialysis and Transplantation Department, Hopital Bicetre, Le Kremlin-Bicêtre, France; 2 Unit of Clinical Research, Hopital Universitaire Necker-Enfants Malades, Paris, France; 3 Pathology Department, Centre Hospitalier Universitaire de Nantes, Nantes, France; 4 Histocompatibility Department, Etablissement Francais du Sang Auvergne-Rhone-Alpes, Décines-Charpieu, France; 5 Transplantation Department, Hospices Civils de Lyon, Lyon, France; 6 Transplantation Department, Hopital Universitaire Pitie-Salpetriere, Paris, France; 7 Nephrology Dialysis and Transplantation Department, Hopital Civil, Strasbourg, France; 8 Nephrology Dialysis and Transplantation Department, Hotel Dieu, Nantes, France; 9 Nephrology Department, Hopital Foch, Suresnes, France; 10 Nephrology and Transplantation Department, Centre Hospitalier Universitaire Amiens-Picardie, Amiens, France; 11 Nephrology and Organ Transplantation Department, Hopital de Rangueil, Toulouse, France; 12 Nephrology and Organ Transplantation Department, Hopital Lapeyronie, Montpellier, France; 13 Nephrology and Organ Transplantation Department, Hopitaux Universitaires Henri Mondor, Créteil, France; 14 Nephrology and Organ Transplantation Department, Hopital Tenon, Paris, France; 15 Department of Kidney and Metabolic Diseases, Transplantation and Clinical Immunology, AP-HP, Necker Hospital, Université Paris Cité, Paris, France; 16 Pharmacologie et Évaluations des Thérapeutiques Chez l’Enfant et la Femme Enceinte, Inserm, Universite Paris Cite, Paris, France

**Keywords:** antibody-mediated rejection, bortezomib, donor-specific antibodies, immunosuppression, kidney transplantation

## Abstract

Bortezomib has been proposed as a treatment for antibody-mediated rejection (AMR) after kidney transplantation; however, its efficacy remains uncertain. We conducted a multicenter, randomized, open-label trial in France between February 2015 and July 2019 in adult kidney transplant recipients with late active AMR associated with *de novo* donor-specific antibodies (dnDSA). Sixty of the planned 100 patients were randomized 1:1 to receive standard-of-care consisting of corticosteroids, plasmapheresis, and intravenous immunoglobulin, either alone or with bortezomib administered as two cycles of four infusions. The primary endpoint was a composite of a >50% reduction in immunodominant DSA mean fluorescence intensity and stabilization or improvement of microvascular inflammation and transplant glomerulopathy on graft biopsy at 12 months. This endpoint was achieved in 40.0% of patients receiving bortezomib and 33.3% receiving standard-of-care alone in the intention-to-treat analysis (RR = 1.20, 95% CI 0.61–2.34; p = 0.59), and in 50.0% and 42.1%, respectively, in the per-protocol analysis (RR = 1.19, 95% CI 0.60–2.36; p = 0.62). Neither component differed between groups. Serious adverse events occurred in 23 patients (76.7%) receiving bortezomib and 14 patients (46.7%) receiving standard-of-care alone (p = 0.017). Adding bortezomib did not demonstrate a significant efficacy benefit and was associated with a higher incidence of serious adverse events (ClinicalTrials.gov number: NCT02201576).

## Graphical Abstract

**Figure:**
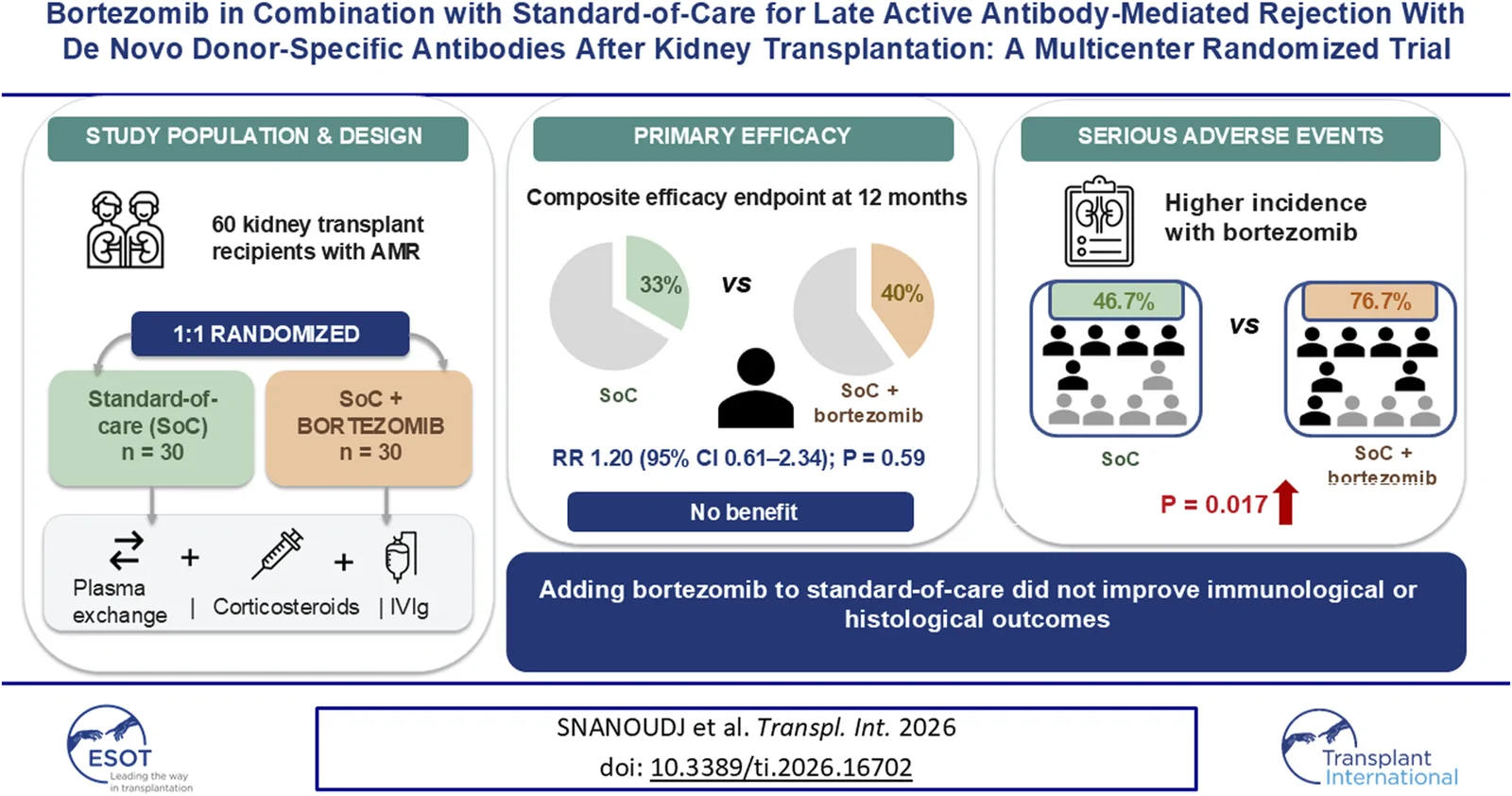

## Introduction

Within ten years of kidney transplantation, up to 25% of recipients develop *de novo* donor-specific antibodies (dnDSA), most of which are class II anti-human leukocyte antigen (HLA) antibodies [1]. The occurrence of dnDSA is associated with a 40% reduction in 10-year graft survival [2] and can trigger active or chronic active antibody-mediated rejection (AMR). These late AMR episodes frequently occur subclinically several years post-transplantation, unlike AMR associated with pre-existing donor-specific antibodies [3]. They are now considered one of the leading causes of long-term graft failure [4, 5].

To date, no approved treatments exist for AMR. The most widely accepted standard-of-care consists of corticosteroids, plasmapheresis, and intravenous immunoglobulin (IVIg), although this approach is supported by low-quality evidence. Several additional therapies are frequently used by transplant centers as adjuncts to standard-of-care including anti-CD20 monoclonal antibodies (mAb), complement inhibitors, anti-IL-6 antibodies, and plasma cell-targeting agents, for which evidence also remains low and anecdotal.

Among plasma cell-targeting therapies, bortezomib, a proteasome inhibitor indicated for multiple myeloma, has been proposed to treat AMR because of its ability to induce apoptosis of plasma cells, which produce anti-HLA antibodies. Uncontrolled studies have shown contrasting results with bortezomib in the context of pre-transplant desensitization [6–9].

Small-scale, single-center retrospective studies conducted in patients with AMR have reported reductions in immunodominant DSA (idDSA) following bortezomib treatment [10, 11], whereas others have found no such effect [12, 13]. Thus, no definitive conclusions can be drawn regarding its effect on AMR outcomes. More recently, the randomized BORTEJECT study demonstrated no benefit of bortezomib monotherapy over placebo in slowing the decline of glomerular filtration rate (GFR) in patients screened for DSA and displaying biopsy-proven AMR [14]. However, in that study, bortezomib was used as monotherapy.

To better assess the role of bortezomib, our objective was to perform a randomized controlled trial in a homogeneous cohort of kidney transplant recipients with active AMR and dnDSA. Specifically, we evaluated the efficacy of adding bortezomib to standard-of-care, including plasmapheresis, corticosteroids, and IVIg. We hypothesized that proteasome inhibitor therapy could be beneficial when combined with interventions targeting circulating alloantibodies (plasmapheresis and IVIg), while corticosteroids may enhance the pro-apoptotic effects of proteasome inhibitor therapy on plasma cells.

## Materials and methods

### Study design

The last version of the protocol is available in Supplementary Material. A randomized controlled, non-blinded multicenter superiority trial was conducted at 15 centers in France between February 12, 2015, and July 25, 2019. Among the centers, 11 sites enrolled patients including Paris (three sites), Lyon, Nantes, Toulouse, Montpellier, Suresnes, Créteil, Strasbourg, and Amiens. Eligible patients were randomized in a 1:1 ratio to receive bortezomib or to the standard-of-care group using a centralized web-based randomization system (CleanWeb^TM^; Telemedicine Technologies SAS, France). Randomization occurred after AMR was confirmed by an inpatient hospital transplant biopsy, eligibility criteria were checked, and informed consent was obtained. Randomization was stratified according to the center and type of rejection, clinical or subclinical. AMR was classified as clinical if the biopsy was performed for graft dysfunction or new-onset proteinuria, and subclinical if performed as a screening biopsy or in response to the detection of dnDSA.

### Inclusion and exclusion criteria

Patients were included if they met the following criteria: i) aged at least 18 years, ii) had received a first or second ABO-compatible kidney transplant either from a deceased donor after brain death or circulatory death, or from a living donor, performed at least 90 days earlier, iii) presence of one or more dnDSAs, iv) evidence of microvascular inflammation (sum of glomerular inflammation (g score) and peritubular capillary inflammation (ptc score), g + ptc ≥2), and chronic glomerulopathy (cg score <3) on graft biopsy, and v) provided prior written informed consent. Female patients of childbearing potential were required to have a negative pregnancy test on the day of enrollment, and all participants (male and female) were required to use at least one effective contraceptive method before the initiation of treatment, throughout treatment and for the duration of the study. Patients with concomitant borderline lesions or T-cell-mediated rejection, corresponding to mixed rejection phenotypes, were eligible for inclusion.

Exclusion criteria were as follows: i) presence of a DSA before or at the time of transplantation, ii) patients receiving a third or fourth kidney transplant, iii) receiving combined transplantation with a non-renal organ, iv) previous history of AMR during the current transplantation, v) severe graft dysfunction, defined as an estimated GFR (eGFR) < 20 mL/min/1.73 m^2^, vi) severe histological lesions of chronic AMR, defined by the cg score of 3/3, vii) severe neuropathy or hematologic disturbances (platelet count <100 × 10^9^/L or absolute neutrophil count <1,000 × 10^6^/L), viii) active uncontrolled infection, chronic active hepatitis B or C, or HIV infection, ix) severe hepatic impairment or abnormal liver function tests, and x) acute diffuse infiltrative pneumonitis or pericardial involvement. Patients unable to understand or comply with the study protocol, as well as those enrolled in another therapeutic trial or within its exclusion period, were also excluded.

### Study intervention

All patients initially received five plasma exchange sessions within 10 days of inclusion. During this period, maintenance immunosuppression was adjusted, if necessary, to include prednisone (10 mg/day), mycophenolate mofetil (500 mg twice daily) or mycophenolate sodium (360 mg twice daily), and tacrolimus with target trough levels of 6–10 ng/mL (Table 1).

**TABLE 1:** Study treatment protocol.

Treatment component	Bortezomib group	Standard-of-care group
Plasma exchange	Five sessions within 10 days after inclusion	
Maintenance immunosuppression	Prednisone 10 mg/day; mycophenolate mofetil 500 mg twice daily or mycophenolate sodium 360 mg twice daily; tacrolimus (target trough level 6–10 ng/mL)	Same protocol, adjusted if necessary
Bortezomib	Two cycles, each consisting of four intravenous injections of 1.3 mg/m^2^ administered every 3 days, repeated after 3 weeks	-
Dexamethasone	20 mg orally on each day of bortezomib administration	20 mg orally, four doses every 3 days, repeated after 3 weeks
Intravenous immunoglobulin (IVIg)	Four infusions of 2 g/kg at 3-week intervals	
Infection prophylaxis	Cotrimoxazole and valaciclovir for 6 months after inclusion	
Treatment discontinuation	Temporary: neutropenia, thrombocytopenia, abnormal liver function tests, or neuropathy. Permanent: patient withdrawal, severe irreversible toxicity, or graft failure requiring dialysis	
Follow-up	12 months	

Patients in the bortezomib group received two cycles of intravenous bortezomib, each consisting of four injections (1.3 mg/m^2^ every 3 days), administered at three-week intervals. In addition, these patients received oral dexamethasone (20 mg) on each day of bortezomib administration and four IVIg infusions (2 g/kg body weight) at three-week intervals. Patients in the standard-of-care group received dexamethasone according to the same schedule as the bortezomib group (20 mg orally, four doses every 3 days, repeated after 3 weeks) along with four IVIg infusions (2 g/kg body weight) at three-week intervals. The planned follow-up duration was 12 months (Table 1).

All patients received prophylaxis with cotrimoxazole and valaciclovir for 6 months after inclusion to prevent *Pneumocystis jirovecii*, herpes simplex virus, and varicella-zoster virus infections. The bortezomib treatment was temporarily discontinued in the event of toxicity including neutropenia, thrombocytopenia, abnormal liver function tests and/or neuropathy. Permanent discontinuation was required in cases of voluntary withdrawal by the patient, severe irreversible toxicity and/or terminal graft dysfunction requiring return to dialysis.

### Outcome measures

The primary endpoint was a composite of two conditions assessed at 12 months: i) a >50% decrease from baseline in the mean fluorescence intensity (MFI) of the idDSA and, ii) stabilization or improvement of histological AMR lesions on the 12-month biopsy, defined as a difference in MVI score, defined as g + ptc, ≤1, and in cg score <1 compared with baseline. This composite endpoint was chosen to capture both immunological and histological responses to treatment.

Secondary endpoints included: i) each component of the primary endpoint analyzed separately, ii) in patients with multiple DSA, relative change from baseline in the MFI of each DSA and in the cumulative MFI, iii) change in DSA MFI after 6 months, iv) histological changes between baseline and 12-month biopsies, including acute and chronic lesions according to the latest Banff criteria, v) changes over 12 months in proteinuria (24-h excretion and urine protein/creatinine ratio), serum creatinine and eGFR (Modification of Diet in Renal Disease (MDRD) formula), and vi) patient and graft survival at 12 months. Safety was assessed throughout treatment and during the 12-month follow-up, with a focus on hematologic, hepatic, neurologic (including neuropathy) and infectious adverse events.

### Data collection

For histopathological assessment, all available baseline/screening and 12-month kidney biopsy slides were centrally reviewed at the pathology laboratory of Hôtel Dieu de Nantes by an expert renal pathologist blinded to treatment allocation. Slides were assessed using standard histological stains, including hematoxylin–eosin–saffron, periodic acid–Schiff, Masson’s trichrome, and silver staining, and lesions were graded according to updated Banff classification at the time of reading. Kidney biopsies were subsequently reclassified according to the 2022 Banff criteria using an automated method [15]. DSA were centrally assessed using Luminex single antigen (Labscreen One Lambda, Thermofisher) by immunologists blinded to treatment allocation. Investigators at each participating center collected clinical and transplant-related data, including sex, weight, rank of transplantation, donor type, dialysis vintage, local histological readings, local pre- and post-transplant HLA antibody assays, and concomitant immunosuppressive therapies.

### Sample size

The sample size calculation assumed primary endpoint rates of 5% in the standard-of-care group and 25% in the bortezomib group. With a two-sided alpha of 0.05% and 80% power, the planned sample size was 50 patients per group, or 100 patients in total. The planned inclusion period was 24 months. Despite multicenter recruitment and a 30-month extension of the inclusion period, the target sample size was not reached because of slow recruitment, reflecting the limited number of patients who met all eligibility criteria and were willing to participate. Recruitment was therefore closed at the end of the extended inclusion period after 60 patients had been randomized.

### Statistical analysis

The primary endpoint was compared in the intention-to-treat (ITT) population with missing data considered as treatment failures. A sensitivity analysis was also performed in the per-protocol (PP) population. Secondary endpoints were analyzed in the ITT population for the two components of the primary endpoint, and on available data (complete-case analysis) for the other endpoints. All statistical tests were two-tailed with a significance level of 5%.

Baseline clinical and biological characteristics were described by group. Quantitative variables were expressed as mean ± standard deviation (SD) or median [Q1:Q3] and qualitative variables as numbers and percentages (%). Comparisons of the primary endpoint and other categorical variables between the two groups were performed using Chi-squared or Fisher’s exact tests, as appropriate. Quantitative variables were compared using Student’s t-test or Wilcoxon’s rank-sum test when distributional assumptions were not met. Relative risks (RR) and 95% confidence interval (95% CI) were estimated using log-binomial regression models. Longitudinal outcomes were analyzed using linear mixed-effects models. Secondary analyses were considered exploratory and no adjustment for multiple comparisons was performed. Given the limited sample size, secondary endpoints were analyzed on available data without imputation. All analyses were performed with R software (www.r-project.org).

### Ethical considerations

This study was conducted in accordance with the Declaration of Helsinki. Approval was obtained from the *Comité de protection des personnes Ile de France II* (CPP) (Reference: 2014-03-01 RBM) and the *Agence Nationale de Sécurité du Médicament et des Produits de Santé* (ANSM) (Reference: 140368A-11). This trial was registered at ClinicalTrials.gov (NCT02201576) and an independent Data Safety Monitoring Board (DSMB) regularly reviewed the data to ensure patient safety and monitor the progress of the study.

## Results

### Baseline characteristics

Between February 12, 2015, and July 25, 2019, 60 patients were randomized. The planned sample size of 100 patients was not reached by the end of the extended recruitment period because of slower-than-anticipated enrolment related to the restrictive eligibility criteria. For the ITT analysis, 30 patients were allocated to each group. For the PP analysis, 20 patients were included in the bortezomib group and 19 in the standard-of-care group; one patient randomized to bortezomib did not receive the intervention and was therefore analyzed in the standard-of-care group for the PP analysis (Figure 1). Baseline characteristics are presented in Table 2. Patients in the bortezomib group were more frequently women (53.3% vs. 13.3%) and had a lower mean age (44.4 ± 13.8 vs. 51.3 ± 13.0 years). The median duration from transplantation to inclusion was 76 months in both groups. Notably, retransplantations occurred only in the bortezomib group (13.3%) (Table 2). A history of T-cell mediated rejection (TCMR) prior to inclusion was reported in one patient in the bortezomib group, and four in the standard-of-care group. Most patients received triple maintenance immunosuppression consisting of prednisone, ciclosporin or tacrolimus, and mycophenolate (Table 2). At inclusion, six patients in each group were not receiving a calcineurin inhibitor. AMR was more often subclinical than clinical for both groups (bortezomib group: 60.0% and standard-of-care group: 66.7%).

**FIGURE 1:**
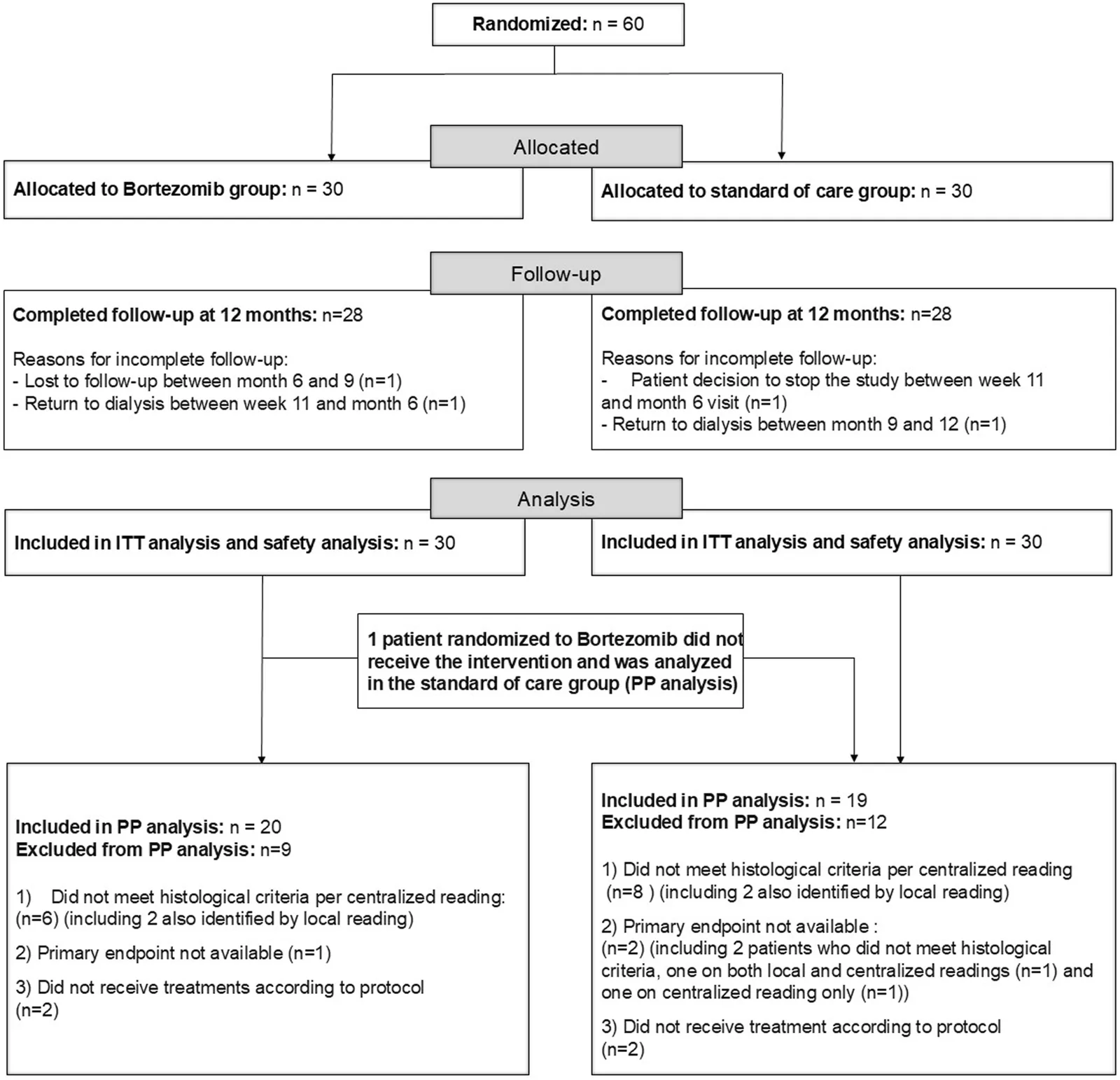
Study flowchart.

**TABLE 2:** Baseline characteristics.

Variable	Bortezomib group (n = 30)	Standard-of-care group (n = 30)
Sex		
Male	14 (46.7)	26 (86.7)
Female	16 (53.3)	4 (13.3)
Age at inclusion (years)	44.4 ± 13.8	51.3 ± 13.0
Type of nephropathy		
Diabetic	0 (0.0)	6 (20.0)
Hypertensive	1 (3.3)	2 (6.6)
Polycystic kidney disease	4 (13.3)	3 (10.0)
Pyelonephritis or chronic interstitial nephritis	4 (13.3)	3 (10.0)
Glomerulonephritis	11 (36.7)	11 (36.7)
Other	5 (16.7)	3 (10.0)
Unknown	5 (16.7)	2 (6.7)
Rank of transplantation		
First transplant	26 (86.7)	30 (100.0)
Second transplant	4 (13.3)	0 (0.0)
Duration at inclusion since transplantation (months)	76 [25: 104]	76 [30: 111]
Donor type		
Living	8 (26.7)	6 (20.0)
Deceased (brain death)	21 (70.0)	21 (70.0)
Deceased (circulatory death)	1 (3.3)	3 (10.0)
History of TCMR before inclusion	1 (3.3)	4 (13.3)
Renal function at inclusion		
Serum creatinine (µmol/L)	149.2 ± 61.0	149.7 ± 50.4
eGFR (mL/min/1.73 m^2^)	44.0 ± 14.6	47.5 ± 19.3
Urine protein/creatinine ratio (g/g)*	0.48 [0.11: 0.73]	0.23 [0.11: 1.20]
Maintenance immunosuppression at inclusion		
Corticosteroid (prednisone)	24 (80.0)	24 (80.0)
Ciclosporin	6 (20.0)	6 (20.0)
Tacrolimus	18 (60.0)	18 (60.0)
No ciclosporin or tacrolimus	6 (20.0)	6 (20.0)
Azathioprine	0 (0.0)	1 (3.3)
Mycophenolate^†^	30 (100.0)	27 (90.0)
No mycophenolate or azathioprine	0 (0.0)	2 (6.7)
Sirolimus	1 (3.3)	1 (3.3)
Everolimus	2 (6.7)	2 (6.7)
No sirolimus or everolimus	27 (90.0)	27 (90.0)

eGFR; estimated glomerular filtration rate, TCMR; T-cell-mediated rejection, SD; standard deviation. Data are presented as n (%) for categorical variables, mean ± SD, for normally distributed variables, or median [Q1: Q3] for non-normally distributed variables.

* Missing data for urine protein-to-creatinine ratio: bortezomib group n = 10 and standard-of-care group n = 4.

^†^ Data were unavailable for one patient in the standard-of-care group.

### Immunological and histological characteristics

At inclusion, patients in the bortezomib group more frequently had two or more dnDSA than in the standard-of-care (60.0% vs. 41.3%), with a median of 2 [1:3] DSA vs. 1 [1: 2] in the standard-of-care group, and they also had a higher proportion of class I DSA than in the standard-of-care group (whether considering the idDSA or all the DSA) (Table 3). Both the idDSA MFI and the sum of all the DSA MFI per patient were higher for patients in the bortezomib group.

**TABLE 3:** Characteristics of donor specific antibodies (DSA) at inclusion.

Variable	Bortezomib group (n = 30)	Standard-of-care group (n = 29*)
Number of dnDSA per patient, median [Q1:Q3]	2 [1: 3]	1 [1: 2]
Distribution of patients according to number of dnDSA detected, n (%)		
1	12 (40.0)	17 (58.6)
2	8 (26.6)	5 (17.2)
3	5 (16.7)	4 (13.8)
>3	5 (16.7)	3 (10.4)
Class of the idDSA		
*I*	8 (26.7)	3 (10.3)
*II*	22 (73.3)	26 (89.7)
Class of all DSA		
I	7 (23.3)	3 (10.3)
II	14 (46.7)	21 (72.4)
I+II	9 (30.0)	5 (17.3)
idDSA MFI	17,130 [4,121: 22,772]	11,234 [2,599: 22,549]
Sum of all the DSA MFI	22,440 [4,946: 29,851]	11,743 [2,599: 28,756]

DSA; donor specific antibodies, idDSA; immunodominant donor specific antibody, dnDSA; *de novo* DSA, MFI; mean fluorescence intensity. All values are according to central reading and are expressed as n (%) or median [Q1: Q3].

* Missing data for one patient in the standard treatment group.

Concerning biopsy findings at inclusion, discrepancies were present between local and central readings of AMR lesions (Table 4). According to the central assessment, only 25 patients (83.3%) in the bortezomib group and 24 patients (80.0%) in the standard-of-care group had MVI score ≥2, which was used as an inclusion criterion by local readings. Chronic glomerulopathy (cg score ≥1) was only reported in seven patients (23.3%) in each group, explaining the low median [IQR] score of 0[0:0]. According to the 2022 Banff criteria reclassification, active AMR was present in 18 patients (60.0%) in each treatment group. Chronic active AMR was present in seven patients (23.3%) in the bortezomib group and six patients (20.0%) in the standard-of-care group; one additional patient (3.3%) in the standard-of-care group had chronic inactive AMR. The remaining patients were classified as having C4d staining with DSA, probable AMR, or no AMR. Associated TCMR lesions were present in nine patients (30.0%) in the bortezomib group and five patients (16.7%) in the standard-of-care group (Table 4).

**TABLE 4:** Biopsy results at inclusion.

Variable	Bortezomib group (n = 30)	Standard-of-care group (n = 30)
Histological lesions		
Glomerular inflammation (g score)		
Score ≥ 1	19 (63.3)	21 (70.0)
Score value	1.0 [0.0: 2.0]	1.0 [0.0: 2.0]
Peritubular capillary inflammation (ptc score)		
Score ≥ 1	23 (76.7)	23 (76.7)
Score value	2.0 [1.3: 3.0]	2.0 [2.0: 3.0]
MVI score		
Score ≥ 2	25 (83.3)	24 (80.0)
Score value	3.0 [2.0: 4.0]	3.0 [2.3: 4.8]
Chronic glomerulopathy (cg score)		
Score ≥ 1	7 (23.3)	7 (23.3)
Score value	0.0 [0.0: 0.0]	0.0 [0.0: 0.0]
C4d staining (positive)	17 (56.7)	15 (50.0)
Vascular inflammation (v score)		
Score ≥ 1	3/29 (10.3)	1/29 (3.4)
Tubular atrophy (ct score)	1.0 [1.0: 1.8]	1.0 [1.0: 1.0]
Interstitial fibrosis (ci score)	1.0 [0.0: 1.8]	0.0 [0.0: 1.0]
IFTA grade≥1	24/30 (80.0)	20/29 (69.0)
Banff 2022 diagnosis*		
AMR		
*Active*	18 (60.0)	18 (60.0)
Chronic active	7 (23.3)	6 (20.0)
Chronic inactive	0 (0.0)	1 (3.3)
C4d staining only+DSA	1 (3.3)	0 (0.0)
Probable	0 (0.0)	4 (13.4)
None	4 (13.4)	1 (3.3)
Associated TCMR		
Borderline Changes	4 (13.3)	2 (6.7)
Acute (grade I and above)	1 (3.3)	2 (6.7)
Chronic active	4 (13.3)	1 (3.3)

MVI score: microvascular inflammation corresponding to g+ptc sum score; DSA; donor specific antibodies, IFTA; interstitial fibrosis and tubular atrophy, AMR; antibody-mediated rejection, TCMR; T-cell-mediated rejection. All values are according to central reading and are expressed as n (%) or mean ± SD or median [Q1: Q3].

* Reclassified according to the 2022 Banff criteria [15].

### Combined primary efficacy endpoint

In both the ITT and PP analyses, the primary endpoint rate did not significantly differ between groups. In the ITT analysis, the endpoint was achieved by 40.0% of patients in the bortezomib group and 33.3% in the standard-of-care group (RR = 1.20 [95% CI: 0.61 to 2.34], p = 0.59). Missing primary endpoint data were considered treatment failure in four patients in the standard-of-care group and one in the bortezomib group. In the PP analysis, the corresponding rates were 50.0% and 42.1% (RR = 1.19 [95% CI: 0.60 to 2.36], p = 0.62) (Table 5). No significant interaction was observed between treatment allocation and rejection type (clinical or subclinical; p = 0.86).

**TABLE 5:** Primary and secondary efficacy endpoint at 12 months.

Endpoint (each one assessed at 12 months)	Patients		RR [95% CI]*	Absolute difference [95% CI]*	p-value
	Bortezomib group	Standard-of-care group			
Primary endpoint					
ITT analysis	12/30 (40.0)	10/30 (33.3)	1.20 [0.61 to 2.34]	6.7 [-17.6 to 30.5]	0.59
PP analysis	10/20 (50.0)	8/19 (42.1)	1.19 [0.60 to 2.36]	7.9 [-23.1 to 37.8]	0.62
Secondary endpoints					
>50% reduction in idDSA MFI (ITT analysis)	17/30 (56.7)	14/30 (46.7)	​	​	0.44
Stabilization or improvement of histological AMR lesions (ITT analysis)	17/30 (56.7)	17/30 (56.7)	​	​	1.00
>50% reduction in idDSA MFI (PP analysis)	13/20 (65.0)	11/19 (57.9)	​	​	0.65
Stabilization or improvement of histological AMR lesions (PP analysis)	13/19 (68.4)	12/18 (66.7)	​	​	0.91
Estimated slope between baseline and 12 months					
Serum creatinine (µmol/L)	45.9 ± 13.5	29.7 ± 13.5	​	16.14 [-21.5 to 53.8]	0.40
eGFR (mL/min/1.73 m^2^)	−7.10 ± 2.19	−4.68 ± 2.18	​	−2.42 [-8.51 to 3.67]	0.43
Log-transformed PCR (g/g)	0.20 ± 0.23	0.11 ± 0.23	​	0.09 [-0.55 to 0.74]	0.78
Graft survival	29/30 (96.7)	29/30 (96.7)	​	​	1.00
Patient survival	30/30 (100.0)	30/30 (100.0)	​	​	​

ITT; intention to treat, PP; per protocol, DSA; donor specific antibodies, idDSA; immunodominant donor specific antibodies, dnDSA; *de novo* DSA, MFI; mean fluorescence intensity, RR; risk ratio, CI; confidence interval, SD; standard deviation. All values are according to central reading and are expressed as n/N (%) or mean ± SD.

* Standard-of-care group used as the reference.

When separately analyzed, neither component of the combined endpoint was different between groups. After 12 months, a >50% reduction in idDSA MFI was observed in 56.7% of patients in the bortezomib group and 46.7% of patients in the standard-of-care group in the ITT analysis (p = 0.44), and 65.0% and 57.9% in the PP analysis (p = 0.65), respectively. Stabilization or improvement of histological lesions at month 12 was reached in 56.7% of patients in both groups in the ITT analysis (p = 1.00), and 68.4% and 66.7% of patients in the PP analysis, respectively (p = 0.91). Thus, 5/28 (17.8%, bortezomib) and 2/25 (8.0%, standard-of-care) of patients experienced a >50% decrease in idDSA MFI without improving or stabilizing MVI, and 5/28 (17.8%, bortezomib) and 7/25 (28.0%, standard-of-care) improved or stabilized MVI without significantly reducing idDSA MFI.

In a *post hoc* analysis of patients with baseline idDSA MFI >15,000, the primary composite endpoint was achieved in 42.1% of patients in the bortezomib group and 8.3% in the standard-of-care group (p = 0.10). A >50% reduction in idDSA MFI was observed in 52.6% and 16.7% of patients, respectively (p = 0.065) (Supplementary Table S1).

In separate sensitivity analyses adjusted individually for baseline characteristics that were imbalanced between groups despite randomization (sex, transplantation rank, idDSA class, and baseline idDSA MFI), the association between treatment allocation and the primary endpoint remained non-significant (Supplementary Table S2).

### Changes in renal function, DSA and MVI

Changes in serum creatinine, eGFR, and urine protein/creatinine ratio between baseline and 12 months did not significantly differ between patients in the bortezomib and standard-of-care groups (Table 5; Figure 2). No deaths occurred, and one patient in each group returned to dialysis.

**FIGURE 2:**
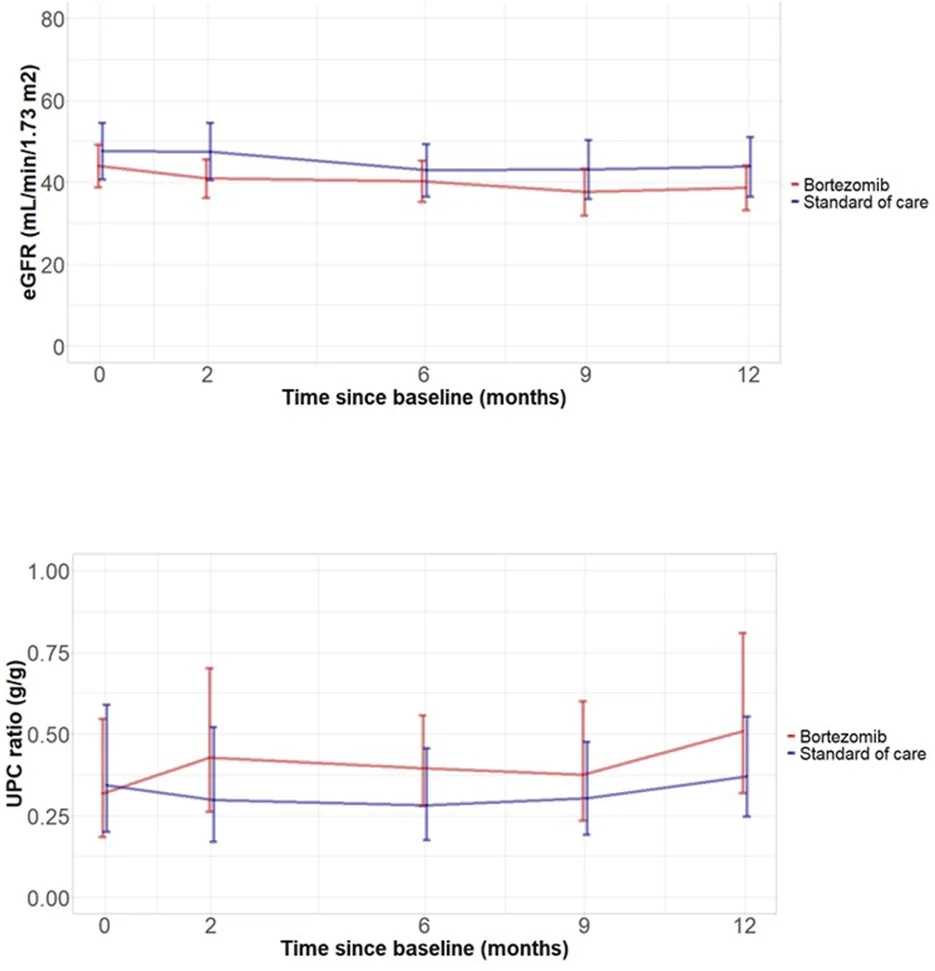
Change in renal function and proteinuria from baseline to 12 months in the bortezomib and standard-of-care group. eGFR; estimated glomerular filtration rate (Modification of Diet in Renal Disease (MDRD) formula), UPC; urine protein/creatinine (g/g). Control corresponds to the standard-of-care group.

For patients receiving bortezomib, the idDSA MFI decreased by 45.1% [-69.7: -6.1] after 6 months (n = 23), and by 60.2% [-80.6: -11.5] after 12 months (n = 28). Similar reductions were observed for patients in the standard-of-care group: 50.7% [-76.6: -6.6] after 6 months (n = 27; p = 0.92) and 60.0% [-81.3: -11.5] after 12 months (n = 27; p = 0.85) (Figure 3). Median relative change in the sum of all DSA MFI at 12 months was also comparable: 60.3% [-74.3: -27.6] in the bortezomib group (n = 28) and 57.6% [-81.8: -21.5] in the standard-of-care group (n = 27; p = 0.80).

**FIGURE 3:**
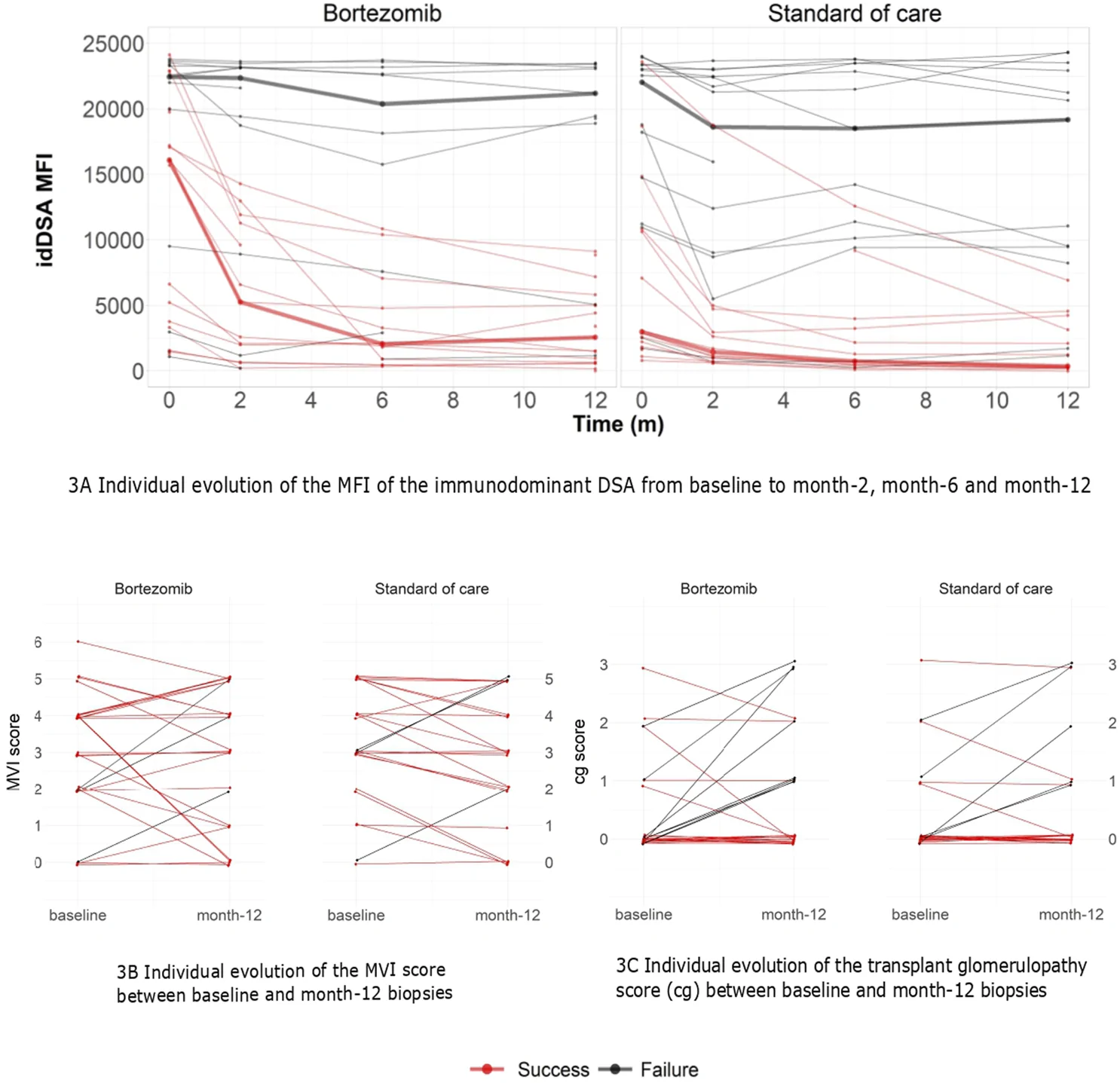
Individual changes in donor-specific antibodies (DSA) and histological lesions from baseline to 12 months for patients in the bortezomib and standard-of-care group. idDSA; immunodominant donor-specific antibodies, MFI; mean fluorescence intensity, MVI; microvascular inflammation score, cg; transplant glomerulopathy score. **(A)** Individual changes in idDSA MFI over 12 months. Red curves represent patients with immunological success (reduction in idDSA MFI >50%), and black curves represent patients with immunological failure. The thicker lines represent the median values of the responder and non-responder subgroups. **(B)** Individual changes in microvascular inflammation (MVI score) between baseline and 12 months **(C)** Individual changes in transplant glomerulopathy (cg score) between baseline and 12 months. Red curves represent patients with histological success (ΔMVI ≤1 and Δcg <1), and black curves represent patients with histological failure.

Patients whose idDSA MFI dropped by at least half at 12 months (responders) had lower baseline MFI values than non-responders: median 16,080 vs. 22,582 in the bortezomib group and 2,996 vs. 22,054 in the standard-of-care group.

Median scores of histological lesions remained stable between baseline and 12 months in both groups (Table 6). Histological failure was due to the progression of MVI (Δg + ptc score ≥2) in three patients in each group and of transplant glomerulopathy (Δcg score ≥1) in eight patients in the bortezomib group and five patients in the standard-of-care group (Figure 3). One patient in each group had progression of both MVI and cg scores, while most with cg progression showed no or only mild worsening of MVI. Among patients in the ITT population with an MVI score ≥2 at baseline and available 12-month histological data, nine of 23 patients in the bortezomib group and 10 of 20 patients in the standard-of-care group showed a decrease in MVI score between baseline and 12 months. Complete MVI resolution, defined as an MVI score of 0 at 12 months, occurred in 3/23 patients (13.0%) and 2/20 patients (10.0%), respectively. In the PP analysis, complete MVI resolution occurred in 3/20 patients (15.0%) and 2/17 patients (11.8%), respectively. The cg score decreased in three patients in the bortezomib group and two patients in the standard-of-care group (Table 6; Figure 3).

**TABLE 6:** Histological lesion findings at baseline and at 12 months.

Variable	Bortezomib group (n = 27)	Standard-of-care group (n = 24)	p-value
Histological lesions			
Glomerular inflammation (g score)			
Baseline	1.0 [0.0: 2.0]	1.0 [0.0: 2.0]	​
12 months	1.0 [0.0: 2.0]	1.0 [0.0: 2.0]	0.98
≥1 at 12 months	17 (63.0)	15 (62.5)	0.97
Change between baseline and 12 months	0.0 [0.0: 0.0]	0.0 [-1.0: 0.0]	0.57
Peritubular capillary inflammation (ptc score)			
Baseline	2.0 [1.5: 3.0]	2.0 [2.0: 3.0]	​
12 months	2.0 [1.0: 3.0]	2.0 [1.5: 3.0]	0.87
≥1 at 12 months	21 (77.8)	18 (75.0)	0.82
Change between baseline and 12 months	0.0 [-0.5: 1.0]	0.0 [-0.3: 0.0]	0.41
MVI score			
Baseline	3.0 [2.0: 4.0]	3.0 [2.8: 4.3]	​
12 months	3.0 [1.0: 4.5]	3.0 [2.0: 4.3]	0.91
≥2 at 12 months	19 (70.4)	19 (79.2)	0.47
Change between baseline and 12 months	0.0 [-1.0: 1.0]	0.0 [-1.0: 0.0]	0.35
Decrease in the MVI score at 12 months*, n/N (%)	9/23 (39.1)	10/20 (50.0)	​
MVI = 0 at 12 months (ITT population), n/N (%)	3/23 (13.0)	2/20 (10.0)	​
MVI = 0 at 12 months (PP population), n/N (%)	3/20 (15.0)	2/17 (11.8)	​
Chronic glomerulopathy (cg score)			
Baseline	0.0 [0.0: 0.5]	0.0 [0.0: 0.3]	​
12 months	0.0 [0.0: 1.0]	0.0 [0.0: 1.0]	0.62
≥1 at 12 months	11 (40.7)	8 (33.3)	0.58
Change between baseline and 12 months	0.0 [0.0: 1.0]	0.0 [0.0: 0.0]	0.69
Decrease in the cg score for patients with cg > 0 at inclusion	3/7	2/6	​
C4d staining (positive)			
≥1 at baseline	16 (59.3)	12 (50.0)	​
≥1 at 12 months	7 (25.9)	7 (29.2)	0.80
Change between baseline and 12 months	0.0 [-2.0: 0.0]	0.0 [-1.0: 0.0]	0.29
Vascular inflammation (v score)			
≥1 at baseline	3/25 (12.0)	1/23 (4.3)	​
≥1 at 12 months	1/25 (4.0)	0/23 (0.0)	1
Change between baseline and 12 months	0.0 [0.0: 0.0]	0.0 [0.0: 0.0]	0.73
Tubular atrophy (ct score)			
Baseline	1.0 [1.0: 1.0]	1.0 [1.0: 1.0]	​
12 months	1.0 [1.0: 1.5]	1.0 [1.0: 1.3]	0.31
Change between baseline and 12 months	0.0 [0.0: 0.5]	0.0 [0.0: 0.0]	0.74
Interstitial fibrosis (ci score)			
Baseline	1.0 [0.0: 1.0]	0.0 [0.0: 1.0]	​
12 months	1.0 [0.0: 1.5]	1.0 [0.0: 2.0]	0.98
Change between baseline and 12 months	0.0 [-1.0: 0.5]	0.0 [0.0: 1.0]	0.39
IFTA grade≥1			
≥1 at baseline	21 (77.8)	16/23 (69.6)	​
≥1 at 12 months	18 (66.7)	16/23 (69.6)	0.83
Change between baseline and 12 months	−1.0 [-1.5: 0.0]	0.0 [-2.0: 1.0]	0.66
Banff 2022 diagnosis^†^			
AMR, n (%)			
Active	8 (29.6)	11 (45.8)	​
Chronic active	11 (40.7)	8 (33.3)	​
Chronic inactive	8 (29.6)	5 (20.8)	​
Associated TCMR, n (%)			
Borderline Changes	0 (0.0)	1 (4.2)	​
Acute (grade I and above)	1 (3.7)	0 (0.0)	​
Chronic active	0 (0.0)	0 (0.0)	​

MVI score: microvascular inflammation corresponding to g+ptc sum score; IFTA; interstitial fibrosis and tubular atrophy, AMR; antibody-mediated rejection, TCMR; T-cell-mediated rejection. All values are according to central reading and are expressed as n (%) or median [Q1: Q3]. Comparisons were performed using the Wilcoxon or Chi-squared tests as appropriate.

* Among patients with MVI score ≥2 at baseline.

^†^ Reclassified according to the 2022 Banff criteria (Yoo et al., 2023).

In the bortezomib group, tubulitis with a Banff t score ≥1 was present in 29.6% of patients at baseline and 7.4% at 12 months, compared with 20.8% and 4.2%, respectively, in the standard-of-care group. Interstitial inflammation with a Banff i score ≥1 was present in 19.0% and 7.0% of patients in the bortezomib group and in 21.0% and 8.0% of patients in the standard-of-care group at baseline and 12 months, respectively. Interstitial fibrosis and tubular atrophy (IFTA) scores worsened in six patients in the bortezomib group and eight patients in the standard-of-care group, which was associated with a cg or MVI progression in two patients in each group. According to the 2022 Banff classification at the 12-month biopsy, 19/27 patients in the bortezomib group and 19/24 patients in the standard-of-care group still had active or chronic active AMR (Table 6).

### Adverse events and safety of bortezomib

Overall, 132 adverse events were reported in the bortezomib group and 118 in the standard-of-care group, with infections, neurological and gastrointestinal events being the most frequent in both groups. In the bortezomib group, 11 patients discontinued or did not complete the two planned treatment courses due to hematological, neurological or hepatic toxicity. Twenty-three patients in the bortezomib group (76.7%) experienced at least one serious adverse event compared to 14 (46.7%) in the standard-of-care group (p = 0.017), corresponding to 43 and 30 serious adverse events, respectively (Table 7). No significant between-group differences were observed in the proportions of patients experiencing adverse events or serious adverse events within individual System Organ Class categories. Infections, including urinary tract and viral infections were the most common serious adverse events in both groups.

**TABLE 7:** Serious adverse events and adverse events in patients receiving bortezomib and standard-of-care treatment.

Type of adverse events	Bortezomib group, n = 30	Standard-of-care group, n = 30	p-value
Patients with at least one serious adverse event, n (%)	23 (76.7)	14 (46.7)	0.017
Patients with at least one adverse event, n (%)	24 (80.0)	26 (86.7)	0.49
Patients with at least one serious adverse event, by SOC, n (%)	​	​	​
Infections	8 (26.7)	7 (23.3)	0.77
Urinary tract infection	4	1	​
Respiratory infection	1	0	​
Viral infection	4	2	​
Bacterial infection	1	5	​
Cardiac	0 (0)	4 (13.3)	0.11
Heart failure	0	4	​
Blood and lymphatic disorders	6 (20.0)	1 (3.3)	0.10
Thrombocytopenia	4	0	​
Neutropenia	2	0	​
Anemia	0	1	​
Renal	5 (16.7)	1 (3.3)	0.19
Acute kidney injury	4	1	​
Gastro-intestinal	3 (10)	0 (0)	0.24
Hepatobiliary	5 (16.7)	0 (0)	0.052
Nervous system disorders	3 (10)	0 (0)	0.24
Metabolic	2 (6.7)	2 (6.7)	>0.99
Patients with at least one adverse event, by SOC, n (%)	​	​	​
Infections	16 (53.3)	11 (36.7)	0.19
Urinary tract infection	3	4	​
Viral infection	4	1	​
Cardiac	2 (6.7)	4 (13.3)	0.67
Hypertension	2	0	​
Cardiac failure	0	1	​
Hematologic	7 (23.3)	8 (26.7)	0.77
Anemia	5	3	​
Thrombocytopenia	0	3	​
Renal	7 (23.3)	4 (13.3)	0.32
Acute kidney injury	3	2	​
Proteinuria	3	0	​
Gastro-intestinal	13 (43.3)	8 (26.7)	0.18
Diarrhea	6	5	​
Nausea, vomiting	10	3	​
Hepatobiliary	2 (6.7)	2 (6.7)	>0.99
Nervous system	15 (50.0)	14 (46.7)	0.80
Paresthesia	8	6	​
Headache/migraine	7	4	​
Metabolism	7 (23.3)	5 (16.7)	0.52
Gout, hyperuricemia	2	4	​
Diabetes, hyperglycemia	2	2	​
General disorders	5 (16.7)	7 (23.3)	0.52
Asthenia	4	2	​

SOC, System Organ Class

## Discussion

In this multicenter trial, we found that the addition of bortezomib to standard-of-care did not improve efficacy compared with standard-of-care alone in patients with active AMR associated with dnDSA. No significant differences were observed between groups in the individual immunological and histological efficacy endpoints or renal function parameters. This study focused on late rejection, with a median time from transplantation to inclusion of 76 months.

Randomized trials in AMR are rare [16–19]. A major methodological strength of our study was the blinded central assessment of both DSA and histological lesions. The only difference between the two treatment groups was the addition of bortezomib. We selected this design because proteasome inhibitors target antibody-secreting cells, including plasmablasts and plasma cells, thereby reducing antibody production. In the context of AMR, they may need to be combined with strategies that rapidly remove or neutralize circulating pathogenic antibodies, such as plasma exchange and intravenous immunoglobulins. Corticosteroids were also included because they may enhance the pro-apoptotic effects of proteasome inhibitors on plasma cells. Given the poor prognosis of late AMR and the relatively recent occurrence of dnDSA in most patients in our study, the combination of corticosteroids, plasma exchange, and IVIg was considered standard-of-care at the time of the study and was subsequently recommended by the Transplantation Society Working Group consensus in 2020 [20], although some experts now recommend avoiding these treatments in late AMR, in favor of anti-CD38 monoclonal antibodies alone [16].

Previous uncontrolled studies have reported contradictory findings regarding the effect of bortezomib in patients with AMR and DSA. When used alone, bortezomib appeared to have little or no effect on DSA [12, 13], whereas a transient but significant reduction in DSA was observed when bortezomib was combined with IVIg, corticosteroids, plasma exchange, and sometimes rituximab [10, 11]. However, the effects on AMR outcomes were not assessed in these studies.

In the BORTEJECT study, 44 patients with DSA and late AMR were randomized to receive bortezomib or placebo, with no benefit in preserving eGFR [14, 17]. Despite these negative results, there remained a rationale for evaluating bortezomib in combination with other therapies and in a more homogeneous population of patients. The BORTEJECT study included patients with both preformed DSA and dnDSA. In contrast, our study specifically focused on patients with dnDSA, and combined bortezomib with corticosteroids, plasma exchange, and IVIg to achieve a rapid and sustained effect on DSA, while maximizing the effect of proteasome inhibition on plasma cells. Direct comparison between the two studies is difficult because of differences in patient populations, baseline DSA characteristics, treatment regimens, and follow-up duration. Baseline idDSA MFI was substantially lower in the BORTEJECT study than in the two groups of the TRIBUTE study, and the extent of MFI reduction appeared to be lower in BORTEJECT. Although median MVI scores on biopsies performed at 2 years remained stable, more patients developed cg lesions in BORTEJECT than in TRIBUTE (69% versus 40%).

Few controlled trials have recently been published in late AMR. Anti-CD38 monoclonal antibodies are of interest because they target plasma cells, like bortezomib, but also NK cells involved in AMR lesions through antibody-dependent cellular cytotoxicity. Mayer et al. recently reported a randomized trial evaluating the anti-CD38 antibody felzartamab versus placebo in late AMR. Felzartamab significantly reduced MVI but produced only a slight reduction in DSA MFI, suggesting a direct effect on inflammatory cells involved in MVI lesions [21]. Effects on circulating NK cells and NK cell-related AMR genes were also demonstrated in an ancillary study [22]. At 1 year, 4/11 patients (36%) treated with felzartamab had an MVI score of 0, compared with 13% of patients treated with bortezomib in the TRIBUTE study.

Anti-IL-6 antibodies have also generated considerable interest because of their expected inhibitory effects on T cells, B cells, and plasma cells. In a phase 2 placebo-controlled trial, clazakizumab showed effects on DSA levels, molecular AMR activity on biopsy, and the slope of eGFR decline in patients with late chronic active AMR, but no significant effect on MVI. Safety concerns were observed because several patients discontinued treatment due to serious infections or diverticulitis [23]. Another phase 2 trial of clazakizumab showed similar effects without the same safety concerns, but the subsequent phase 3 IMAGINE study was terminated early because of insufficient efficacy on renal function [24].

In the VIPAR study, patients with chronic active AMR were randomized to receive six monthly IVIg infusions or no IVIg. Patients in the IVIg group showed stabilization of chronic histological lesions and eGFR at 1 year, whereas those in the no-IVIg group experienced worsening renal function and histological injury. Compared with the TRIBUTE study, there was no substantial change in MVI score during the 12-month study period in either group, despite a reduction in mean idDSA MFI with IVIg between inclusion and month-12 [25].

Taken together, these studies demonstrate a dissociation between changes in DSA, MVI, and graft function in patients treated for late AMR. In our study, some patients showed improvements in both MVI and DSA MFI, whereas others showed discordant immunological and histological responses. This raises the important question of whether treatments targeting antibodies and MVI should be combined. Anti-CD38 antibodies appear to have a marked but potentially transient effect on MVI, possibly because high-level DSA may continue to circulate and stimulate antibody-dependent cellular cytotoxicity.

The observed discordance also raises questions regarding the sensitivity of the chosen composite primary endpoint. Although the endpoint was prespecified to capture both immunological and histological responses, combining these biologically distinct components may have reduced its ability to detect a treatment effect. Nevertheless, monitoring DSA remains clinically relevant when DSA are considered to contribute to MVI, particularly when treatment includes antibody removal, as in our trial [16]. In addition, transplant glomerulopathy generally evolves slowly, and including stabilization of the cg score within a 12-month endpoint may have further limited its sensitivity, although changes in cg score were observed in some patients.

Our study had several limitations. First, the study was underpowered because the planned sample size was not reached, and a modest treatment effect may therefore have been missed. In the exploratory *post hoc* analysis, patients with a very high baseline idDSA MFI (>15,000) more frequently achieved the composite primary endpoint and a >50% reduction in idDSA MFI in the bortezomib group than in the standard-of-care group. However, these differences were not statistically significant, possibly because of the small subgroup size and limited statistical power. This analysis was not prespecified and should therefore be interpreted cautiously.

The lack of efficacy may also reflect compensatory humoral mechanisms whereby bortezomib-induced plasma-cell depletion is followed by expansion of germinal-center B cells and follicular helper T cells in lymph nodes, as reported by Kwun et al. [26]. Additionally, Woodle et al. observed that subsets of bone-marrow plasma cells may develop resistance to proteasome inhibitors through increased immunoproteasome activity [27].

Despite randomization, the two groups were imbalanced, and patients in the bortezomib group had a higher immunological risk, with higher proportions of women and retransplantations, higher baseline idDSA MFI, and more class I DSA. However, in separate sensitivity analyses adjusted individually for these characteristics, the association between treatment allocation and the primary endpoint remained non-significant. The open-label design may also have introduced reporting bias, particularly for subjective adverse events, although the central assessments of DSA and histological outcomes were performed blinded to treatment allocation.

Treatment exposure was limited by toxicity, with 11 of 30 patients discontinuing or not completing the planned bortezomib regimen. This safety profile, together with the absence of a significant efficacy benefit, limits the clinical applicability of bortezomib in late active AMR and supports careful patient selection in any future evaluation, particularly among patients with comorbidities or increased susceptibility to infectious, hematological, neurological, or hepatic toxicity.

Finally, central review of the immunological and histological endpoints identified a substantial number of patients who no longer fulfilled the histological inclusion criteria: six in the bortezomib group and eight in the standard-of-care group. This further decreased the already limited number of evaluable patients in the PP population.

In conclusion, adding bortezomib to standard-of-care did not demonstrate a significant efficacy benefit in patients with late active AMR associated with dnDSA and was associated with a higher incidence of serious adverse events. Although the exploratory findings in patients with high baseline idDSA MFI warrant further investigation, the present results do not support the routine addition of bortezomib in this setting. Future therapeutic strategies should aim to address both circulating DSA and tissue-level inflammation while maintaining an acceptable safety profile.

## Data Availability

The datasets presented in this article are not readily available because of French data protection regulations governed by the Commission nationale de l’informatique et des libertés (CNIL). Biological samples collected during the study will be stored for up to 30 years at the Tissuthèque DNA/RNA biobank located in the Department of Pathology at Necker Children’s Hospital (Paris, France), under the responsibility of the coordinating investigator and site manager. Requests to access the datasets should be directed to Assistance Publique–Hôpitaux de Paris (AP-HP) and the Delegation for Clinical Research and Innovation (DRCI), secretariat-direction.drc@aphp.fr.

## Supplementary material

SUPPLEMENTARY DATA SHEET 1 Tribute study protocol Version n°10 2019-08-08.
