# Supplementary material for: Bortezomib in combination with standard-of-care for late active antibody-mediated rejection with de novo donor-specific antibodies after kidney transplantation: a multicenter randomized trial
Source: Transpl Int. 2026 Sep 23;39:16702. doi: 10.3389/ti.2026.16702 (PMC13642227; doi:10.3389/ti.2026.16702)
Supplement: Supplementary file 1 [file DataSheet1.PDF]

TRIBUTE study

Version 10.0 of 08/08/2019

Bortezomib for the treatment of chronic active antibody-mediated rejection in kidney transplantation

Project code: P120119/EudraCT: 2013-002322-23

**Sponsor**	***Assistance Publique des Hôpitaux de Paris***<br>Clinical Research and Innovation Department - Hôpital Saint Louis<br>1 avenue Claude Vellefaux<br>75475 Paris Cedex 10<br>Project Manager: **Sophie Courtial-Destembert**<br>Tel: +33 (0)1 40 27 55 91/Fax: +33 (0)1 44 84 17 01<br>sophie.courtial-destembert@aphp.fr
**Coordinating Investigator**	***Dr. Renaud Snanoudj***<br>Nephrology Department<br>Hôpital Foch<br>40 rue Worth<br>92150 Suresnes<br>Tel: +33 (0)1 46 25 30 95/Fax: +33 (0)1 46 25 28 79<br>r.snanoudj@hopital-foch.com
**Scientific Director**	***Professor Christophe Legendre***<br>Adult Renal Transplant Department, Hôpital Necker, 149 rue de Sèvres,<br>75015 Paris<br>Tel: +33 (0)1 44 49 54 32/Fax: +33 (0)1 44 49 54 30<br>christophe.legendre@aphp.fr
**Clinical Research Unit/Clinical Investigation Center Necker-Cochin, Hôpital Necker**	***Professor Jean Marc Treluyer***<br>149 rue de Sèvres<br>75743 Paris Cedex 15<br>Project Manager: **Sandra Colas**<br>Tel: +33 (0)1 71 19 64 32/Fax: +33 (0)1 44 49 59 50<br>sandra.colas@aphp.fr
**Biostatistician/Methodologist**	***Dr. Caroline Elie***<br>Clinical Research Unit/Clinical Investigation Center Necker Cochin<br>149 rue de Sèvres<br>75743 Paris Cedex 15<br>Tel: +33 (0)1 44 49 46 07/Fax: +33 (0)1 44 49 59 50<br>caroline.elie@aphp.fr
**General Agency for Healthcare Equipment and Products (AGEPS) (AGEPS), AP-HP**	***Dr. Blandine Lehmann, Chrystelle Frappesauce*** Clinical Trial Department<br>7 rue du Fer à Moulin, 75005 Paris<br>Tel: +33 (0)1 46 69 14 02/Fax: +33 (0)1 46 69 14 09<br>blandine.lehmann@aphp.fr<br>chrystelle.frappesauce-hereng@eps.aphp.fr

SIGNATURE PAGE for the biomedical research protocol, signed by the COORDINATING INVESTIGATOR and the SPONSOR'S REPRESENTATIVE

Title **TRIBUTE: "Bortezomib for the treatment of chronic active antibody-mediated rejection in kidney transplantation"**

Biomedical Research Code: **P120119**

Eudract 2013-002322-23

Version 10.0 of 08/08/2019

• **Coordinating investigator:**

Dr. Renaud Snanoudj
Nephrology Department
Hôpital Foch
40 rue Worth
92150 Suresnes

Date: 24.09.19
[Signature]

[Stamp:
Dr. Renaud Snanoudj
Nephrologie-Transplantation
Hôpital Foch
40 rue Worth
92150 Suresnes Cedex

• **Sponsor:**

Florence Favrel-Feuillade (Director of the Clinical Research and Innovation Department)
Assistance Publique-Hôpitaux de Paris
Clinical Research and Innovation Department
Hôpital Saint Louis
1 avenue Claude Vellefaux
75019 Paris

Date: 09 JULY 2020
[Signature]

[Stamp:
Serge Bureau
DRCI – AP-HP
Pôle Promotion

NB: This version corresponds to the text of the protocol and appendices sent to the REC and the competent authority, respectively, for review and authorization, as well as to other research partners (hospital directors, etc.).
The initial favorable opinion of the Research Ethics Committee (REC) IDF 2 - Necker was obtained on 07/07/2014 and the initial authorization from the ANSM was obtained on 05/06/2014.

TABLE OF CONTENTS

1. LITERATURE REVIEW, PRELIMINARY DATA, SCIENTIFIC RATIONALE, AND GENERAL DESCRIPTION OF THE STUDY 11

1.1. Definition of chronic active antibody-mediated rejection in kidney transplantation **11**

1.1.1. Clinical circumstances of chronic active antibody-mediated rejection 11

1.1.2. Diagnostic criteria for chronic antibody-mediated rejection 11

1.1.3. Incidence and adverse prognostic significance of chronic active AMR and so-called "de novo" DSA in kidney transplantation 12

1.2. Treatments used in the prevention and management of antibody-mediated rejection 14

1.2.1. Treatments for acute antibody-mediated rejection 14

1.2.2. Treatment for chronic antibody-mediated rejection 16

1.2.3. Failure of current antibody-targeted therapies: evidence for the role of plasma cells in anti-HLA antibody production? 16

1.3. Rationale for using a bortezomib-based strategy for the treatment of chronic active AMR 17

1.3.1. Description and role of the ubiquitin-proteasome system 17

1.3.2. Proteasome inhibition and immune response 18

1.3.3. The use of bortezomib as a proteasome inhibitor – Use in multiple myeloma and AL amyloidosis in combination with dexamethasone 18

1.3.4. Pre-clinical data on the use of bortezomib in transplantation 18

1.3.5. Clinical data on the use of bortezomib in kidney transplant patients 20

1.4. Description of the new strategy 22

1.5. Summary of the benefits and, where applicable, the foreseeable and known risks for individuals participating in the study 23

1.5.1. Individual benefits 23

1.5.2. Collective benefit 24

1.5.3. Risks 24

2. STUDY OBJECTIVES 29

2.1. Primary objective 29

2.2. Secondary objectives 29

3. SELECTION AND EXCLUSION OF STUDY PARTICIPANTS 29

3.1. Inclusion criteria 29

3.2. Exclusion criteria 30

4. STUDY DESIGN 31

4.1. Description of the study methodology 31

4.2. Patient recruitment 31

4.3. Patient selection/inclusion and follow-up 32

4.3.1. Selection/inclusion (D-10) - Randomization 32

4.3.2. Patient follow-up 32

4.3.3 Procedures and tests performed as part of standard care 33

4.3.4 Procedures and tests performed as part of the study 34

4.4. RANDOMIZATION METHOD 37

4.5. SITE OF EXAMINATIONS, SAMPLE COLLECTION, AND ASSAYS 37

4.5.1 Centralized tests and assays: 37

4.5.2 Local tests and assays: 37

4.6. Sample storage 37

4.6.1 Samples included in the Kidney Transplantation Biocollection 37

4.6.2 Samples taken for the pharmacokinetic study 38

4.7. Study duration ... 38
4.8. Identification of collected data ... 38
5. TREATMENTS ADMINISTERED TO STUDY PARTICIPANTS ... 39
5.1. Description of the bortezomib treatment required to conduct the study: Description of bortezomib (Velcade®) ... 39
5.2. Composition, dosage, and administration of the bortezomib (Velcade®) treatment ... 39
5.3. Description of the combination therapies in the experimental arm (bortezomib) and the control arm ... 42
5.3.1 Treatment regimen for the experimental arm ... 42
5.3.2 Treatment regimen for the control arm ... 42
5.3.3 Plasma exchanges ... 43
5.3.4 Intravenous immunoglobulin ... 43
5.3.5 Steroids ... 43
5.3.6 Anti-infective prophylaxis ... 43
5.4. Adjustment and optimization of maintenance immunosuppressive therapy ... 44
5.5. Investigational medicinal product management ... 44
5.6. Accountability procedures for the investigational medicinal product(s) ... 45
5.7. Medications and treatments that are permitted and prohibited under the protocol, including rescue medications. ... 45
5.8. Patient card ... 45
6. EFFICACY ASSESSMENT ... 45
6.1. Primary efficacy endpoints ... 45
6.2. Secondary efficacy endpoints ... 47
7. 6. SAFETY AND TOLERABILITY EVALUATION ... 47
7.1. 6.1. Description of safety evaluation parameters ... 47
7.2. Planned methods and schedule for the assessment, collection and analysis of safety endpoints ... 48
7.3. Procedures implemented for the recording and reporting of adverse events ... 48
7.3.1 Definitions ... 48
7.3.2. Severity grade ... 49
7.3.3. Role of the Investigator ... 49
7.3.4. Sponsor responsibilities ... 53
7.3.5. In utero exposure ... 54
7.3.6. Exposure during breastfeeding ... 54
7.3.7. Independent data monitoring committee ... 54
7.4. Criteria for permanent or temporary discontinuation of bortezomib ... 56
7.4.1. Temporary treatment discontinuation ... 56
7.4.2. Permanent treatment discontinuation ... 56
7.4.3. Collection methods and schedule for these data ... 56
7.4.4. Expected adverse reactions associated with bortezomib ... 56
7.4.5. Management of worsening kidney function in the control group ... 56
7.5.1. Criteria for discontinuation of plasma exchange ... 57
7.5.2. Criteria for discontinuation of intravenous immunoglobulins ... 57
7.6.1. Criteria for discontinuation of study participation ... 57
7.6.2. Follow-up procedures after study withdrawal ... 57
7.7. Criteria for early termination of part or all of the study ... 58
8. DATA MANAGEMENT AND STATISTICAL CONSIDERATIONS ... 58
8.1. Sample size ... 58
8.2. Statistical analysis strategy ... 59
8.2.1. Analysis of the primary endpoint ... 59
8.2.2. Analysis of the secondary endpoints ... 59
8.3. Data management ... 59

9. RIGHT OF ACCESS TO DATA AND SOURCE DOCUMENTS ... 59
9.1. Case report form ... 59
9.2. Identification of data considered to be source data ... 60
9.3. Right of access to data and source documents ... 60
9.4. Data processing and retention of documents and data ... 60
10. QUALITY CONTROL AND QUALITY ASSURANCE ... 61
10.1. Monitoring procedures ... 61
10.2. Quality control by an audit team ... 61
10.3. Quality control by health authorities ... 61
11. LEGAL AND ETHICAL CONSIDERATIONS ... 62
11.1. Application for authorization from ANSM ... 62
11.2. Application for an opinion from the research ethics committee ... 62
11.3. Procedures relating to data protection and information technology regulations ... 62
11.4. Amendments ... 63
11.5. Information sheet and informed consent ... 63
11.6. Final research report and conclusions ... 63
12. INSURANCE AND SCIENTIFIC COMMITMENT ... 63
12.1. Insurance ... 63
12.2. Scientific commitment ... 64
13. PUBLICATION RULES ... 64
14. LIST OF APPENDICES ... 65
APPENDIX 1: Bibliography ... 66
APPENDIX 2: Number of participating sites (investigational centers, laboratories) ... 69
APPENDIX 3: Banff 2013 classification and c4d staining scores (Haas 2013) ... 72
APPENDIX 4: Bortezomib (Velcade®) SmPC ... 73
APPENDIX 5: Bortezomib-related neurotoxicity grades ... 73
APPENDIX 6: Other non-experimental treatments used during the study ... 74
TACROLIMUS ... 75
CELLCEPT (Mycophenolate Mofetil) ... 75
CORTANCYL (prednisolone) ... 76
MYFORTIC (mycophenolic acid) ... 77
DEXAMETHASONE ... 78
APPENDIX 7: SAE Reporting Form ... 79
APPENDIX 9: Ancillary tests ... 81

LIST OF ABBREVIATIONS

**ABO**	A, B and O blood groups
**AMR**	Antibody-mediated rejection
**ANSM**	Agence Nationale de Sécurité du Médicament et des Produits de Santé (*French National Agency for Medicines and Health Products Safety*)
**APHP**	Assistance Publique et Hôpitaux de Paris
**CRID**	Clinical Research and Innovation Department
**DSA**	Donor-specific antibody
**DXMT**	Dexamethasone
**GFR**	Glomerular filtration rate
**HCG**	Human chorionic gonadotropin
**HIV**	Human immunodeficiency virus
**HLA**	Human leukocyte antigen
**IDMC**	Independent Data Monitoring Committee
**IVIg**	Intravenous immunoglobulin
**KT**	Kidney Transplantation
**MDRD**	Modification of the Diet in Renal Disease (Levvey, 2000)
**MFI**	Mean Fluorescence Intensity
**REC**	Research Ethics Committee (*Comité pour la Protection des Personnes*)
**SmPC**	Summary of Product Characteristics

**Title**	**Bortezomib for the treatment of chronic active antibody-mediated rejection in kidney transplantation - TRIBUTE**
**Project Code**	**P120119**
**EudraCT**	**2013-002322-23**
**Sponsor**	AP-HP
**Coordinating Investigator**:	Dr. Renaud Snanoudj
**Study objectives**	**Primary objective:**<br>To evaluate, at one year, the efficacy and safety of bortezomib, in combination with steroids, plasmapheresis, and intravenous immunoglobulin (IVIg), on donor-specific anti-HLA antibody levels and on the progression of chronic active antibody-mediated rejection (AMR) lesions observed on kidney graft biopsy, in patients who underwent kidney transplantation more than 3 months previously.<br>**Secondary objectives:**<br>• To assess the effect of this one-year strategy on kidney graft function, graft survival, and patient survival<br>• To evaluate the safety of bortezomib in combination with steroids, plasma exchange, and IVIg in kidney transplant patients in terms of adverse events and infectious complications<br>• To assess the kinetics of DSA titers under bortezomib and at a later stage after the injections.
**Selection criteria (inclusion and exclusion)**	**Inclusion criteria:**<br>1- Patients over the age of 18 who have received a first or second ABO-compatible kidney transplant more than three months ago, from a deceased donor (whether or not the donor's heart was still beating) or from a living donor,<br>2- Patients with one or more donor-specific antibodies (DSA) that appeared de novo, i.e. that were absent on the day of transplantation and in pre-transplantation serum samples.<br>3- Patients with findings consistent with moderate chronic active antibody-mediated rejection (g+ptc > 2) on a graft biopsy performed as part of routine follow-up or for another reason:<br>• the occurrence of de novo DSA (threshold varies by laboratory)<br>• and/or impaired kidney function<br>• and/or proteinuria of more than 0.5 g/24 hours,<br>4- Patients who have been informed and have signed a written informed consent form<br>5- Given the teratogenic risks described in the SmPC for Velcade® and CellCept®:<br>- Women of childbearing potential must have a negative pregnancy test on the day of inclusion. In addition, they must use at least one effective method of contraception before the start of treatment, during treatment, and for the entire duration of the study.<br>- Sexually active men must use condoms

	during treatment and for at least 90 days after the last administration of an investigational medicinal product during the trial. In addition, according to the CellCept® SmPC, male patients or their female partners (rather than both partners) are recommended to use an effective method of contraception during treatment and for 90 days after the male partner's last dose of mycophenolate.<br>[Update and revision of the recommendations on contraception during treatment with CellCept® or Myfortic® (ANSM references: AL20180650/AL20180651)]<br>6- Patients who are registered with or covered by a social security scheme<br>7- Patients with cellular rejection lesions associated with chronic active antibody-mediated rejection lesions may be included in the study. This rejection can be treated with three 500-mg bolus doses of methylprednisolone prior to inclusion.<br>**Exclusion criteria:**<br>1- Patients with a donor-specific antibody (DSA) present before transplantation or on the day of transplantation, or with a positive historical cross-match or a positive cross-match on the day of transplantation.<br>2- Patients receiving a third or fourth kidney transplant<br>3- Patients who have received a combined transplant with another non-kidney organ<br>4- Patients who have experienced an episode of acute antibody-mediated rejection during the current transplant<br>5- Patients with advanced graft dysfunction, defined as an estimated glomerular filtration rate (eGFR), calculated using the MDRD formula, of less than 20 ml/min/1.73 m²<br>6- Patients with histological lesions of antibody-mediated rejection that are too advanced, defined by a cg glomerulopathy score of 3/3 (Haas, 2013)<br>7- Patients with severe neuropathy, thrombocytopenia of less than 100,000/mm³, neutropenia of less than 1,000/mm³ and/or an active, uncontrolled infection<br>8- Patients with chronic active hepatitis B (positive for HBs antigen and/or HBV DNA), chronic hepatitis C, and/or a known HIV infection<br>9- Patients with hypersensitivity to bortezomib, boron, or any of the excipients<br>10- Patients with severe liver failure or abnormal liver function tests (conjugated bilirubin levels more than three times the normal range, transaminase levels more than three times the normal range), acute diffuse infiltrative pneumonia, or pericardial involvement<br>11- Patients who are deemed incapable of understanding the protocol and adhering to it<br>12- Patients who are currently participating in another treatment protocol or are within the exclusion period of another protocol.
**Study treatment**	**Bortezomib**, mannitol boronic ester for intravenous injection.<br>**Bortezomib group**: five plasma exchanges over a maximum of 10 days; two cycles of bortezomib administered three weeks apart (each consisting of four injections of 1.3 mg/m² every three days) in combination with steroids, and four courses of intravenous immunoglobulin administered

	three weeks apart (the first two courses in each of the two cycles of bortezomib), optimization of maintenance immunosuppressive therapy, and treatment to reduce proteinuria.<br>**Control group**: five plasma exchanges within a maximum of 10 days, followed by four courses of intravenous immunoglobulin, combined with steroids for the first two courses, optimization of maintenance immunosuppressive therapy, and treatment to reduce proteinuria.
**Examinations performed**	**Visits/examinations required specifically for the study:**<br>For patients in the bortezomib arm:<br>- Visits on D21, D24, D42, and D45 are specific to the study.<br><br>**For all patients:**<br>- Anti-HLA antibody assay: at inclusion<br>-- Quantitative immunoglobulin assay: at inclusion, M6, M12<br>- Lymphocyte phenotyping: W8, M6, M12<br>-- Beta HCG (women only)<br>-DNA repository: one 7 mL tube at the inclusion visit<br>- Serum repository: one 7 mL tube at the inclusion visit and D14, D35, M12<br>- Samples taken for the pharmacokinetic study (two blood samples of 5 mL each)
**Number of patients**	100 patients (50 per group)
**Research duration**	Total planned duration of the trial: 66 months<br>Inclusion period: 54 months
**Participation period for each patient**	Follow-up period: 12 months
**Number of investigational centers**	16
**Methodology**	A multicenter, randomized, open-label therapeutic trial comparing a treatment regimen combining plasma exchange, intravenous immunoglobulin, steroids, and optimization of maintenance immunosuppressive therapy, with or without two cycles of bortezomib.
**Primary endpoint and secondary endpoints**	**Primary endpoint**<br>One-year efficacy endpoint combining:<br>- a decrease of more than 50% in the titer of donor specific antibody(ies)<br>- and stabilization or improvement of the histological lesions indicative of chronic active antibody-mediated rejection observed on the graft biopsy, according to the 2013 Banff classification (glomerular capillaritis, peritubular

	capillaritis, allograft glomerulopathy).<br><br>**Secondary endpoints**<br>- Each of the primary endpoints at one year, taken separately<br>- changes in kidney function assessed by the estimated glomerular filtration rate (eGFR), using the MDRD formula<br>- Bortezomib safety (treatment-related adverse events and infectious complications)<br>- Patient survival and graft survival at 1 year
**Statistical analysis**	Analyses of the primary endpoint and all secondary endpoints will be conducted according to the intention-to-treat (ITT) principle. All statistical tests will be two-sided with a significance level of 5%.<br>The percentage of patients achieving treatment success at 1 year will be compared between the two treatment groups using the chi-square test.<br>For all secondary histological and immunological endpoints, as well as safety endpoints, comparisons of two means will be performed using a Student's t-test or, if necessary, a Wilcoxon test. Percentage comparisons will be performed using the Chi-square test or, if necessary, Fisher's exact test.

1. LITERATURE REVIEW, PRELIMINARY DATA, SCIENTIFIC RATIONALE, AND GENERAL DESCRIPTION OF THE STUDY

1.1. Definition of chronic active antibody-mediated rejection in kidney transplantation

1.1.1. Clinical circumstances of chronic active antibody-mediated rejection

Chronic active antibody-mediated rejection (AMR) refers to lesions caused by alloantibodies developed by the recipient against antigens carried by the graft, most commonly from the HLA system. These antibodies are known as donor-specific antibodies (or DSA). Chronic active AMR occurs after the first few months following transplantation, in contrast to acute AMR, which occurs within the first few weeks or even the month after transplantation. A diagnosis of chronic active AMR can be made in two circumstances:

1) progression to chronicity of acute antibody-mediated rejection in an immunized patient, meaning a patient who, prior to the current transplant, developed anti-HLA antibodies following an immunizing event (transplant, pregnancy, transfusion).

2) some time after transplantation due to the development of so-called “de novo” DSA, meaning that they did not exist prior to transplantation. These antibodies are most often class II anti-HLA antibodies. The circumstances leading to the diagnosis of chronic active AMR may include a gradual decline in kidney function, the onset of proteinuria, or, in the absence of laboratory evidence of kidney damage, a routine graft biopsy performed following the detection of de novo DSA. This study is focused on this type of antibody.

1.1.2. Diagnostic criteria for chronic antibody-mediated rejection

1.1.2.1 <u>The Banff classification (Appendix 3)</u>

The negative impact of AMR and chronic active AMR had been underestimated because valid diagnostic criteria were only recently identified (Solez, 2007). For both forms of rejection, these criteria include: - specific histological lesions on the graft biopsy,

- an immunohistochemical marker of complement classical pathway activation by DSA, C4d,
- and the detection of circulating DSAs in the patient’s serum using sensitive methods. Table 1 summarizes the chronic active AMR criteria according to the latest Banff classification (Haas, 2013).

Table 1: **Criteria for chronic active antibody-mediated rejection according to the latest 2013 Banff classification**

Histological criteria
- Duplication of the glomerular basement membrane (designated cg1 to cg3 and commonly referred to as allograft glomerulopathy)
- Multilayering of the basement membrane of peritubular capillaries*
- Arterial intimal fibrosis without elastosis
- Interstitial fibrosis with tubular atrophy
Diffuse C4d staining along the peritubular capillaries
Presence of circulating donor-specific antibodies

**Criteria requiring electron microscopy analysis*

One of the diagnostic challenges that persists despite the establishment of these criteria is that these lesions are not always present simultaneously at any given time. It has been shown that, in cases of allograft glomerulopathy, the detection of circulating DSA and C4d staining were positive in only 70% and 32% of cases, respectively (Sis, 2007). Our group demonstrated that C4d staining varied over time in serial biopsies collected according to protocol from patients with DSA (Loupy, 2011). Furthermore, during chronic rejection, the effect of anti-HLA antibodies may be independent of complement activation, occurring through a direct effect on endothelial cells or through the recruitment of cytotoxic cells bearing an Fc receptor for immunoglobulins (FcγRIII) (Colvin, 2010). When C4d staining or DSA testing is negative in the presence of histological signs of chronic active AMR, the condition is classified as “suspected” chronic antibody-mediated rejection.

1.1.2.2 Extension of the criteria for chronic antibody-mediated rejection to include microcirculatory abnormalities

It should be noted that these histological lesions are frequently associated with mononuclear cell infiltration in the glomerular and peritubular capillaries. These “capillaritis” lesions are very frequently observed in acute antibody-mediated rejection and are graded from g0 to g3 and from ptc0 to ptc3, respectively.

The Edmonton team suggested extending the diagnosis of chronic active AMR to patients with microcirculatory abnormalities, in the presence of DSA, regardless of C4d staining. These abnormalities include capillaritis lesions (glomerulitis score g > 1 and/or peritubular capillaritis score ptc > 1) and/or histological lesions of chronic rejection in the strict sense (glomerulopathy score cg > 1 and/or peritubular basement membrane multilamination score > 2) (Einecke, 2009). Like chronic active AMR, this clinical picture is strongly associated with late graft loss (see below).

1.1.3. Incidence and adverse prognostic significance of chronic active AMR and so-called “de novo” DSA in kidney transplantation

1.1.3.1 Incidence and prognosis of chronic active AMR

The incidence of chronic active AMR has been determined based on these diagnostic criteria. In a series of 771 kidney graft biopsies performed due to dysfunction, chronic active AMR was diagnosed in 9.3% of cases, representing 66 patients. Incidence, which is rare before one year, gradually increases over time. In this group, chronic active AMR was associated with a very marked decrease in graft survival from the time of diagnosis (54% at one year and 8% at five years) (Colvin, 2010).

In a study by the Edmonton group (Einecke, 2009), nearly 15% of biopsies performed one year later due to functional decline met the full diagnostic criteria for chronic active AMR, according to the Banff classification. This group also found an association between late graft loss and microcirculatory abnormalities in a broad sense, with C4d staining being less important. When the diagnosis of chronic active AMR was broadened to include microcirculatory abnormalities, it accounted for 63% of graft losses that occurred in patients who underwent a graft biopsy after one year (Einecke, 2009).

In the U.S. multicenter DeKAF study, 173 patients underwent a biopsy due to functional decline after an average of 7.3 years. In total, 57% of these patients had positive C4d staining and/or positive DSA results. Graft survival based on biopsy results was significantly lower in the presence of C4d staining positivity, regardless of whether circulating DSA were present (Gaston, 2010). However, survival rates are even lower in the presence of DSA (relative risk of graft loss: 4.54 for C4d+ DSA+/3.75 for C4d+ DSA-/

2.17 for C4d- DSA+, compared to the control group of C4d- DSA-).

In a study of 153 graft losses not related to patient death, allograft glomerulopathy accounted for 15% of graft losses

after an average follow-up of 50 months; incidence rose to 20% for graft losses occurring between one and five years after transplantation (El-Zoghby, 2009).

1.1.3.2 Incidence and prognostic value of donor-specific antibodies

Antibody detection technique

Several studies have assessed the frequency and prognosis of DSA in kidney transplant patients. This problem was significantly underestimated for many years, due to the lack of an effective detection method. For a very long time, the only method for detecting anti-HLA antibodies was the microlymphocytotoxicity assay. This technique involves reacting the patient's serum with a panel of phenotyped lymphocytes expressing known HLA antigens, to test the ability of the antibodies present in the patient's serum to activate rabbit complement and induce complement-dependent cytotoxicity against target cells. This test assessed a patient's immunity against a panel of donors representative of the geographic region. The result was reported as a percentage of reactivity relative to this panel. The use of complement meant that only antibodies capable of activating complement, and present above a minimal threshold, could be detected, resulting in low sensitivity for this test.

More sensitive detection and identification techniques, known as solid-phase techniques (ELISA, Luminex), have recently been developed. They have three characteristics that distinguish them from microlymphocytotoxicity:

1) They are not based on the antibody's ability to activate complement, but on its ability to bind to its antigenic target
2) They make it possible to test for the presence of antibodies against a single HLA antigen at a time, which allows for a precise determination of the antibodies present in a recipient
3) They allow for a quantitative classification of the antibodies present

The Luminex technique is currently the most widely used method for detecting anti-HLA antibodies. First, serum is screened for anti-class I and anti-class II antibodies. If this screening test is positive, the specific antigens recognized by the present antibodies are identified one by one (a technique known as the "single antigen" method). This is a flow cytometry technique in which the patient's serum is brought into contact with microspheres. Each microsphere carries a specific HLA antigen and has its own fluorescence level, which allows it to be identified during flow cytometry analysis. When the serum contains an antibody specific to an antigen, a double fluorescent signal is detected: one from the sphere and one from the fluorescent anti-human antibody, signaling that the antibody has bound to the specific sphere. This technique makes it possible to easily determine whether, at a given time point (t), the patient carries antibodies directed against HLA specificities expressed by the graft (DSA). It has high sensitivity, as it detects very low mean fluorescence intensity (MFI) levels corresponding to very low antibody titers, well below the detection threshold of lymphocytotoxicity assays. As a result, it is now possible to monitor specific antibodies both qualitatively and quantitatively. With high-quality serological monitoring, it is thus possible to determine whether an antibody was present prior to transplantation or appeared "de novo" after transplantation, and to track its titer over time and in relation to the treatments received.

Frequency and prognostic value of de novo anti-HLA antibodies

Gill et al. prospectively monitored the emergence of de novo anti-HLA antibodies in 70 non-immunized patients during the first year after transplantation: 16% of patients developed de novo anti-HLA antibodies, including DSA in three-quarters of cases (Gill, 2010).

In another study, 54 patients were followed longitudinally for an average of 40 months: 20% developed de novo anti-HLA antibodies, including DSA in 45% of cases (Mao, 2007). The negative prognostic role of these de novo antibodies has been particularly well demonstrated. Six of the 11 patients who developed de novo anti-HLA

antibodies suffered graft failure. Of the five patients who developed de novo DSA, four lost their grafts after an average of two years following the development of antibodies and one patient retained his/her graft but suffered severe dysfunction.

Between 1998 and 2003, the Nantes team conducted an annual prospective follow-up study of 1,229 patients who had undergone transplants between 1972 and 2003. The incidence of the three situations in this study was as follows: absence of antibodies 83.1%, DSA 5.5%, and non-donor-specific antibodies 11.4% (Hourmant, 2005). The de novo nature is difficult to establish in this study. The presence of antibodies, whether donor-specific or not, was associated with poorer survival and poorer graft function.

Lachmann et al. conducted a cross-sectional study of 1,014 patients with a post-transplant follow-up period ranging from six months to 18 years (median: five years) (Lachmann, 2009). Depending on the post-transplant time period during which patients were studied, the percentage of patients with de novo non-specific and donor-specific anti-HLA antibodies varies: between two and four years, 7.4% and 4.6% respectively; between four and eight years, 17.6% and 9.5% respectively; after eight years, 12% for both types of antibodies. Graft survival in patients with de novo antibodies after transplantation is significantly lower: 61% versus 82%.

In a large, multicenter collaborative study, Terasaki and Ozawa examined graft survival in 2,231 patients, two years after screening for anti-HLA antibodies (Terasaki, 2005). When considering only patients who did or did not develop de novo antibodies after transplantation, the difference in the failure rate is highly significant: 16.7% of 233 patients with antibodies versus 6.5% of 1,331 patients without antibodies ($p=1.10^{-7}$). The difference between the two groups of patients, those with and those without antibodies, becomes more pronounced as kidney function at the time of screening deteriorates.

Another study by the Edmonton group showed that the presence of DSA during a biopsy performed due to functional decline was associated with microcirculatory abnormalities such as those described above (Hidalgo, 2009). Of the 145 patients tested, 37% had DSA, 60% of which were de novo DSA. These de novo DSA were class II antibodies, either isolated or associated with class I antibodies, but very rarely isolated anti-class I antibodies. With regard to C4d staining, it was positive in only 67% of the biopsies associated with de novo DSA. Graft survival following biopsy was similar across three groups of patients: patients without anti-HLA antibodies, patients with non-donor-specific antibodies, and patients with DSA prior to transplantation. Only the group of patients who developed de novo DSA had significantly poorer graft survival, with a three-year graft survival rate of just 47.6%.

1.2. Treatments used in the prevention and management of antibody-mediated rejection

1.2.1. Treatments for acute antibody-mediated rejection

As we will see later, there is very little data on the treatment of chronic antibody-mediated rejection. To date, AMR is the only form of antibody-mediated rejection that has been the subject of controlled and uncontrolled studies. The treatment of patients with DSA, whether for prevention or curative treatment, is based on two approaches:

1) neutralizing or eliminating pathogenic antibodies, in this case, anti-HLA antibodies, using intravenous immunoglobulins (IVIg) and plasmapheresis (or immunoadsorption)

2) inhibiting the synthesis of these antibodies, the only treatment currently specifically active on B cells being the anti-CD20 monoclonal antibody, rituximab.

Compared to plasmapheresis, which allows for the rapid removal of pathogenic antibodies, IVIg has a prolonged

effect that extends beyond the product's half-life, suggesting effects other than anti-idiotypic neutralization (Jordan, 2009).

In recent years, rituximab has been routinely added to the treatment received by patients with DSA, even though it has not been the subject of any controlled studies. This antibody is active only against naive and memory B cells, but not against plasma cells, and it has no immediate effect on circulating immunoglobulin levels. However, the mechanism of action most frequently cited to explain its effect is thought to be the elimination of alloreactive B cells, thereby preventing their differentiation into plasma cells that secrete DSA.

There are three distinct phases in the treatment of patients with anti-HLA antibodies:

1. **Desensitization prior to transplantation.** The efficacy of IVIg used prior to transplantation, whether or not in combination with rituximab, has been demonstrated in this indication in highly sensitized patients to reduce their anti-HLA antibody levels and to enable them to undergo transplantation with a negative cross-match (Vo, 2008). However, while access to transplantation has improved and short-term survival rates are satisfactory, the rate of acute rejection remains high: 50%, of which 30% are acute antibody-mediated rejections. This incidence of acute antibody-mediated rejection raises the legitimate question of the incidence of chronic humoral rejection following such a strategy. However, these data are not available.

2. **Desensitization after transplantation.** This technique has been extensively studied at Hôpital Necker (Anglicheau, 2007, Loupy, 2010). It comprises four courses of IVIg administered every three weeks, beginning on the day of transplantation in patients with preformed, donor-specific anti-HLA antibodies, to prevent acute antibody-mediated rejection. The results were satisfactory, with a one-year graft survival rate of 95% and a 10% incidence of AMR. However, a routine biopsy conducted after one year revealed glomerulitis lesions in 60% of cases and allograft glomerulopathy lesions in 28% of cases – lesions consistent with CHR (Anglicheau, 2007). In a second phase, this prophylactic treatment was supplemented by adding plasma exchanges at the start of transplantation and rituximab to the IVIg courses (Loupy, 2010). This strategy led to a significant reduction in chronic and subclinical antibody-mediated rejection lesions on routine biopsies, as well as a decrease in anti-class II DSA titer.

3. **Curative treatment for acute antibody-mediated rejection.** Lastly, as we have seen, these preventive measures do not eliminate the risk of antibody-mediated rejection. To date, there are no published controlled trials on curative treatment for AMR. However, several single-center studies have reported that plasma exchange is quite effective, as it allows for faster elimination of pathogenic antibodies than IVIg, when used in combination with rituximab. Compared with IVIg, only this combined strategy results in a significant improvement in graft survival following rejection (92% versus 50% at three years) and a reduction in DSA titer (Lefaucheur, 2009).

1.2.2. Treatment for chronic antibody-mediated rejection

There is no standard treatment for chronic active AMR. To date, only three studies have been published on the use of rituximab. The first (Billing, 2008) reported on the treatment of six children who had undergone kidney transplantation and were presenting with chronic active AMR, using four weekly courses of IVIg, followed by a rituximab injection. All of the children had microvascular lesions (peritubular capillaritis, with three of them also having allograft glomerulopathy) and positive C4d staining; four of them had de novo DSA. Kidney function improved or stabilized in four of the six children, while two children, who had the most advanced glomerulopathy lesions (cg3), did not respond to treatment. DSA became undetectable in just one of the four children, but the technique used (ELISA) does not allow for a precise determination of changes in antibody titer.

The second study (Fehr, 2009) reported on four patients with late-onset, progressive graft dysfunction, with circulating DSA, few histological signs of antibody-mediated rejection, and, in three out of four cases, signs of cellular rejection and diffuse C4d staining. All patients received bolus doses of Solumedrol and a course of rituximab; three of them received a course of IVIg. Kidney function improved at six months in all patients, but at one year, one patient experienced a new episode of acute rejection. DSA titer decreased in two patients. There was no control histology. The data from this initial study is therefore limited and one might wonder whether the beneficial effect on kidney function was related to the steroids used to treat rejections, all of which had a cellular component.

In the third study (Rostaing, 2009), 14 kidney transplant patients, including 7 patients with chronic hepatitis C, were treated with rituximab for allograft glomerulopathy, as indicated by a graft biopsy performed due to impaired kidney function and severe proteinuria. DSA were present in six of these patients (43%). This subgroup probably corresponds most closely to the definition of chronic active AMR, as hepatitis C can produce an appearance resembling allograft glomerulopathy. Of these six patients, five lost their graft despite treatment with rituximab, compared with only two of the eight patients without DSA.

In conclusion, we can therefore say that the use of rituximab ± IVIg may be effective in certain cases of chronic active AMR. However, the evidence regarding DSA titer and histology, two essential prognostic factors, is insufficient. Furthermore, no controlled studies are currently available for this indication.

1.2.3. Failure of current antibody-targeted therapies: evidence for the role of plasma cells in anti-HLA antibody production?

While significant progress has been made in the prevention and treatment of AMR, making transplantation possible for patients previously considered very difficult transplant candidates, it is clear that AMR and chronic active AMR remain significant threats to graft survival in the medium and long term. One of the most likely reasons is that current combinations of immunosuppressants, including rituximab, have little effect on memory B cells and no effect at all on plasma cells in secondary lymphoid organs and bone marrow (Ramos, 2007).

The respective roles of plasma cells and memory B cells at different time points during transplantation (before, after, and during an episode of acute rejection) remain poorly understood. The evidence supporting the role of these two cell types comes from two studies that successfully reproduced, in vitro, the synthesis of anti-HLA antibodies with the same specificity as those found in serum, using circulating memory B cells (Han, 2009, Zacchary, 2007) or medullary plasma cells (Perry, 2008), collected from immunized patients who had been stimulated in a polyclonal manner.

These studies do not allow us to conclude which cell type is involved in antibody synthesis in vivo or at what point in time relative to transplantation. It is possible that two different cell types are involved: in the absence of the antigen (before transplantation) or in the presence of the antigen (after transplantation). Based on parallels with humoral responses to other protein antigens, the following hypotheses can be formulated (Snanoudj, 2010):

- during the primary humoral response (which occurs during pregnancy, a blood transfusion, or at the time of transplantation), naive B cells are activated upon contact with the alloantigen and undergo maturation, hypermutation, and isotype switching. They will differentiate, with some becoming memory B cells and others becoming medullary plasma cells.
- during the secondary humoral response, such as following transplantation in immunized patients, memory B cells become active and differentiate into short-lived plasma cells that synthesize large quantities of anti-HLA antibodies.
- it is likely that, outside these “acute” periods, the synthesis of anti-HLA antibodies at a stable level is due to long-lived plasma cells. This likely corresponds to the pre-transplantation period, when antibody levels fluctuate slightly and slowly in the absence of antigen, but also to the post-transplantation period, when alloantibody production becomes “chronic” following an acute phase.

We will see further on that bortezomib, chosen for its effect on plasma cells, likely has varying efficacy, depending on the different stages of alloantibody synthesis. Plasma cells are, in fact, most sensitive to its pro-apoptotic action when they are at the peak of their protein synthesis, likely at the onset of the primary or secondary response, as short-lived plasma cells, before long-lived plasma cells take over.

In any case, it is precisely because they target plasma cells that proteasome inhibitors have been proposed as a treatment for antibody-mediated rejection.

1.3. Rationale for using a bortezomib-based strategy for the treatment of chronic active AMR

1.3.1. Description and role of the ubiquitin-proteasome system

Cellular proteins have a short lifespan and are degraded by two different mechanisms: the lysosomal system (10–20% of proteins) and the proteasome system (nearly 80% of intracellular proteins). The proteasome carries out five types of proteasic enzymatic activity. Before being degraded by the proteasome, proteins are first tagged with ubiquitin molecules by ubiquitinating enzymes, which facilitate both the intracytoplasmic transport of these proteins and their subsequent degradation. Potentially, any protein involved in cell signaling is subject to regulation of its effects through ubiquitination and proteasome-mediated degradation. The ubiquitin-proteasome system thus plays an important role in numerous cellular processes, such as protein turnover and quality control, the cell cycle and apoptosis, transcription and cell signaling, the immune response (particularly antigen presentation via MHC class I), inflammation, and development (Everly, 2009).

The proteasome also plays a role in the endoplasmic reticulum (ER) stress response. The factors that disrupt the proper functioning of the ER lead to the accumulation of unfolded proteins within it, a process that results in a phenomenon known as ER “stress”. The detection of these proteins triggers a process known as the “unfolded protein response” (UPR), which restores proper ER function. In the event of prolonged stress, a terminal UPR response is initiated, leading to cell apoptosis, either via the mitochondrial pathway or the caspase pathway. Proteasome inhibition leads to the accumulation of unfolded proteins in the ER, particularly in cells undergoing constant protein synthesis, such as plasma cells during certain phases, and thus to caspase-dependent apoptosis.

1.3.2. Proteasome inhibition and immune response

Proteasome inhibition by bortezomib induces apoptosis in both polyclonally stimulated and unstimulated B cells in vitro and ex vivo. However, polyclonal B-cell activation and differentiation into plasma cells significantly increase sensitivity to the pro-apoptotic effect of bortezomib (Cascio, 2008). Bortezomib significantly reduces the primary IgM-specific response in vivo, with a suppressive effect that persists long after injection in mice.

In addition to antigen presentation and the humoral response, proteasome inhibition also has an immunosuppressive and pro-apoptotic effect on CD4+ T-cells activated by allogeneic dendritic cells, making it a potentially preferred immunosuppressant.

1.3.3. The use of bortezomib as a proteasome inhibitor – Use in multiple myeloma and AL amyloidosis in combination with dexamethasone

Bortezomib is a reversible and highly selective proteasome inhibitor. Bortezomib dissociates rapidly from proteasome; consequently, proteasome inhibition by bortezomib is rapidly reversible.

Proteasome inhibition leads to cell cycle arrest and cell apoptosis. Bortezomib is cytotoxic to various types of cancer cells, which are more sensitive to the pro-apoptotic effects of proteasome inhibition than normal cells. Bortezomib reduces tumor growth in vivo in numerous pre-clinical tumor models, including multiple myeloma.

Bortezomib and the combination of bortezomib and dexamethasone in the treatment of myeloma

Bortezomib (Velcade®) is indicated as monotherapy for the treatment of progressive multiple myeloma in patients who have received at least one prior treatment and who have either already undergone a bone marrow transplant or are ineligible for one. In a Phase II study and a Phase III study comparing bortezomib with dexamethasone (DXMT), previously treated patients with relapsed or refractory multiple myeloma experienced an increased time to progression (TTP). The DXMT arm was discontinued and all patients were subsequently treated with bortezomib. Patients who had not responded satisfactorily to bortezomib were given DXMT as an additional treatment, which led to an improvement in response in 18% of patients. Another study showed that the addition of DXMT in patients who had not responded satisfactorily to bortezomib led to an improved response in two-thirds of cases (Jagannath, 2006, Rajkumar, 2011).

The addition of bortezomib to the melphalan-prednisone combination represents a new treatment option for first-line multiple myeloma in patients over the age of 65 or for whom high-dose chemotherapy is contraindicated: this triple combination significantly improves patient survival and time to progression (TTP) (Rajkumar, 2011).

Bortezomib and the combination of bortezomib and dexamethasone in the treatment of primary AL amyloidosis

In primary AL amyloidosis, the production of an amyloid light chain is associated with plasma cell dyscrasia. In most cases, the myelogram does not show any medullary tumor-related plasma cell proliferation. The combination of bortezomib and DXMT, as in multiple myeloma, shows an excellent hematologic response rate. DXMT and bortezomib have a synergistic pro-apoptotic effect in vitro and in vivo on dysplastic plasma cells, making their combination a promising treatment option for multiple myeloma (Jagannath, 2006) and AL amyloidosis (Kastritis, 2007). A similar synergistic effect can be envisioned in vitro on non-dysplastic plasma cells.

1.3.4. Pre-clinical data on the use of bortezomib in transplantation

The proteasome was first tested in animal models of autoimmune diseases: post-streptococcal polyarthritis, autoimmune encephalitis, and lupus. In this last model, bortezomib eliminated both short-lived and long-lived plasma

cells. Furthermore, it directly eliminated the plasma cells responsible for the synthesis of anti-double-stranded DNA antibodies and reduced the severity of lupus glomerulonephritis (Neubert, 2008). It is important to note that proteasome inhibition in these studies selectively induces a reduction in autoantibodies compared with polyvalent immunoglobulins. Bortezomib has also been tested in several animal models of allogeneic transplantation. It has been shown that bortezomib inhibits mixed T-lymphocyte cultures and cytotoxic T-cell activity in a dose-dependent manner. Furthermore, it significantly increased the survival of Langerhans islet allografts (Wu, 2004) and heart allografts (Luo, 2001) in mice. In an antibody-mediated rejection model, Ashton-Chess and colleagues in Nantes tested bortezomib in a rat cardiac allograft model. To prevent acute antibody-mediated rejection, bortezomib administered early after transplantation (from D0 to D20) prolongs graft survival in a dose-dependent manner, with a reduction in circulating donor-specific antibody titers (Ashton-Chess, 2010). Interestingly, however, when injected well after transplantation (D60 to D100), it induces the same effects on graft survival and the reduction in DSA titer, as well as a decrease in histological lesions and C4d deposits in the graft. These results strongly suggest that bortezomib has a beneficial effect on chronic antibody-mediated rejection in this model.

1.3.5. Clinical data on the use of bortezomib in kidney transplant patients

Table 2: **Published studies on the use of bortezomib in kidney transplantation**

Author	No.	Patients	Results
**Everly, 2008**	6	• Late-onset acute cellular rejection (1 month–7 years) with DSA, corticosteroid-resistant	• Recovery from acute rejection<br>• Reduction in iDSA titer >50%
**Walsh, 2010**	2	• Only one confirmed case of early acute antibody-mediated rejection (D14)<br>• Treatment with steroids/PP/RTX	• Recovery from acute antibody-mediated rejection<br>• Resolution of DSA in two weeks
**Trivedi, 2009**	11	• Clonal stimulation/deletion, pre-transplant<br>• PE+bortezomib at DSA emergence	• Sustained reduction in DSA at four months in six of eight patients
**Sberro-Soussan, 2010**	4	• Subacute antibody-mediated rejection between one and three years post-transplantation, with a persistent DSA titer	• No effect on iDSA
**Flechner, 2010**	20	• Difficult: 20 months post-RT, de novo DSA, C4D+<br>• No histological findings of AMR, but one of three patients previously treated for AMR	• 55% reduction in iDSA, but rebound in 60%<br>• Improvement in kidney function
**Walsh, 2011**	28	• 12 cases of early antibody-mediated rejection<br>• 16 cases of delayed antibody-mediated	• % of patients with a reduction in iDSA >50%: 76.9% (early) versus 35.3% (late)
**Waiser, 2011**	19	• 19 cases of acute or chronic antibody-mediated rejection<br>• 10 treated with bortezomib/nine with 500 mg RTX	• 60% graft survival in the bortezomib group, versus 11% in the RTX group, 18 months after treatment

***iDSA**: immunodominant donor-specific antibodies (those with the highest titer);*
***AMR:** antibody-mediated rejection; KT: kidney transplantation; **PE**: plasma exchanges; **RTX**: rituximab*

Clinical data on the use of bortezomib in kidney transplantation is currently limited. The first study involved the treatment of episodes of late acute cellular rejection associated with the development of DSA in six patients who were refractory to standard anti-rejection therapy (ATG steroids) and, sometimes combined with antibody-targeted therapy (rituximab, plasmapheresis) (Everly, 2008). Bortezomib resulted in the resolution of acute rejection and a reduction in DSA titer in all patients (by an average of 50%, with the nadir reached on average 18 days after the bortezomib cycle) during a six-month follow-up period. These results, obtained in a specific clinical form of rejection, may be related to the combined anti-T and anti-B effects of bortezomib.

The same team reported the effective treatment of two episodes of AMR with bortezomib, but in combination with steroids, plasmapheresis, and rituximab, which makes it difficult to analyze the respective roles of each immunosuppressant (Walsh, 2010). However, one of the two patients experienced a rebound in DSA titer two months after the initial treatment; this patient responded to a second course of bortezomib.

The Mayo Clinic team conducted in vitro tests to assess the effect of several immunosuppressants on the synthesis of anti-HLA antibodies by medullary plasma cells collected during transplantation in immunized patients (Perry, 2009). While ATG and rituximab had no effect, bortezomib significantly inhibited the allospecific memory B-cell response. This effect was associated with activation of the caspase pathway, compared with plasma cells not treated with bortezomib, demonstrating the pro-apoptotic mechanism of action. In vivo, two immunized patients who were DSA-positive on the day of transplantation experienced an episode of acute antibody-mediated rejection that was treated with bortezomib. This treatment resulted in clinical and histological resolution of acute antibody-mediated rejection (assessed at one year by means of a screening biopsy), a decrease in the number of anti-HLA class I and II antibodies, and a significant reduction in DSA titer. In addition, both patients underwent bone marrow aspiration to isolate medullary plasma cells at the time of rejection and one week after bortezomib treatment. Bortezomib caused a reduction in the number of

medullary plasma cells, as well as a marked reduction in the number of anti-HLA antibodies synthesized in vitro and in their titer. This mechanistic study of two patients therefore strongly suggests that bortezomib is effective against medullary plasma cells that synthesize anti-HLA alloantibodies.

Waiser et al. compared the efficacy of a single cycle of bortezomib in 10 patients with that of a historical cohort of nine patients treated with a single dose of 500 mg of rituximab for the treatment of antibody-mediated rejection (Waiser, 2011). Patients in both groups also received plasma exchanges and IVIg. It should be noted that three of the rejections in each group were chronic antibody-mediated rejections, the others being acute rejections. At 18 months, graft survival was 60% in the bortezomib group, compared with 11% in the rituximab group (p=0.07). Kidney function was significantly better in the bortezomib group: 2.5 ± 0.6 mg/dL versus 5.1 ± 2.1 mg/dL, p=0.008. Survival following a single cycle of bortezomib in the short term after the episode of rejection remains inadequate, even though it is higher than that of the rituximab group, which was used here at a very low dose. Interestingly, all DSA were de novo.

Trivedi et al. reported their experience of using bortezomib in 11 patients at the time of the emergence of anti-HLA antibodies following transplantation – a time window that is of particular therapeutic interest, since, as we will see below, this is perhaps the most promising period, given that plasma cells are in a phase of peak protein synthesis (Trivedi, 2009). The associated immunosuppressive protocol was quite unusual, which limits the scope of the study; it followed a clonal stimulation/deletion protocol (infusion of donor hematopoietic stem cells and leukocytes, cyclophosphamide, lymphoid irradiation, anti-thymocyte globulin, rituximab), followed by maintenance treatment consisting solely of steroids. Bortezomib, in combination with plasma exchange, was administered to patients who were prospectively monitored for the onset of DSA, at the time of its onset and thus before the development of antibody-mediated rejection lesions. This treatment resulted in a substantial reduction in DSA titer in all patients. Two patients experienced a rebound in DSA titer during the four-month follow-up period and therefore received another injection of bortezomib.

This finding should be compared with the results obtained in four patients at Hôpital Necker (Sberro-Soussan, 2010). These four patients, who were immunized prior to transplantation and tested positive for DSA on D0, were treated with a course of bortezomib alone some time after transplantation (between one and three years), due to the persistence of a high but stable DSA titer and persistent antibody-mediated rejection lesions on graft biopsy. Bortezomib has been shown to be ineffective in this indication for reducing DSA titer. This difference in efficacy compared to the previous study, conducted during de novo antibody synthesis or during an episode of acute rejection, suggests that plasma cells are most sensitive to proteasome inhibition during the peak phase of protein (and thus immunoglobulin) synthesis. This is consistent with the aforementioned in vitro data: it is when the plasma cell's protein machinery is most active that it is most susceptible to bortezomib-induced apoptosis. Both situations, de novo antibody synthesis and acute antibody-mediated rejection, likely involve short-lived plasma cells that are called upon to synthesize immunoglobulins intensively, as part of a primary humoral response and a secondary response, respectively.

Several studies support this hypothesis:

1) A recent conference presentation on the use of bortezomib in 11 kidney transplant recipients with AMR and different types of anti-HLA antibodies: pre-existing DSA, de novo DSA, and non-donor-specific anti-HLA antibodies (Walsh, ATC 2010). De novo DSA were the most sensitive to bortezomib compared with the other two types of antibodies, reinforcing the hypothesis that plasma cells are most sensitive to bortezomib during rising DSA titer. The efficacy of bortezomib in treating de novo DSA has also been suggested very recently.

2) Flechner et al. treated 20 patients with acute antibody-mediated rejection, diagnosed on a biopsy performed for graft dysfunction, sometimes occurring late (average time: 20 months [range: 1–71 months]), related to de novo DSA, using a combination of steroids, plasmapheresis, and bortezomib (one cycle). Six of these patients had already been treated with plasmapheresis, IVIg, and steroids. The reduction in dominant DSA titer was 55 ± 22%, but 10

patients experienced a rebound in titer after reaching the nadir. The response was better before the onset of severe dysfunction and/or proteinuria (Flechner, 2010).

3) The efficacy of bortezomib was compared in 12 cases of early acute rejection (time since transplantation of 2.2 ± 2.0 months) and 16 late rejections (55.3 ± 48.5 months). It is important to note that 36% of the treated cases had been refractory to prior treatment with plasmapheresis, IVIg, rituximab, and ATG. Furthermore, the validity of these results is limited by the fact that patients were treated with one cycle of bortezomib combined with rituximab and plasmaphereses (Walsh, 2011). The percentage of patients with a reduction in DSA titer on D14 of treatment was 76.9% in the early rejection group versus 35.3% in the late rejection group, p=0.03. An improvement in kidney function was observed in both groups, with a greater improvement following early rejection; however, patients in this group had a better baseline GFR. This difference in efficacy likely reflects the much longer diagnostic delay between the onset of DSA and the appearance of histological lesions in late rejection, which develops more insidiously than in acute rejection.

All published studies on bortezomib in kidney transplantation should be interpreted with great caution, given the heterogeneity of indications, time since transplantation, and prior treatments administered in combination with bortezomib. Nevertheless, it seems clear that early initiation of treatment following the onset of rejection or antibodies is a factor that appears to influence the efficacy of bortezomib.

Another factor may explain the lack of response to bortezomib reported by Sberro-Soussan et al.: its use as a monotherapy without steroids or plasma exchange, and perhaps at an insufficient dose. Indeed, as discussed above, it has been clearly demonstrated in myeloma that steroids and bortezomib have a synergistic pro-apoptotic effect on plasma cells in vitro and in vivo. Furthermore, again by analogy with myeloma, a single cycle of bortezomib may be insufficient, as efficacy is often assessed from the second cycle in myeloma.

1.4. Description of the new strategy

The treatment we provide for kidney transplant patients with chronic rejection is innovative in two ways:

1) the therapeutic aspect, involving the first-ever use of a proteasome inhibitor, bortezomib, for this condition. To maximize its efficacy, bortezomib is incorporated into a treatment protocol that initially includes plasmapheresis combined with IVIg, corticosteroids administered with each bortezomib injection, optimization of maintenance immunosuppression using a triple-drug regimen consisting of corticosteroids, mycophenolate mofetil, and tacrolimus, and nephroprotective treatment with angiotensin II receptor blockers in patients with proteinuria. Bortezomib will be used for two cycles, three weeks apart, each consisting of four injections of 1.3 mg/m² on D1, D4, D8, and D11.

2) Early intervention: as discussed in Chapter 2.3.5, it is likely that for treatment with bortezomib to be successful, it must be administered as soon as possible after the onset of donor-specific antibodies. That is why we recommend screening for DSA every six months during the first year and annually in subsequent years. This will make it possible both to confirm the de novo nature of an antibody, which, by definition, will develop at a specific time during the transplantation process, and to treat chronic antibody-mediated rejection early, before severe lesions develop. Robert Colvin has proposed a classification of chronic antibody-mediated rejection based on four stages of progression (Colvin, 2007):

1. Detection of DSA in the bloodstream
2. Complement activation
3. Development of histological lesions in the graft (subclinical antibody-mediated rejection)
4. Clinical stage: chronic graft dysfunction

In animal models (primates) of CHR, stages 1 and 2 are reversible if effective treatment is administered. Stages 3 and 4, however, prove refractory to treatment and may persist even after DSA and C4d staining have resolved (Colvin, 2007). The majority of cases of CHR in humans are diagnosed at stage 4; in any event, de novo DSA cases are consistently diagnosed at stages 3 and 4, as routine screening for anti-HLA antibodies was not carried out in real time until recently. This likely explains why no effective treatment has been identified to date.

Participating centers conduct annual routine screening for anti-HLA antibodies using a sensitive technique, Luminex. In the event of a positive screening result, the antibodies are identified using the same Luminex “single-antigen” technique. We therefore recommend continuing this strategy for screening and identifying anti-HLA antibodies.

In the event of a de novo positive result for an antibody directed against the donor, a graft biopsy is performed to look for chronic active AMR lesions. Patients can also be identified via protocol biopsies performed one year after transplantation. If these biopsies reveal chronic active AMR lesions and a de novo DSA is identified, a diagnosis of subclinical chronic active AMR is made and the patient may be included in the study. Finally, a for-cause biopsy is performed in the event of a progressive deterioration in graft function or the onset of proteinuria. If there are histological signs of chronic active AMR, testing for DSA is performed; if the result is positive for a de novo DSA, the patient may be included in the study.

The first two recruitment methods occur earlier than the last one, which is already associated with a dysfunction; however, given the de novo nature of the antibodies, we still consider this to be an early stage. We do not plan to treat patients solely based on the presence of an antibody without histological lesions (corresponding to Colvin’s Stages 1 and 2) for two reasons:

1. The use of immunosuppressive therapy, which may cause adverse effects, merely because of a biological abnormality, even if it has a poor prognostic significance

2. The difficulty of predicting whether a DSA is pathogenic at an individual level. Some DSA, particularly class I DSA, can be detected over a long period without causing any histological damage (accommodation).

1.5. Summary of the benefits and, where applicable, the foreseeable and known risks for individuals participating in the study

1.5.1. Individual benefit

As we saw earlier, there is no standard treatment for chronic active AMR and the three published studies on rituximab therapy show only very limited results that are not conclusive. The current approach taken by transplant teams when a patient is diagnosed with chronic active AMR is to optimize maintenance immunosuppressive therapy using the maximum tolerated doses and to add renoprotective therapy. If the fibrotic lesions and glomerulopathy are not too advanced, some teams use a combination of plasmapheresis ± IVIg ± rituximab, although no study has demonstrated the benefits of this strategy. The idea is to try to counteract the effect of DSA and, ultimately, to reduce their synthesis. Our strategy at Hôpital Necker is to try treatment with plasmapheresis and IVIg, as this treatment is associated with minimal immunosuppression and low morbidity. In particular, we do not use rituximab with this indication, given the lack of proven efficacy and the risk of infection associated with the use of bortezomib in kidney transplant patients (Kamar, 2010).

- **For patients in the bortezomib arm**, the individual benefit of effective treatment for chronic active AMR would be considerable, as it would extend the lifespan of the graft and delay or even prevent the need to resume dialysis. It would thus help extend the benefits of kidney transplantation, in terms of patient survival, quality of life, and comorbidities, which have been well demonstrated in comparison with dialysis.

This survival benefit obviously cannot be directly assessed by a study with a primary endpoint at one year. However, we have clearly demonstrated that the one-year efficacy criterion combines a reduction in antibody titer and histological lesions, both of which are markers that correlate with graft survival during chronic active AMR.

- **For patients in the control group**, the benefit of being included in the study lies in the earlier diagnosis of chronic active AMR compared with the usual time to diagnosis and in the early administration of a treatment with the best theoretical benefit-to-risk ratio. Participating centers' commitment to this protocol for the early detection of de novo DSA will enable treatment with plasmapheresis and IVIg to be started at an early stage, as well as optimizing immunosuppressive therapy and providing nephroprotective treatment. It is likely that this treatment is insufficient, given the studies reporting a poorer prognosis for chronic active AMR in patients receiving only maintenance immunosuppressive therapy. However, we believe that the temporary neutralization DSAs by IVIg, together with therapeutic optimization, may slow the progression of chronic kidney disease, as has been observed in many nephropathies. To avoid penalizing patients in the control group, patients with rapid deterioration of kidney function, defined as an increase in serum creatinine of more than 20% between two tests performed three months apart, will be eligible for a graft biopsy. In the event of an acute worsening of antibody-mediated rejection lesions, treatment – the choice of which will be left to the medical team at the patient's center – may be offered to the patient.

1.5.2. Collective benefit

Given the frequency of chronic active AMR and its role in late graft loss, the development of an effective treatment will have implications for a large proportion of the population of kidney transplant patients. This will, in fact, be the first randomized trial for the treatment of chronic active AMR. It will provide a scientifically valid, actionable response for the kidney transplant patient community. If this strategy proves effective for this indication, this treatment could benefit a large proportion of kidney transplant patients, as we have shown that there is no specific high-risk group and that de novo DSA can develop in any kidney transplant patient.

Furthermore, this will also be the first controlled trial to assess the use of bortezomib in kidney transplantation. We have highlighted the considerable heterogeneity of the few retrospective studies published on the subject in chapter 2.3.5. Given the considerable cost of the drug and its potential toxicity, it seems important to establish with certainty whether its use offers any benefit in the treatment of chronic active AMR. That is why the two treatment arms differ only in the use of bortezomib.

Monitoring DSA titer at six months and one year will also provide us with a deeper understanding of the kinetics of the response to bortezomib. A serum sample will also be collected for the serum repository before each cycle of bortezomib and after the second cycle (W8), for later, more closely spaced analysis of DSA titer. This is an important consideration for the future follow-up of patients with chronic active AMR treated with bortezomib. It will help answer the question of whether increasing the number of bortezomib treatment cycles provides a benefit, as has been reported in the case of multiple myeloma. Observing a rebound in antibody titer some time after the treatment cycle, namely at six months and one year, will provide information on the durability of the response to treatment.

1.5.3. Risks

<u>1.5.3.1 Physical risks and burdens</u>

There may be serious risks associated with the use, in combination with steroids, of a new immunosuppressant – which acts on T and B cell function – in patients already receiving triple immunosuppressive therapy. The main risk associated with use in patients who are already immunocompromised is the occurrence of infections. For this reason, prophylactic

antibiotics (Bactrim®, Zelitrex®) will be routinely administered to patients in the bortezomib arm.
Patients in the control arm will receive the same prophylactic treatments, given that steroids and plasmapheresis are also used in this arm. Nevertheless, the chosen study population should limit this risk. This is because we have chosen to treat patients who did not have pre-existing DSA prior to transplantation and who did not experience any episodes of AMR during the current transplant. This therefore limits the use of potent immunosuppressive therapy prior to bortezomib. The use of high doses of steroids, except in cases of acute cellular rejection, should also be rare and none of these patients will have received rituximab treatment during the course of the current transplant.
The second risk associated with the combination of bortezomib and IMPDH inhibitors (mycophenolic acid), which most kidney transplant patients receive as maintenance therapy, is an increase in hematological toxicity, manifesting as central-origin cytopenias (thrombocytopenia, anemia, leucopenia).
First, we will review the adverse effects associated with the use of bortezomib, as reported in studies conducted during the treatment of multiple myeloma and other hematologic disorders. We will then consider the safety profile of bortezomib (Velcade®), using data from the few published studies on kidney transplant patients.

- <u>Adverse effects reported with the use of bortezomib for the treatment of multiple myeloma</u>

Table 6 in the summary of product characteristics in Appendix 4 lists all the adverse effects reported in connection with the use of bortezomib in the treatment of myeloma.
The following adverse effects, observed during the use of bortezomib in the treatment of myeloma, require precautions to be taken and will be the subject of particular monitoring during this study.

Gastrointestinal effects:

Gastrointestinal toxicity, such as nausea, diarrhea, vomiting, and constipation, are very common during treatment with bortezomib. As cases of ileus have been reported, patients suffering from constipation must be closely monitored.

Hematological effects:

Treatment with bortezomib is very frequently associated with hematological toxicity (thrombocytopenia, neutropenia, and anemia). The most common hematological toxicity is transient thrombocytopenia; platelet counts reached their lowest point on day 11 of each cycle of bortezomib treatment. There is no evidence of cumulative thrombocytopenia. The average of the nadir platelet counts measured was approximately 40% of the baseline value. In patients with advanced-stage myeloma, the severity of thrombocytopenia was linked to the pre-treatment platelet count. Before each bortezomib injection, platelet counts must be monitored. Patients with a platelet count of less than 100,000/µl will not be included and the platelet count must be above 100,000/µl before a decision is made regarding an injection. It is very rare to encounter such levels of thrombocytopenia in kidney transplant patients, and exceptionally so when they are associated solely with treatment with mycophenolic acid, compared with patients with myeloma, who are most often treated with myelosuppressive chemotherapy and have disease-related medullary failure.

Similarly, the neutrophil count must be above 1,500/µl before inclusion in the study. In the event of thrombocytopenia or neutropenia, the bortezomib cycle will be postponed by one week and administered once these hematological abnormalities have been corrected.

Progressive multifocal leukoencephalopathy (PML)

Very rare cases of infection with the John Cunningham (JC) virus with no known causal relationship that have led to PML and death have been reported in patients treated with Velcade®. Patients diagnosed with PML had received prior or concomitant immunosuppressive therapy.

Most cases of PML were diagnosed within 12 months of the first dose of Velcade®. Patients must be monitored at regular intervals to detect the onset or worsening of any neurological symptoms or signs suggestive of PMSL, as part of the differential diagnosis of CNS disorders. If PML is suspected, patients should be referred to a PML specialist and appropriate diagnostic tests should be performed. Discontinue Velcade® if PML is diagnosed.

Peripheral neuropathies: **see Appendix 5**

Bortezomib treatment is very commonly (in more than 10% of cases) associated with peripheral neuropathies, primarily sensory neuropathies. However, cases of severe motor neuropathies, whether or not associated with sensory peripheral neuropathy, have been reported. The incidence of peripheral neuropathies increases from the start of treatment and peaks during the fifth cycle. Our protocol, however, requires only two cycles.

A neurological examination, including an assessment of paresthesia, osteotendinous reflexes, tactile sensation in the hands and feet, and muscle strength, will be documented in the patient's medical record at the start of each bortezomib cycle and at three, six, and 12 months. Patients with new-onset or worsening peripheral neuropathy should undergo a specialized neurological evaluation and may require temporary discontinuation of bortezomib treatment. These neuropathies can be particularly debilitating in kidney transplant patients, who may have pre-existing neuropathy: uremic neuropathy, diabetic neuropathy, amyloid neuropathy, and neuropathy associated with calcineurin inhibitors (tacrolimus). Consequently, patients with severe neuropathy will not be included in the study.

Improvement or resolution of peripheral neuropathy upon discontinuation of treatment was reported in 51% of patients with grade 2 or higher peripheral neuropathy in the Phase III trial and in 71% of patients with grade 3 or 4 peripheral neuropathy, or peripheral neuropathy that led to discontinuation of treatment in the Phase II trials (see table in Appendix 5 for neuropathy grades).

In addition to peripheral neuropathies, disorders of the autonomic nervous system can contribute to adverse effects, such as orthostatic hypotension and severe constipation with ileus.

Seizures:

Seizures have been reported rarely in patients with no history of seizures or epilepsy. Close monitoring is required during treatment for patients with risk factors for seizures.

Hypotension:

Treatment with bortezomib is frequently associated with orthostatic hypotension. Most cases are mild to moderate in severity and may occur at any time during treatment.

Patients who developed orthostatic hypotension while taking bortezomib did not show any signs of orthostatic hypotension before treatment. The mechanism responsible for this reaction is unknown, but it may be related to a disorder of the autonomic nervous system. As with sensory neuropathy, the use of bortezomib is contraindicated in patients with pre-existing severe autonomic neuropathy, such as diabetic or amyloid neuropathy. Caution is advised when using bortezomib in patients with a history of syncope during hypotensive therapy or who are dehydrated (due to diarrhea, vomiting, etc.). Treatment of orthostatic

hypotension may include adjusting antihypertensive medications, rehydration, or the administration of mineralocorticoids and/or sympathomimetics. Patients should be advised to consult a doctor if they experience dizziness, vertigo, or episodes of syncope.

Cardiac adverse effects:

The onset or worsening of congestive heart failure has been reported infrequently or rarely during treatment with bortezomib. In a randomized, comparative Phase III study, the incidence of heart failure was similar in the bortezomib and dexamethasone groups. Sodium and fluid retention can be a contributing factor in the onset of signs and symptoms of heart failure. Patients with underlying cardiopathy must be closely monitored.

Pulmonary adverse effects:

Rare cases of acute diffuse infiltrative pneumopathy of unknown etiology, such as pulmonary inflammation, interstitial lung disease, pulmonary infiltrates, and acute respiratory distress syndrome (ARDS), have been reported in patients treated with bortezomib. Some of these occurrences were fatal. A chest X-ray will be performed before treatment begins to obtain a baseline image and to screen for any underlying pulmonary conditions.

Kidney failure:

It has been shown that the incidence of serious adverse effects increases in patients with mild to moderate kidney impairment, compared with patients with normal kidney function. These patients should be closely monitored and a reduction in dosage should be considered, if necessary.

Reactions potentially associated with immune complexes:

Reactions potentially associated with immune complexes, such as serum sickness-like reactions, polyarthritis with rash, and proliferative glomerulonephritis, have been reported infrequently. Bortezomib must be discontinued if serious reactions occur.

Precautions regarding certain concomitant treatments:

Patients receiving bortezomib in combination with potent CYP3A4 inhibitors (ketoconazole, ritonavir) or CYP3A4 inducers (rifampin, St. John's wort) are at risk of an increase or decrease, respectively, in the area under the curve for bortezomib. Consequently, treatment must be discontinued during bortezomib treatment insofar as possible. Precautions should be taken for patients receiving treatment with carbamazepine, phenytoin, or phenobarbital.

During clinical trials, hypoglycemia and hyperglycemia were reported infrequently and frequently, respectively, in diabetic patients receiving oral hypoglycemic agents. Patients receiving such treatment while also receiving bortezomib will require close monitoring of their blood glucose levels and adjustment of their blood glucose-lowering medication dose in the event of a change in its efficacy.

- <u>Adverse effects reported with the use of bortezomib in kidney transplantation</u>

The few studies published on the use of bortezomib in kidney transplantation report fairly good tolerability with no specific side effects, albeit in a small number of treated patients. Table 3 lists the adverse effects reported in publications discussing the use of rituximab in kidney transplantation.

Table 3: Adverse effects reported with the use of bortezomib in kidney transplantation

Reference Number of patients	Associated treatment	Adverse effect	Frequency
**Flechner (n=20)**	Steroids, plasmapheresis, IVIg	- SAE, including<br>Diarrhea<br>Edema<br>Fever<br>- Fatigue, lethargy<br>- Hyperkalemia<br>- Thrombocytopenia (35,000 to 100,000/mm³)<br>- Temporary numbness in the limbs	- N=7 (35%) N=3<br>N=3<br>N=1<br>- N=9 (45%)<br>- N=2 (10%)<br>- N=5 (25%)<br>- N=2
**Everly (n=6)**	Plasmapheresis, steroids, ATG, rituximab	- Diarrhea and grade II thrombocytopenia - Febrile neutropenia	- N=1<br>- N=1
**Trivedi (n=11)**	Plasmapheresis and rituximab (6/11)	- Diarrhea<br>- Thrombocytopenia (always > 50,000)<br>- General weakness	- N= 3 (30%)<br>- N=11 (100%)<br>- N=1
**Sberro-Soussan (n=4)**	None	- General weakness<br>- Bilateral conjunctivitis<br>- Vomiting	- N=3 (75%)<br>- N=3 (75%)<br>- N=1
**Walsh, 2011 (n=28)**	Bortezomib, plasmapheresis, rituximab	- Anemia<br>- Thrombocytopenia<br>- Neuropathy	- N=12 (43%)<br>- N=8 (29%)<br>- N=5 (18%)
**Waiser N=10**	Bortezomib, plasmapheresis, IVIg	- thrombocytopenia<br>- Diarrhea	- N=7 (70%)<br>- N=1 (10%)

1.5.3.2 Physical burden related to the experimental protocol

Patients included in the study will undergo five plasma exchanges and four three-week courses of intravenous immunoglobulin, each of which will require a three- to four-day hospital stay, depending on the speed of administered treatment, oral dexamethasone and, for the bortezomib arm only, two cycles of four intravenous bortezomib injections administered concurrently with the first two courses of immunoglobulin. For the first two cycles of bortezomib, the first injection or the first two injections of bortezomib will be administered during the hospital stay scheduled for the IVIg treatment. These cycles of bortezomib will be preceded by blood tests to assess, in particular, hematological, hepatic, and kidney function. The injection will be administered by direct intravenous injection. Except for the first injections of the first two cycles, all other bortezomib injections will be administered on an outpatient basis over the course of half a day. These eight outpatient visits therefore represent a physical burden. Intravenous injection of the product is well tolerated; rare cases of pain at the injection site and extravasation have been reported.

In addition to these visits, several tests will be necessary:

- a kidney biopsy of the graft one year after inclusion. The kidney biopsy performed prior to inclusion does not constitute an additional procedure, as it is routinely performed in clinical practice when DSAs develop, when there is a decline in graft function, or when proteinuria develops, or as part of a routine screening program by centers that perform screening biopsies. The one-year kidney biopsy assesses treatment efficacy in both groups and is a standard monitoring tool in the treatment of antibody-mediated rejection. The risks associated with kidney graft biopsies are well known: hematoma (1–8%), gross hematuria (0.2–9%), arteriovenous fistula (0.3–10.7%), and pain requiring analgesia (Schwarz, 2005, Furness, 2003). Biopsies are performed under ultrasound guidance in an outpatient setting. Patients are monitored for four hours after the biopsy, as all complications occur within this time frame. The most recent studies report no deaths or graft losses attributable to the biopsy. In just 0.1 to 1% of cases, post-biopsy complications require invasive procedures and hospitalization (blood transfusion, bladder catheterization due to gross hematuria, surgical drainage of a hematoma, interventional radiology for embolization) (Schwarz, 2005, Furness, 2003).

As part of the protocol, additional blood tests performed during the transplant follow-up visit will be carried out at inclusion, before each bortezomib injection as part of treatment monitoring, and at 3 months, 6 months, 9 months,

and twelve months. Apart from the additional blood samples taken as part of the protocol, this will not increase the frequency of study visits, as, depending on the post-transplantation period and the practices of each center, patients are generally seen at intervals ranging from once a month to once every four months.

1.5.3.3 Psychological burden

The psychological risks to which patients will be exposed include the possibility that bortezomib will prove ineffective, causing patients to lose hope of controlling the progression of chronic active AMR, a condition for which no current treatment has demonstrated any efficacy and which invariably leads to premature graft loss.

1.5.3.4 Socioeconomic burden

Any work absenteeism associated with IVIg treatments, plasmapheresis, and scheduled bortezomib injections (totaling 8 half-days) may pose a socioeconomic risk, once the transplant recipient has returned to work. Nevertheless, this risk must be weighed against the loss of the transplant and a return to dialysis, which, in addition to reducing the patient's survival and quality of life, imposes a much more long-lasting socioeconomic burden. Hemodialysis is often associated with a degree of general asthenia that impairs occupational performance, compared with kidney transplantation. The employment rate among hemodialysis patients is significantly lower than that of kidney transplant recipients.

2. STUDY OBJECTIVES

2.1. Primary objective

To evaluate, at one year, the efficacy and safety of bortezomib, in combination with steroids, plasmapheresis, and intravenous immunoglobulins (IVIg), on donor-specific anti-HLA antibody levels and on the progression of chronic active antibody-mediated rejection (AMR) lesions observed on kidney graft biopsy, in patients who underwent kidney transplantation more than 3 months previously.

2.2. Secondary objectives

The secondary objectives of the study are:

- to assess the effect of this one-year strategy on kidney graft function, graft survival, and patient survival
- To evaluate the safety of bortezomib in combination with steroids, plasma exchange, and IVIg in kidney transplant patients in terms of adverse events and infectious complications
- to assess the kinetics of DSA titer under bortezomib and at a later stage after the injections

3. SELECTION AND EXCLUSION OF STUDY PARTICIPANTS

3.1. Inclusion criteria

1 Patients over the age of 18 who have received a first or second ABO-compatible kidney transplant more than three months ago, from a deceased donor (whether or not the donor's heart was still beating) or from a living donor,

2 Patients with one or more donor-specific antibodies (DSA) that appeared de novo, i.e. that were absent on the day of transplantation and in pre-transplantation serum samples.

3 Patients with findings consistent with moderate chronic active antibody-mediated rejection (g+ptc > 2) on a graft biopsy performed as part of routine follow-up or for another reason:

- the occurrence of de novo DSA (threshold varies by laboratory):

In the absence of a validated threshold, the assessment of the MFI positivity threshold is left to the clinical teams at each center;

These de novo DSA may be anti-Cw or anti-DP antibodies

- and/or deterioration in kidney function.
- and/or proteinuria of more than 0.5 g/24 hours

**Criteria used for the histological diagnosis of moderate chronic active antibody-mediated rejection (Haas, 2013):**
- [glomerulitis score g + peritubular capillaritis score ptc]> 2<br>- and cg < 3, regardless of C4d staining

4 Patients who have been informed and have signed a written informed consent form

5 Given the teratogenic risks described in the SmPC for Velcade® and Cellcept®:

- Women of childbearing potential must have a negative pregnancy test on the day of inclusion. In addition, they must use at least one effective method of contraception before the start of treatment, during treatment, and for the entire duration of the study.

- during treatment and for at least 90 days after the last administration of an investigational medicinal product during the trial. In addition, according to the CellCept® SmPC, male patients or their female partners (rather than both partners) are recommended to use an effective method of contraception during treatment and for 90 days after the male partner's last dose of mycophenolate (ANSM recommendations: AL20151069 and AL20151070).

[Update and revision of the recommendations on contraception during treatment with CellCept® or Myfortic® (ANSM references: AL20180650/AL20180651)]

6 Patients who are registered with or covered by a social security scheme

7 Patients with cellular rejection lesions associated with chronic active antibody-mediated rejection lesions may be included in the study. This rejection can be treated with three 500-mg bolus doses of methylprednisolone prior to inclusion.

3.2. Exclusion criteria

1 Patients with a donor-specific antibody (DSA) present before transplantation or on the day of transplantation, or with a positive historical cross-match or a positive cross-match on the day of transplantation.

2 Patients receiving a third or fourth kidney transplant

3 Patients who have received a combined transplant with another non-kidney organ

4 Patients who have experienced an episode of acute antibody-mediated rejection during the current transplant

5 Patients with advanced graft dysfunction, defined as an estimated glomerular filtration rate (eGFR), calculated using the MDRD formula, of less than 20 mL/min/1.73 m²

6 Patients with histological lesions of antibody-mediated rejection that are too advanced, defined by a cg

glomerulopathy score of 3/3 (Haas, 2013)

7 Patients with severe neuropathy, thrombocytopenia of less than 100,000/mm³, neutropenia of less than 1,000/mm³ and/or an active, uncontrolled infection

8 Patients with chronic active hepatitis B (positive for HBs antigen and/or HBV DNA), chronic hepatitis C, and/or a known HIV infection

9 Patients with hypersensitivity to bortezomib, boron, or any of the excipients

10 Patients with severe liver failure or abnormal liver function tests (conjugated bilirubin levels more than three times the normal range, transaminase levels more than three times the normal range), acute diffuse infiltrative pneumonia, or pericardial involvement

11 Patients who are deemed incapable of understanding the protocol and adhering to it

12 Patients who are currently participating in another treatment protocol or are within the exclusion period of another protocol.

4. STUDY DESIGN

4.1. Description of the study methodology

This project is a comparative superiority **therapeutic** trial that is **randomized**, **controlled**, and **open-label** for patients and the healthcare providers administering the treatments.

This clinical trial is a controlled study: it compares treatment with plasma exchange, intravenous immunoglobulins, steroids, and optimization of maintenance immunosuppressive therapy, with or without two cycles of bortezomib. This study is being conducted as an open-label trial, given the cost of producing a placebo and the burdens involved in the number of injections that would be required for the control group. However, the primary efficacy endpoint will be assessed by a pathologist (review of the graft biopsy) and a clinical biologist specializing in histocompatibility (determination of DSA), both of whom will be blinded to treatment allocation.

4.2. Patient recruitment

Patients will be recruited through two pathways:

1. **Screening through DSA testing:** this is carried out routinely at 3 or 6 months, at 1 year during the first post-transplant year, and annually thereafter. When a DSA test is positive and its de novo nature is confirmed by the absence of anti-HLA antibodies on pre-transplant testing and on the day of transplantation, the patient is scheduled for a kidney biopsy. If the biopsy shows features of chronic active AMR, as described in the Inclusion Criteria section, the patient is invited for a study visit to verify eligibility, receive information about the study protocol, and provide written informed consent.

2. **Diagnosis of chronic active AMR on kidney biopsy:** a kidney graft biopsy may be performed as part of routine follow-up or when a patient develops a gradual decline in kidney graft function or de novo proteinuria. If the biopsy demonstrates features of chronic active AMR, as described in the Inclusion Criteria section, DSA testing is performed, or the most recent DSA test result is retrieved. If de novo DSA are identified, the patient is invited for a study visit to

verify eligibility, receive information about the study protocol, and provide written informed consent.

Graft biopsies taken during patient selection (for patients randomized in the trial) will be stored on each site for the duration of the study. They will then be sent to and examined centrally at the Department of Anatomic Pathology at the Centre Hospitalier Universitaire de Nantes (Dr Renaudin), along with the one-year end-of-study biopsies.

4.3. Patient selection/inclusion and follow-up

4.3.1. Selection/inclusion (D-10) - Randomization

- Once the diagnosis of chronic active AMR and the presence of de novo DSA have been confirmed, the patient is invited to attend a study visit. If the patient meets the selection criteria in principle, the protocol is explained to them, along with the expected benefits, the risks involved, and how the study will be conducted. The investigator obtains the patient's signature on the informed consent form. The following tests are carried out to check the patient's eligibility. Once the investigator has obtained these results, he/she will check the inclusion and exclusion criteria. Randomization will then be carried out in the patient's absence, prior to the first plasma exchange, which must be scheduled as soon as possible and no later than 10 days after inclusion. The patient will be informed of their treatment arm during this first plasma exchange (D0) (plasma exchanges are performed in both treatment arms).
- Tests performed at the inclusion visit: clinical examination (blood pressure, weight, heart rate, neurological questionnaire)/Treatments received/Tacrolimus trough level/Biochemistry (electrolytes, urea, serum creatinine, liver function tests: total and conjugated bilirubin, transaminases, alkaline phosphatase, and gamma-GT, 24-hour proteinuria and creatininuria, eGFR calculated using MDRD)/CBC, PT, aPTT, fibrinogen/Identification of anti-HLA antibodies by Luminex single-antigen assay/Quantitative assay (IgG; IgA and IgM)/Chest X-ray/Plasma β-hCG pregnancy test/HBV and HCV viral serologies /T-cell (CD4/CD8) and B-cell (CD19/IgD/CD38/CD27) phenotyping/DNA repository/Serum repository

4.3.2. Patient follow-up

<u>4.3.2.1 Follow-up visits until the 8th week</u>

The five plasma exchanges must take place within a maximum period of 10 days.

**2nd visit** (**D14** ± 3 days)<br>**= D1 of the 1st course of bortezomib (bortezomib arm) and D1 of the 1st course of IVIg (2 arms)**	Clinical examination (blood pressure, weight, temperature, heart rate)/ Neurological examination/Treatments received/Tacrolimus trough level/Biochemistry (electrolytes, urea, serum creatinine, liver function tests, 24-hour proteinuria and creatininuria, eGFR calculated using MDRD)/CBC, PT, aPTT, fibrinogen/CRP/Serum repository
**3rd to 5th visits (D17, D21, D24) = D4, D8, D11 of the 1st course of bortezomib (bortezomib arm only)**	Each bortezomib injection involves a visit during which the following tests are performed:<br>Clinical examination (blood pressure, weight, heart rate)/ Biochemistry (electrolytes, urea, serum creatinine, liver function tests, 24-hour proteinuria and creatininuria on a spot urine sample, MDRD calculation)/CBC

**6th visit (D35)**<br>**= D1 of the 2nd course of bortezomib (bortezomib arm) and D1 of the 2nd course of IVIg (2 arms)**	Clinical examination (blood pressure, weight, temperature, heart rate)/ Neurological examination/Treatments received/Residual rate of trough level/Biochemistry (electrolytes, urea, serum creatinine, liver function tests, 24-hour proteinuria and creatininuria, eGFR calculated using MDRD)/CBC, PT, aPTT, fibrinogen/Serum repository/CRP
**7th to 9th visits (D38, D42, D45) = D4, D8, D11 of the 2nd course of bortezomib (bortezomib arm only)**	See 3rd to 5th visits: same tests as for the 1st course<br>From the W8 visit, the visit calendar is the same for both groups.

4.3.2.2 <u>Common schedule for both arms from W8 to M12</u>

**Visits in W8, W11, M6, M9*:**	Clinical examination (blood pressure, weight, heart rate)/Neurological examination/Treatments received/Tacrolimus trough level/Biochemistry (electrolytes, urea, serum creatinine, liver function tests, 24-hour proteinuria and creatininuria, eGFR calculated using MDRD)/CBC/CRP<br><u>Only in W8:</u> identification of anti-HLA antibodies by Luminex single-antigen assay/T- and B- cell phenotyping<br><u>Only in M6:</u> identification of anti-HLA antibodies by Luminex single-antigen assay/quantitative immunoglobulin assay/T- and B-cell phenotyping
**End of study visit at 12 months*:**	Clinical examination (blood pressure, weight, heart rate)/Neurological examination/Treatments received/Tacrolimus trough level/Biochemistry (electrolytes, urea, serum creatinine, liver function tests, 24-hour proteinuria and creatininuria, eGFR calculated using MDRD)/CBC/Serum repository/Identification of anti-HLA antibodies by Luminex single-antigen assay/Quantitative immunoglobulin assay/CRP/Kidney biopsy/T- and B-cell phenotyping

*A delay is accepted between the scheduled visit and the actual visit for visits between W8 and M12:

- ± 2 days for the W8 and W11 visits
- ± 1 week for the M6, M9, and M12 visits

4.3.3 Procedures and tests performed as part of standard care

All procedures and tests performed during the inclusion visit, on D0, then on D1 and D3/4 of the immunoglobulin courses for both groups, on D18–20 and D39–41 of the immunoglobulin courses for the control group, and the visits in W8, W11, M6, M9, and M12 are considered part of standard care, with the exception of the procedures and tests listed in paragraph 4.3.4 below.

4.3.4 Procedures and tests performed as part of the study

Visits on D21, D24, D42, and D45 for patients in the bortezomib arm are specific to the study.

Similarly, the procedures/tests listed below are related to the study:

- anti-HLA antibody assay at inclusion
- quantitative immunoglobulin assay at inclusion, in M6, and M12
- lymphocyte phenotyping in W8, M6, and M12.
- beta HCG (women only)
- DNA repository: one 7 mL tube at the inclusion visit
- Serum repository: one 7 mL tube at the inclusion visit and D14, D35, M12
- Samples taken for the pharmacokinetic study (two blood samples of 5 mL each)

4.3.5 Study design diagram and summary table

Study design diagram

Days are given for guidance only. In particular, the organization and performance of plasma exchanges may involve variable timeframes. The blood volumes collected during each visit are listed in the table below.

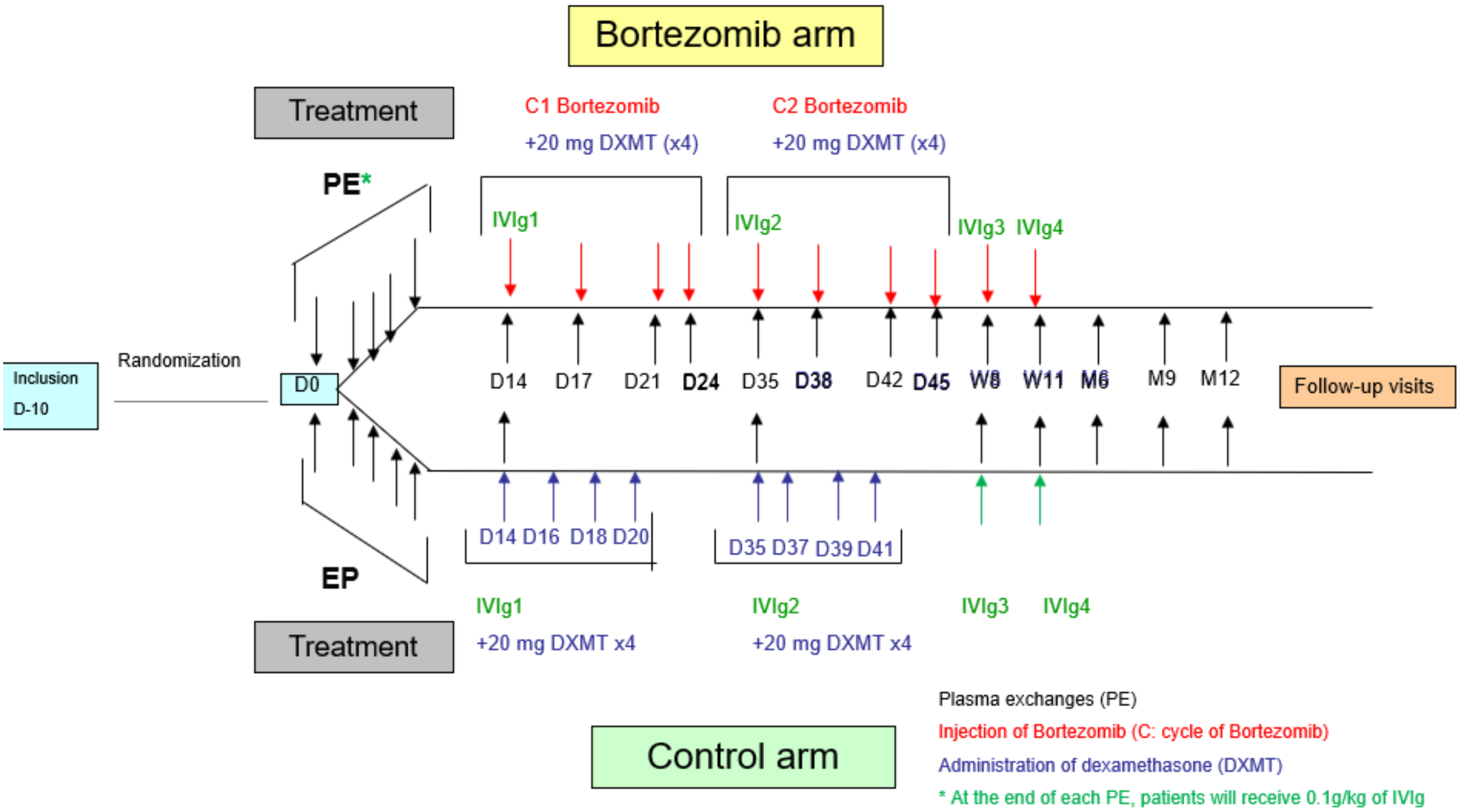

STUDY SCHEDULE FOR EACH INCLUDED PATIENT

Period	INCLUSION	RANDO	TREATMENT											FOLLOW-UP		
Title [c]	INCLUSION	RANDO	PE	C1/D1 IVIg 1	C1/D4	C1/D8	C1/D11	C2/D1 IVIg 2	C2/D4	C2/D8	C2/D11	IVIg 3	IVIg 4			
Visit No.	1[a]			2[a]	3[b]	4[b]	5[b]	6[a]	7[b]	8[b]	9[b]	10[a]	11[a]	12[a]	13[a]	14 and/or EOS[a]
Day(D) Week(W) Month(M)	D-10	D-10–D0	D0–D10	D14	D17	D21	D24	D35	D38	D42	D45	W8	W11	M6	M9	M12
Window			D0-D10	± 3 days												
Inclusion/exclusion criteria	X															
Information - Consent	X															
CleanWeb randomization		X														
Treatments (PE, IVIg, bortezomib)				X	X	X	X	X	X	X	X	X	X			
Clinical examination (weight, T°, BP, HR)	X			X	X	X	X	X	X	X	X	X	X	X	X	X
Neurological examination	X			X				X				X	X	X	X	X
Height, body surface area	X															
Adverse events (excluding SAE)				X	X	X	X	X	X	X	X	X	X	X	X	X
SAE	X			X	X	X	X	X	X	X	X	X	X	X	X	X
Biochemistry (blood, urine), liver function	X			X	X	X	X	X	X	X	X	X	X	X	X	X
CBC, platelets	X			X	X	X	X	X	X	X	X	X	X	X	X	X
PT, aPTT, fibrinogen	X			X												
T°, tacrolimus	X			X				X				X	X	X	X	X
CRP	X			X				X				X	X	X	X	X
Hepatitis B and C serologies	X															
Beta HCG	X															
Luminex anti-HLA antibodies	X											X		X		X
Quantitative immunoglobulin assay (IgG,	X													X		X
Chest X-ray	X															
Lymphocyte phenotyping (T and B cells)	X											X		X		X
Serum repository	X			X				X								X
DNA repository	X															
Kidney biopsy																X
Concomitant treatments	X			X				X				X	X	X	X	X
Plasma exchanges			X													
Ancillary tests[d]			X (2.3)										X (3)			X (1.2)
Pharmacokinetics (see section 4.6.2)			2 blood samples of 5 mL per patient													

a: visit for both arms/b: applies only to the bortezomib arm/c: bortezomib cycles: C1 and C2, days of the cycle: D1, D4, D8, D11/d: 3 ancillary studies per patient

*Liver function tests: total and conjugated bilirubin, transaminases, alkaline phosphatase, and gamma-GT

EOSV: End of Study Visit; **SAE:** Serious Adverse Events

SUMMARY TABLE OF SAMPLES FOR EACH INCLUDED PATIENT

Period	INCLUSION	TREATMENT									FOLLOW-UP				
Visit	Inclusion[a]	Plasma exchanges	2nd visit [a]	3rd visit [b]	4th visit [b]	5th visit [b]	6th visit [a]	7th visit [b]	8rd visit [b]	9th visit [b]	10th visit [a]	11th visit [a]	12th visit [a]	13th visit [a]	14th/ EOS [a]
Day(D) Week(W) Month(M)	D-10	D0	D14	D17	D21	D24	D35	D38	D42	D45	W8	W11	M6	M9	M12
Title [c]			**C1/D1 IVIg 1**	**C1/D4**	**C1/D8**	**C1/D11**	**C2/D1 IVIg 2**	**C2/D4**	**C2/D8**	**C2/D11**	**IVIg 3**	**IVIg 4**			**C1/D1 IVIg 1**
**Biochemistry (blood, urine), liver function tests***	*4 mL*		*4 mL*	*4 mL*	*4 mL*	*4 mL*	*4 mL*	*4 mL*	*4 mL*	*4 mL*	*4 mL*	*4 mL*	*4 mL*	*4 mL*	*4 mL*
**CBC, platelets**	*2.5 mL*		*2.5 mL*	*2.5 mL*	*2.5 mL*	*2.5 mL*	*2.5 mL*	*2.5 mL*	*2.5 mL*	*2.5 mL*	*2.5 mL*	*2.5 mL*	*2.5 mL*	*2.5 mL*	*2.5 mL*
**PT, aPTT, fibrinogen**	*4.5 mL*		*4.5 mL*												
**T°, tacrolimus**	*2 mL*		*2 mL*				*2 mL*				*2 mL*	*2 mL*	*2 mL*	*2 mL*	*2 mL*
**Hepatitis B and C serologies**	*5 mL*														
**βHCG**	*3.5 mL*														
**Luminex anti-HLA antibodies**	*5 mL*										*5 mL*		*5 mL*		*5 mL*
**Quantitative immunoglobulin assay ** **	*5 mL*												*5 mL*		*5 mL*
**Lymphocyte phenotyping (T and B cells)**	*2.5 mL*										*2.5 mL*		*2.5 mL*		*2.5 mL*
**Serum repository**	7 mL		7 mL				7 mL								7 mL
**DNA repository**	7 mL														
**TOTAL**	**49 mL**	**0 mL**	**20 mL**	**6.5 mL**	**6.5 mL**	**6.5 mL**	**15.5 mL**	**6.5 mL**	**6.5 mL**	**6.5 mL**	**17 mL**	**7.5 mL**	**22 mL**	**8.5 mL**	**29 mL**
+ Ancillary tests[d]		*17 mL(2)* *44 mL(3)*										*34 mL(3)*			*17 mL(2)*
+ Pharmacokinetics	2 blood samples of 5 mL per patient														

a: visit for both arms

b: applies only to the bortezomib arm

c: cycles of bortezomib: C1 and C2, days of the cycle: D1 D4 D8 D11

d: 3 ancillary studies per patient

*Liver function tests: total and conjugated bilirubin, transaminases, alkaline phosphatase, and gamma-GT

**Quantitative immunoglobulin assay: IgG, IgA, and IgM

4.4. RANDOMIZATION METHOD

After the patient has signed the informed consent form and the inclusion and exclusion criteria have been verified, the investigator will complete an electronic inclusion form, which is accessible 24 hours a day on the Internet via the e-CRF (secure access to which has been obtained in advance by each pre-registered investigational center).
Randomization lists will be generated using the block method.
Randomization will then be performed using a block randomization method, stratified by center and by biopsy type (clinical or subclinical chronic active AMR). These two populations may follow different trajectories. Patients who already have graft dysfunction or proteinuria (clinical chronic active AMR) may have a more advanced form of the condition and therefore lower chances of success compared to patients with subclinical forms of the condition.
Randomization will be computerized and linked to the electronic data collection system, so that patients included in the study will be easily and automatically randomized.

4.5. SITE OF EXAMINATIONS, SAMPLE COLLECTION, AND ASSAYS

4.5.1 Centralized tests and assays:

The kidney graft biopsy slides required for selection, as well as those performed at 12 months, will be transported by an approved carrier, either at the end of the study or during the study (group shipment), to the Department of Pathology and Cytology at the Centre Hospitalier Universitaire Hôtel-Dieu in Nantes, where they will be re-examined under the direction of Dr. Renaudin. Four slides with different stains must be sent: HES, PAS, Masson’s trichrome, and silver staining.
Blood samples intended for anti-HLA antibody testing will be transported at the end of the study or during the study (group shipment) by an approved carrier to the Établissement Français du Sang Rhône-Alpes in Lyon. Antibodies are collected in gel tubes (5 mL), centrifuged (2300 g, 15 mins), and frozen as is at -20°C. HLA assays will be performed under the responsibility of Dr. Valerie Dubois.

4.5.2 Local tests and assays:

All additional tests specified in the sample collection table, other than anti-HLA antibodies, will be performed at the participating study centers.

4.6. Sample storage

4.6.1 Samples included in the Kidney Transplantation Biocollection

The samples in question are those that make up the DNA repository and the serum repository (see the table of samples). The DNA repository sample consists of a 7 mL EDTA tube, stored frozen on site at -20°C without centrifugation. The serum repository consists of a 7 mL dry tube, centrifuged and aliquoted into four tubes of 500 µL each, plus one tube containing the remaining volume. All tubes will be transported at the end of the study or during the study (group shipment) by

an approved carrier.

DNA repository: The purpose of collecting DNA samples is to enable local histocompatibility laboratories to perform high-resolution HLA typing using molecular biology techniques for all patients. This typing will notably allow for the study of the role of non-classical donor-specific antibodies, directed in particular against HLA-DQA and DP antigens, since DQA and DP typing is not performed routinely.

Serum repository: the purpose of storing serum is to be able, if necessary, to perform specific biological assays that were not included in routine laboratory testing and that may be influenced by the investigational treatment. These samples may allow, if necessary and based on the study's initial results, additional anti-HLA antibody assays to be performed using the Luminex single-antigen method, as well as quantitative immunoglobulin assays.

At the end of the study, the samples will be stored for a period of 30 years on site in the Tissue Repository–DNA Repository–RNA Repository in the laboratory of the Department of Pathology at Hôpital Necker-Enfants Malades, under the responsibility of the Coordinating Investigator (Dr. Renaud Snanoudj) and the site manager (Prof. Thierry Molina).

4.6.2 Samples taken for the pharmacokinetic study

For the pharmacokinetic study, blood samples (5 mL) will be taken from five groups of five different patients:

- **Group 1**: in five patients in the bortezomib arm, a blood sample will be taken five minutes and then 15 minutes after the first injection of Cycle 1.
- **Group 2**: in five patients in the bortezomib arm, a blood sample will be taken two hours and then six hours after the last injection of Cycle 1.
- **Group 3**: in five patients in the bortezomib arm, a blood sample will be taken 30 minutes after the last injection of Cycle 1 and just before the first injection of Cycle 2 (residual concentration).
- **Group 4**: in five patients in the bortezomib arm, a blood sample will be taken 24 hours after the first injection of Cycle 2 and just before the second injection of Cycle 2 (trough concentration).
- **Group 5**: in five patients in the bortezomib arm, a blood sample will be taken just before the third injection of Cycle 2 (trough concentration) and 10 days after the last injection of Cycle 2.

This study will be conducted under the supervision of Prof. Treluyer in the Developmental Biology Laboratory of the Paris Centre Hospital Group (Cochin, Broca, and Hôtel-Dieu).

The optimal sampling times were determined using PFIM software (http://www.pfim.biostat.fr/), based on the pharmacokinetic parameters reported by Millennium Pharmaceuticals, Inc. (Kuan, 2012) for bortezomib and the study by Nakade et al. (2008) for dexamethasone.

4.7. Study duration

The recruitment period will last 54 months. The follow-up period will last 12 months for each patient included in the study. The total duration of the study will therefore be 66 months.

4.8. Identification of collected data

All patient-related data will be collected by the investigators at each study center.

Identification data: center number, inclusion number, patient initials (first letter of the surname followed by the first letter of the first name),

Demographic data: sex, month, and year of birth,

Medical data: weight, relevant medical history, usual medications,

Efficacy data: HLA antibody testing, review of kidney graft biopsy slides,

Safety data: occurrence of AE or SAE.

The patient's clinical and laboratory records, together with any electronic or paper-based means used to collect these data, shall be considered source documents and must be retained by the investigational sites to enable subsequent verification and any audits.

5. TREATMENTS ADMINISTERED TO STUDY PARTICIPANTS

5.1. Description of the bortezomib treatment required to conduct the study: Description of bortezomib (Velcade®)

- Bortezomib, mannitol boronic ester for injection.
- Antineoplastic agent. Bortezomib (Velcade®) is indicated in combination with melphalan and prednisone for the treatment of previously untreated patients with multiple myeloma who are not eligible for high-dose chemotherapy with bone marrow transplantation.
- Bortezomib (Velcade®) is indicated as monotherapy for the treatment of progressive multiple myeloma in patients who have received at least one prior treatment and who have either already undergone or are ineligible for a bone marrow transplant.
- European Marketing Authorization Number for this indication (EU/1/04/274/002) (2008, rev. 06/2010)
- Marketing Authorization holder: JANSSEN-CILAG INTERNATIONAL NV, Beerse, Belgium.
- Local representative in France: JANSSEN-CILAG, Issy-les-Moulineaux, Cedex 9
- Velcade®, a trademark of Millennium Pharmaceuticals, Inc.

5.2. Composition, dosage, and administration of the bortezomib (Velcade®) treatment

- Pharmaceutical form: Sterile lyophilized powder for reconstitution for injection.

A 10-mL vial containing 3.5 mg of bortezomib and a 5-mL vial containing 1 mg of bortezomib.

- Composition: active ingredient = bortezomib + excipients: Mannitol (E421) + nitrogen

- Dosage: The treatment consists of two courses of four bortezomib (Velcade®) injections administered three weeks apart, with one injection every three days at a dose of 1.3 mg/m², capped at 2 m² for patients in the Velcade arm; combined with 20 mg of dexamethasone. Bortezomib injections should be administered on D1, D4, D8, and D11 of both cycles; there is no flexibility regarding the timing.

- Dose reduction: see table in the section **Bortezomib (Velcade®) dose adjustment in the event of toxicity** – 1) Neurotoxicity.

- Store at a temperature not exceeding 30°C and in the original packaging, protected from light.

- Product preparation: Bortezomib is a cytotoxic agent. Precautions must be taken when handling it. The use of gloves or other protective clothing is recommended to prevent contact with the skin. Aseptic technique must be strictly observed when handling bortezomib (Velcade®) because it does not contain any preservatives. The powder for solution should be dissolved in a 0.9% sodium chloride injectable solution to achieve a final concentration of 1 mg/mL of bortezomib (i.e. 3.5 mL for the 3.5-mg vial). The reconstituted solution is clear and colorless, and must be visually inspected before injection to ensure that it is free of particles and has not changed color. If a change in color or particles is observed, the reconstituted solution must be discarded.

Immediate use recommended

- Storage of the reconstituted product: below 25°C for a maximum of 8 hours.

- Administration: The reconstituted solution is administered by intravenous bolus injection over 3 to 5 seconds into a peripheral or central intravenous catheter, followed by flushing with 0.9% sodium chloride injection.

A nurse-administered treatment monitoring form will be provided in the study for patients in the Velcade® arm. It will be completed by the nurse at the time of administration of each product and on each day of treatment. It will then be kept on file in the patient's medical record.

Bortezomib (Velcade®) dose adjustment in the event of toxicity

If the first course of bortezomib is interrupted due to toxicity, the second course will be administered, along with the second course of IVIg, 10 days after the end of the first course.

If the second course of bortezomib is interrupted due to toxicity, the third course of IVIg will be administered 10 days after the end of the second course of bortezomib.

1 - Neurotoxicity (see Appendix 5)

In the event of signs of neurotoxicity occurring after the first cycle of bortezomib, doses should be adjusted as follows for the second cycle:

Neuropathy severity	Dosage adjustment in the second cycle
**Grade 1**: Paresthesia, muscle weakness and/or loss of reflexes, without loss of function and without pain	No change to the dosage
**Grade 1** with pain or<br><br>**Grade 2**: Objective sensory and/or motor loss resulting in functional impairment and limiting instrumental activities of daily living[1]	Reduce the bortezomib (Velcade®) to 1 mg/m^2
**Grade 2** with pain<br><br>**Or Grade 3**: Impairment of autonomy[2]	Suspend treatment with bortezomib (Velcade®) until the symptoms have resolved. Then resume treatment with bortezomib (Velcade®), reduce the dosage to 0.7 mg/m^2, and reduce the frequency of administration to once a week.
**Grade 4**: Disabling sensory neuropathy and/or motor neuropathy that is life-threatening or leads to paralysis and/or severe autonomic neuropathy.	Discontinue treatment with bortezomib (Velcade®)

1: **The Instrumental Activities of Daily Living (**IADL) scale refers to meal preparation, grocery or clothing shopping, telephone use, and money management,
2: **The Autonomy Activities of Daily Living** scale refers to personal hygiene, dressing and undressing, feeding oneself independently, using the toilet, taking medication, and not being bedridden.

2 - Dosage adjustment in the event of abnormal liver function tests

Liver function tests are performed at the start of each cycle to adjust the bortezomib (Velcade®) dose if necessary, in accordance with the protocol below. Liver function tests are then carried out after each injection as a routine monitoring measure.

If total bilirubin levels increase to up to 1.5 times the upper limit of the normal range, the dosage of bortezomib (Velcade®) is not adjusted.

If serum bilirubin levels increase to between 1.5 and 3 times the normal range, the dose should be reduced to 0.7 g/m^2 per injection.

If serum bilirubin levels increase to more than 3 times the normal range at the start of the second cycle, treatment must be suspended until liver function tests return to normal (one analysis per week) and resumed at 0.7 mg/m^2.

If transaminase levels increase to more than 3 times the normal range, treatment must be suspended until liver function tests return to normal (one analysis per week) and resumed at 0.7 mg/m^2.

3 - Adjustment in the event of hematologic toxicity

A blood count will be performed on each day that bortezomib is administered and the results must be available before the injection is given.

Neutrophils/mm³	Platelets/mm³	Adjustment
< 500	< 50,000	Permanent discontinuation of bortezomib
500–1,000	50,000–100,000	Discontinuation of the cycle<br>Weekly CBC monitoring<br>Resumption of injections at 1 mg/m²
>1,000	100,000	No adjustment; continuation of the cycle

5.3. Description of the combination therapies in the experimental arm (bortezomib) and the control arm

5.3.1 Treatment regimen for the experimental arm

It may take a few days to organize plasma exchanges (up to 10 days after the inclusion visit). The following schedule is therefore approximate:

**D-10**	- Inclusion
**D0**	- Randomization
**D0–D10**	- 5 plasma exchanges (PE) + 0.1 g/kg IVIg at the end of each PE<br>- Adjustment of immunosuppressive therapy, if necessary<br>- Start of anti-infective prophylaxis
**D14**	- 1st course of IVIg and<br>- 1st course of bortezomib: 1.3 mg/m² on D1course/D4course/D8course/D11course +<br>- Dexamethasone per os (solution or scored tablets): 20 mg with each bortezomib injection
**D35**	- 2nd course of IVIg and<br>- 2nd course of bortezomib: 1.3 mg/m² on D1course/D4course/D8course/D11course +<br>- Dexamethasone per os (solution or scored tablets): 20 mg with each bortezomib injection
**W8**	- 3rd course of IVIg
**W11**	- 4th course of IVIg

5.3.2 Treatment regimen for the control arm

**D-10**	- Inclusion
**D0**	- Randomization

**D0–D10**	- 5 plasma exchanges (PE) + 0.1 g/kg IVIg at the end of each PE<br>- Adjustment of immunosuppressive therapy, if necessary<br>- Start of anti-infective prophylaxis
**D14**	- 1st course of IVIg and<br>- Dexamethasone per os (solution or scored tablets): 20 mg every 48 hours from the beginning of the course: D14, D16, D18, and D20
**D35**	- 2nd course of IVIg and<br>- Dexamethasone per os (solution or scored tablets): 20 mg every 48 hours from the beginning of the course: D35, D37, D39, and D41
**W8**	- 3rd course of IVIg
**W11**	- 4th course of IVIg

5.3.3 Plasma exchanges

Five plasma exchanges will be performed over a 10-day period, in accordance with the center's standard practice, using a dose of 60 mL/kg of body weight of treated plasma. Each plasma exchange is followed by the infusion of an intravenous dose of immunoglobulin at a dosage of 0.1 g/kg. Insofar as possible, it is preferable not to wait more than two full days between plasma exchanges. These plasma exchanges will be performed at a day clinic or any other outpatient facility, depending on each center's practices. Plasma exchanges must be performed within the two weeks preceding the start of bortezomib treatment.

5.3.4 Intravenous immunoglobulin

An intravenous infusion of 0.1 g/kg of immunoglobulin is administered at the end of each of the five plasma exchanges. Then four courses of IVIg at a dose of 2 g/kg of body weight will be administered at three-week intervals. The interval between administrations, which will not be less than 48 hours, will depend on the participating centers and the patients' tolerance of the infusion rate. Depending on the practices at each center, prophylactic heparin therapy may also be used. The IVIg typically used by each center may be used, provided they do not contain sucrose, to limit the risk of nephrotoxicity.

5.3.5 Steroids

20 mg of dexamethasone will be administered per os (ampoule or scored tablets) before or after each bortezomib injection in the bortezomib arm, i.e. on D14, D17, D21, and D24 (1st cycle) and D35, D38, D42, and D45 (2nd cycle) and during the first two courses of IVIg in the control arm, i.e. on D14, D16, D18, D20 and D35, D37, D39, and D41.

5.3.6 Anti-infective prophylaxis

Prophylaxis with Bactrim® (sulfamethoxazole + trimethoprim, at a dose of 400 mg/day or 800 mg, three times per week) in both groups and Zelitrex® (valaciclovir, at a dose of 1 g per day for subjects with normal kidney function) in the bortezomib group will be administered to patients for a period of six months following inclusion, unless they are allergic to either of these anti-infective agents. At six months, Bactrim® prophylaxis will be continued if the CD4+

T-cell count is less than 200/mm³.

5.4. Adjustment and optimization of maintenance immunosuppressive therapy

The treatment identification forms are included in Appendix 6.

Maintenance immunosuppressive therapy for patients in the control group and the bortezomib group will need to be adjusted if it does not consist of a triple combination of prednisone or prednisolone, tacrolimus, and mycophenolate mofetil or mycophenolate sodium. This adjustment must be made within two weeks of randomization in both groups. This adjustment will be made during the plasma exchange phase, which will allow for any necessary tacrolimus level measurements and routine laboratory monitoring (kidney function, complete blood count) to be performed at the start of each plasma exchange session.

In practice, if patients are not receiving steroids, prednisone or prednisolone should be started immediately at a dose of 10 mg/day, taken as a single dose. If the daily dose of prednisone/prednisolone is less than 10 mg, the dose should be increased to 10 mg per day, taken as a single dose.

If patients are not receiving a prodrug form of mycophenolic acid (CellCept® or Myfortic®), one of these two medications should be started at a dose of 500 mg twice daily for CellCept® or 360 mg twice daily for Myfortic®, unless there is severe gastrointestinal intolerance to either drug. The switch will take place without an overlap period.

If patients are not receiving tacrolimus, it must be introduced at the dosage appropriate for the tacrolimus formulation used, replacing cyclosporine (Neoral®) or an mTOR inhibitor (Rapamune®, Certican®), to achieve a trough concentration of between 6 and 10 ng/mL. The switch will take place without an overlap period, except possibly when switching from an mTOR inhibitor to tacrolimus.

5.5. Investigational medicinal product management

The relabeling of the investigational drug and the centers' supply will be handled by the APHP's Clinical Trials Department.

Every time a patient is randomized to the Velcade arm, the eCRF will send a supply request for eight boxes of one vial of Velcade® 3.5 mg to the Clinical Trials Department of the General Agency for Healthcare Equipment and Products (AGEPS). All the treatment required for the included patient will be delivered to the hospital's internal pharmacy within five business days.

Dispensation: Every time a patient is included in the Velcade arm and after the pharmacy has received the patient's medication, it will be dispensed to the Clinical Department upon presentation of a prescription made out in the patient's name.

Drug accountability

- Destruction: Cytotoxic product. Any unused product or waste must be disposed of in accordance with applicable regulations.
- Unused products will be destroyed on site after accountability checks and review by the study's Clinical Research Associate.

5.6. Accountability procedures for the investigational medicinal product(s)

Traceability data must be recorded in each patient's case report form.

5.7. Medications and treatments that are permitted and prohibited under the protocol, including rescue medications.

Patients receiving bortezomib in combination with potent CYP3A4 inhibitors (ketoconazole, ritonavir) or CYP3A4 inducers (rifampin, St. John's wort) are at risk of an increase or decrease, respectively, in the area under the curve for bortezomib. Consequently, treatment must be discontinued during bortezomib treatment. However, since HIV infection is an exclusion criterion, Ritonavir should not be prescribed to patients included in the Tribute study.
Precautions should be taken for patients receiving treatment with carbamazepine, phenytoin, or phenobarbital.
If the patient is taking oral antidiabetic medications, his/her diabetes will need to be monitored more closely.

5.8. Patient card

In accordance with the obligations of the Good Manufacturing Practice guidelines of May 26, 2006, a TRIBUTE patient card will be systematically given to the patient at the inclusion visit, bearing the statement "Please keep this card with you at all times," and specifying the name, address, and telephone number of the investigational center and the study's coordinating center (Pôle Médecine, Hôpital Necker), the study code, the patient identification code, the treatment, the dosage, and the treatment start date.

6. EFFICACY ASSESSMENT

6.1. Primary efficacy endpoints

The primary endpoint is a one-year efficacy endpoint combining:

1) **a decrease in the mean fluorescence intensity of the immunodominant donor specific antibody (i.e. with the highest MFI) of more than 50% compared to the value at inclusion.** For all patients, a 5-mL dry tube of blood will be collected at inclusion, at six months and at one year. The tubes will be centrifuged, the serum will be frozen, and all samples (from inclusion and end of study, for all patients) will be analyzed centrally, using the Luminex technique to avoid variations related to the technique and to the reagent lots used (Établissement Français du Sang in Lyon, Dr. Valérie Dubois). For each sample, the number of DSA, their specificities, and their respective mean fluorescence intensities will be recorded;

AND

2) **stabilization or improvement of the histological lesions indicative of chronic active antibody-mediated rejection observed on the graft biopsy at one year.** The biopsy will be performed as an outpatient procedure. After ultrasound guidance and local anesthesia with xylocaine, a kidney biopsy will be performed using a 16G needle. Two fragments will be taken. One will be fixed in AFA fixative (alcohol-formalin-acetic acid) for light microscopy analysis, and the other will be frozen for immunofluorescence analysis. *The review of one-year biopsies will be centralized (Centre Hospitalier Universitaire Hôtel-Dieu in Nantes) by a pathologist who is not aware of the treatment group corresponding to the specimen.* The criteria used are detailed in Section 2.1.2, Table 1. These are microcirculation

abnormalities (glomerular capillaritis, peritubular capillaritis, allograft glomerulopathy), each graded from 0 to 3 according to the 2013 Banff classification (see Appendix 3).

Stabilization or improvement of the histological lesions of chronic active AMR is defined by the following change in the glomerulitis and peritubular capillaritis scores (g+ptc), and the allograft glomerulopathy score (cg):

- $\Delta(g + ptc) \leq 1$ **and** $\Delta cg < 1$ between the biopsy at inclusion and the biopsy at one year

C4d staining, which is part of the definition of chronic active AMR according to the Banff classification, will be recorded and graded (see Appendix 3), but does not constitute a necessary criterion for diagnosing chronic active AMR.

6.2. Secondary efficacy endpoints

The secondary endpoints are as follows:

1) 1) each of the efficacy endpoints considered separately that make up the composite primary endpoint at one year, namely the reduction in the titer of the immunodominant DSA and the stabilization or improvement of histological lesions of chronic active AMR.

2) 2) change in the titer of each DSA and the sum of DSA

3) 3) Changes in the mean fluorescence intensity of DSA at 6 months to measure the kinetics of alloantibody responses to bortezomib, and at corresponding points in the control group

4) 4) All other acute and chronic histological lesions according to the Banff 2013 classification (Haas 2013) on the initial biopsy and the one-year biopsy:

- - signs of acute cellular rejection (interstitial infiltration, tubulitis, arteritis) and overall inflammation
- - signs of acute antibody-mediated rejection other than capillaritis: glomerular thrombosis, acute tubular necrosis
- - chronic vascular lesions (fibrous endarteritis and arteriolar hyalinosis)
- - interstitial fibrosis and tubular atrophy
- - chronic cellular rejection: chronic allograft arteriopathy (intimal fibrosis with mononuclear cell infiltration and neointimal formation)

5) Changes in kidney function assessed by 24-hour proteinuria, the proteinuria-to-creatinine ratio, serum creatinine, and glomerular filtration rate (GFR) estimated using the MDRD formula:

$$\textbf{eGFR} = 175 \times (\text{serum creatinine in mg/dL})^{-1.154} \times (\text{age})^{-0.203} \times (0.742 \text{ if female}) \times k$$

The k factor depends on the patient’s race or ethnicity. It is 1 for all patients except those of African or Caribbean descent, for whom k = 1.22.

6) Bortezomib safety: adverse events and infections recorded at each visit during the bortezomib treatment phase and during the follow-up phase for up to one year.

7) Patient survival and graft survival at 1 year.

8) Changes in peripheral blood T- and B-cell subpopulations during bortezomib treatment, assessed by flow cytometry at inclusion, 6 months, and 12 months.

7. SAFETY AND TOLERABILITY EVALUATION

7.1. Description of safety evaluation parameters

The safety of bortezomib will be evaluated using the three main criteria for bortezomib toxicity, together with the occurrence of infectious adverse events.

1) Neurotoxicity: This will be assessed using the neuropathy scale described in Section 5.2. A baseline

neurological examination will be performed on the day of enrollment and repeated before and after each bortezomib cycle. If neurotoxicity occurs during the first cycle and is present at the start of the second cycle, the Velcade dose will be reduced or the scheduled injection canceled, depending on the severity.

2) Hepatic toxicity: This will be assessed by monitoring several liver function tests, including bilirubin and transaminase levels. A bortezomib dose adjustment schedule is provided in Chapter 5.2 and will be implemented according to the severity of abnormalities in liver function tests.

3) Hematologic toxicity: This will be assessed by a complete blood count performed at each bortezomib injection. A bortezomib dose adjustment schedule is provided in Chapter 5.2 based on the severity of abnormalities in white blood cell and platelet counts.

4) Monitoring for infections: The final safety parameter to monitor during bortezomib treatment is the occurrence of infections. Infections requiring hospitalization must be reported as an adverse event. Infectious episodes requiring hospitalization must be reported as SAEs.

7.2. Planned methods and schedule for the assessment, collection and analysis of safety endpoints

1) A baseline neurological examination will be performed on the day of study inclusion, before each bortezomib treatment cycle (D14 and Day 35), and after each treatment cycle (W8 and W11). Neurotoxicity assessments will be recorded in the eCRF on each of these dates. The occurrence of grade 3 or 4 neurotoxicity will require the discontinuation of bortezomib and will be reported as a serious adverse event.

2) Liver function tests will be monitored and systematically recorded in the eCRF on D14, D35, W8, W11, M6, M9, and M12. In addition, during the two bortezomib treatment cycles, liver function tests will be performed on each day of bortezomib administration (four injections during the first cycle and four injections during the second cycle) and recorded in the eCRF if abnormalities are detected. In the event of severe liver function abnormalities, treatment must be discontinued, and this will be reported as a serious adverse event.

3) Complete blood counts will be monitored and systematically recorded in the eCRF on D14, D35, W8, W11, M6, M9, and M12. In addition, during the two bortezomib treatment cycles, complete blood counts will be performed on each day of bortezomib administration (four injections during the first cycle and four injections during the second cycle) and recorded in the eCRF if abnormalities are detected. In the event of severe abnormalities, treatment must be discontinued, and this will be reported as a serious adverse event.

4) SAEs of infectious origin must be reported by the investigator without delay, from the day on which the investigator becomes aware of them.

7.3. Procedures implemented for the recording and reporting of adverse events

7.3.1 Definitions

According to Article R1123-39 of the French Public Health Code:

- **Adverse event**

  Any harmful occurrence in a person participating in biomedical research, whether or not the occurrence is related to the research or to the investigational product.

- **Adverse effect**

Any harmful and unintended response to an investigational medicinal product, regardless of the dose administered.

- **Serious adverse event or serious adverse reaction**

Any adverse event or adverse reaction that:

- results in death;
- is life-threatening;
- requires hospitalization or prolongation of existing hospitalization;
- results in significant or persistent disability or incapacity;
- results in a congenital anomaly or birth defect;
- and, in relation to a medicinal product, occurs regardless of the dose administered.

- **Unexpected adverse reaction to an investigational medicinal product**

Any adverse reaction whose nature, severity, or outcome is not consistent with the information provided in the Summary of Product Characteristics when the medicinal product is authorized, or in the Investigator's Brochure when the medicinal product is not authorized.

- **New safety information**

Any new safety data that may lead to a reassessment of the benefit-risk balance of the study or the investigational medicinal product, or that may be sufficient to warrant consideration of modifications to the administration of the investigational medicinal product or to the conduct of the study.

For example:

- A serious adverse event (SAE) that may be related to the investigations and diagnostic procedures performed as part of the trial and that could affect the conduct of the trial;
- A significant risk to the trial population, such as a lack of efficacy of the investigational medicinal product used for the treatment of a life-threatening disease;
- Significant findings arising from a recently completed animal study (such as a carcinogenicity study);
- An early termination or temporary interruption for safety reasons of a trial conducted with the same investigational medicinal product in another country;
- An unexpected serious adverse reaction related to a non-investigational medicinal product required for the conduct of the trial (e.g. challenge agents, rescue treatment).

7.3.2. Severity grade

The severity of AEs is assessed by the Investigator using the Common Terminology Criteria for Adverse Events, version 4.0.
Note: The severity grade should not be confused with the seriousness criterion.

7.3.3. Role of the Investigator

7.3.3.1. Regulatory Obligations of the Investigator (Article R.1123-54)

The investigator must notify the sponsor, **without delay from the day on which they become aware of them**, of all serious adverse events, except those identified in the protocol (see protocol-specific sections) or in the Investigator's Brochure as not requiring immediate notification.

The investigator must therefore report all events meeting any of the seriousness criteria listed below, except those identified in the protocol as not requiring notification:

1- Death

2- Life-threatening event

3- Requires or prolongs

hospitalization 4 - persistent sequelae

5- Congenital anomaly or birth defect

Any other event considered "medically significant"

7.3.3.2. Protocol-specific requirements:

7.3.3.2.1. Events and effects NOT REQUIRING immediate reporting by the investigator

The events listed below do not require immediate notification by the investigator but may be recorded in the "adverse event" section of the case report form:

● Planned hospitalization unrelated to the condition under study (e.g. annual diabetes follow-up, kidney transplant assessment);

● Hospitalization for routine treatment or monitoring of the condition under study, not associated with deterioration of the patient's condition.

The following expected non-serious adverse effects do not require immediate notification by the investigator:

a) For expected non-serious adverse effects related to the investigational medicinal product: • Refer to the SmPC for bortezomib shown in Appendix 4.

b) For adverse effects related to other medicinal products used during the research:

• **Plasma exchanges**

The adverse effects most frequently encountered during plasma exchanges depend on the technique used, the replacement solution and the vascular access route.

1) Metabolic adverse effects

The main risk is symptomatic hypocalcemia related to the use of citrate as an anticoagulant. The main symptoms are:

- Presence of plasma
- Paresthesia
- Muscle cramps
- Rarely, secondary cardiac arrhythmia

These effects can be prevented or corrected by administering intravenous calcium during plasma exchange.

2) Hemorrhagic risk

This risk is related to the removal of coagulation factors and their failure to be replaced when albumin is used as a replacement. This risk can be prevented by measuring fibrinogen levels before each exchange and administering human fibrinogen in the event of hypofibrinogenemia. Replacement with human plasma alone or in combination with albumin reduces the hemorrhagic risk, particularly when exchanges are performed close to an invasive procedure such as graft biopsy.

3) Infectious complications:

The risk is related to the removal of immunoglobulins. However, this risk is offset in the Tribute protocol by the infusion of large quantities of polyclonal immunoglobulins. In addition, there is a very low but not zero risk of viral transmission when replacement is performed with fresh frozen plasma, despite the viral inactivation procedures to which these products are subjected.

Finally, there is an infectious risk associated with the placement of a central venous catheter if exchanges cannot be performed on an arteriovenous fistula or peripheral vein. This risk will nevertheless be limited by the short duration of catheter maintenance required by the protocol (as only 5 exchanges are required) and by catheter tunnelling.

- **Prednisone or prednisolone**

► Cardiovascular disorders: fluid and sodium retention, arterial hypertension.

► Endocrine and metabolic disorders: iatrogenic Cushing's syndrome, reduced ACTH secretion, adrenal cortical atrophy (occasionally permanent), corticosteroid-induced diabetes.

► Musculoskeletal disorders: muscle atrophy preceded by muscle weakness (due to increased protein catabolism), osteoporosis, pathological fractures particularly vertebral compression fractures, aseptic osteonecrosis of the femoral heads. A few cases of tendon rupture have been reported in exceptional circumstances, particularly with concomitant fluoroquinolone treatment.

► Gastrointestinal disorders: gastroduodenal ulcers, small bowel ulcerations, gastrointestinal perforations and hemorrhage.

► Skin disorders: skin atrophy, acne, purpura, ecchymosis, hypertrichosis, delayed wound healing.

► Neuropsychiatric disorders: euphoria, insomnia, agitation.

► Eye disorders: certain forms of glaucoma and cataracts.

- **Tacrolimus**

The most common adverse effects specifically associated with tacrolimus are listed below:

► Infections: as with other potent immunosuppressive agents, patients receiving tacrolimus frequently have an increased risk of infections (viral, bacterial, fungal and protozoal). Cases of BK virus nephropathy, which may impair graft function, as well as cases of JC virus-associated progressive multifocal leukoencephalopathy (PML), have been reported.

► Tumors: patients receiving any immunosuppressive treatment have an increased risk of developing malignant tumors.

► Metabolism disorders: hyperglycemia, diabetes mellitus, hyperlipidemia, hypercholesterolemia, hypertriglyceridemia, hyperkalemia, metabolic acidosis.

► Psychiatric disorders: insomnia, anxiety, confusion and disorientation, depression, depressed mood, mood disorders, nightmares, hallucinations and mental disorders.

► Neurological disorders: tremor, headaches, seizures, disturbances of consciousness, paresthesia and dysesthesia, peripheral neuropathies.

► Very frequent gastrointestinal disorders: diarrhea, nausea, stomatitis and ulceration, gastrointestinal and abdominal pain, dyspeptic signs and symptoms, constipation, flatulence, meteorism and bloating, loose stools.

► Hepatobiliary disorders: cholestasis and jaundice, hepatocellular lesions and hepatitis, cholangitis.

► Skin and subcutaneous tissue disorders: pruritus, rash, alopecia, acne, hyperhidrosis.

► Musculoskeletal and systemic disorders: arthralgia, muscle spasms, pain in the limbs, back pain.

► Kidney disorders: impaired kidney function.

- **Mycophenolic acid (Mycophenolate mofetil / Mycophenolate Sodium)**

The most common adverse events that may be specifically attributed to mycophenolate are listed below.

► Infections: As with other potent immunosuppressive agents, patients receiving mycophenolate are at increased risk of infections (viral, bacterial, fungal and protozoal). Cases of BK virus nephropathy, which may impair graft function, as well as cases of JC virus-associated progressive multifocal leukoencephalopathy (PML), have been reported.
► Tumors: patients receiving any immunosuppressive treatment have an increased risk of developing malignant tumors.
► Hematologic disorders: Leukopenia, neutropenia, lymphopenia, anemia, thrombocytopenia.
► Respiratory disorders: Dyspnea, cough, pneumopathy, pleural effusion.
► Gastrointestinal disorders: Vomiting, abdominal pain, diarrhea, nausea. Less frequently: Gastrointestinal hemorrhage, peritonitis, ileus, colitis, gastric ulcer, duodenal ulcer, gastritis, esophagitis, stomatitis, constipation, dyspepsia, flatulence, belching.
► Hepatobiliary disorders: Hepatitis, jaundice, hyperbilirubinemia.

7.3.3.2.2. Events and effects requiring immediate reporting by the investigator

The following expected serious adverse events require immediate reporting by the investigator:

Any infection requiring hospitalization, hematologic disorder, abnormal liver function tests or neuropathy must be reported immediately by the investigator as an adverse event.

For expected serious adverse events related to the investigational medicinal product(s)
-refer to Section 1.5.3 of the protocol and to the SmPC for bortezomib provided in Appendix 4.

7.3.3.3. Procedures for reporting to the sponsor by the investigator

General:

The initial report must be submitted in writing and signed by the investigator using the SAE reporting form provided for this purpose (see Appendix 7).
This initial report must be followed within a maximum of 8 days by one or more detailed written and signed follow-up reports in the case of a fatal or life-threatening event and within 15 days in all other cases.
Each SAE must be described on the SAE reporting form as comprehensively as possible. Every section of the form must be completed by the investigator to allow the sponsor to perform an appropriate assessment.

The initial report, follow-up reports and any relevant supporting documentation, e.g. hospital discharge summary, must be sent exclusively by fax to the Vigilance Unit of the Clinical Research and Innovation Department (CRID) at **+33 (0)1 44 84 17 99**.

For studies using an electronic case report form (eCRF), the investigator must complete the SAE reporting form in the eCRF, validate it, print it, sign it and fax it to **+33 (0)1 44 84 17 99**.
Only if access to the eCRF is unavailable, the investigator must complete and sign the SAE reporting form provided in Appendix 7 and fax it to the sponsor at **+33 (0)1 44 84 17 99**. Once access to the eCRF has been restored, the investigator must complete the SAE reporting form in the eCRF to reconcile the records.

Information to be reported to the sponsor:

The following information must be reported to the sponsor using the SAE reporting form:

- patient identification
- reporter identification
- seriousness and severity of the adverse event,

- date of onset and resolution of the SAE,
- a detailed description of the SAE (including diagnosis, symptoms, chronology, actions taken, outcomes and clinical course),
- the patient's current medical conditions or previous medical history,
- treatments received by the patient (investigational medicinal product(s) and concomitant medication(s))
- and the causal relationship between the SAE and the investigational medicinal product(s), comparator treatment(s), any concomitant medication(s), study procedures or study-specific examinations.

If the investigator considers that the serious adverse event is related to the study or to the investigational medicinal product(s), all documentation that may be useful to the sponsor should be provided whenever possible

(e.g. medical reports, laboratory results and results of additional diagnostic investigations). These documents must be anonymized. They must also include the following information: study acronym, patient number and initials, the nature of the serious adverse event and the date of its onset. The investigator must respond to any request from the sponsor for additional information.

For any questions regarding the reporting of an adverse event, the Vigilance Unit of the CRID may be contacted by email at vigilance.drc@aphp.fr

7.3.3.4. Reporting period to the sponsor

For each study participant, the investigator is required to report SAEs
from the date informed consent is signed until the end of the participant's involvement in the study.

If treatment is discontinued prematurely due to toxicity, a serious adverse event report must be submitted to the sponsor. The serious adverse event will be followed by both the investigator and the sponsor until resolution.

All adverse events will be followed until complete resolution (stabilization at a level considered acceptable by the investigator or return to baseline), even if the participant has withdrawn from the study.

7.3.4. Sponsor responsibilities

7.3.4.1. Assessment and reporting of serious adverse events

The sponsor will continuously evaluate the safety of each investigational medicinal product throughout the study.

The sponsor will assess the seriousness of all adverse events reported by investigators, their causal relationship to each investigational medicinal product and any other concomitant treatments, as well as whether the adverse events are expected or unexpected.

All adverse events for which the investigator and/or the sponsor considers that a causal relationship with the investigational medicinal product can reasonably be suspected will be considered suspected adverse reactions.

Any suspected unexpected serious adverse reaction (SUSAR) or any significant new safety information will be reported by the sponsor to the French National Agency for the Safety of Medicines and Health Products (ANSM) and the appropriate Research Ethics Committee (Comité de Protection des Personnes).

The initial report must be submitted within 7 calendar days of receipt of the serious adverse event in the case of death or a life-threatening event.

The initial report must be submitted within 15 calendar days of receipt of the serious adverse event for all other serious events.

Follow-up information must be submitted within 8 calendar days following the 7- or 15-day reporting period, as appropriate.

All SUSARs will be reported electronically through the European EudraVigilance database for suspected adverse reactions maintained by the European Medicines Agency (EMA).

The sponsor will inform all participating investigators of any information that may adversely affect the safety of study participants.

7.3.4.2. Assessment and reporting of other safety information

This includes any safety data or significant new information that may substantially affect the assessment of the benefit-risk balance of an investigational medicinal product or the study, or that may require changes to the administration of the investigational medicinal product or the conduct of the study.
Significant new safety information must be reported within 15 calendar days of the sponsor becoming aware of the information. Any relevant follow-up information must be submitted within a further 8 calendar days after the 15-day reporting period.

7.3.5. In utero exposure

If a participant becomes pregnant during the study, or following participation of her male partner in the study (where the investigational medicinal product may be present in seminal fluid), the pregnancy must be reported to the sponsor without delay.
The investigator must fax the "Pregnancy follow-up form for pregnancy occurring during a biomedical research study" (Appendix 8) to the Vigilance Unit at **+33 (0)1 44 84 17 99**. If the pregnancy continues, the form must include the expected delivery date, the contact details of the obstetrician and the planned maternity hospital.
The investigator must follow the participant until completion or termination of the pregnancy and report the outcome to the sponsor using the same form. If the pregnancy outcome meets the definition of a serious adverse event (e.g. spontaneous abortion requiring hospitalization, fetal death or congenital anomaly), the investigator must follow the SAE reporting procedure.
In the event of paternal exposure, the investigator must obtain the pregnant partner's consent before collecting information regarding the pregnancy.

7.3.6. Exposure during breastfeeding

Exposure during breastfeeding occurs when an infant or child may have been exposed to a medicinal product through breastfeeding by a mother receiving an investigational medicinal product.
Even if it is not associated with an adverse event, exposure during breastfeeding must always be reported by the investigator to the sponsor as soon as it becomes known.

7.3.7. Independent data monitoring committee

7.3.7.1. General

An Independent Data Monitoring Committee (IDMC) may be established by the sponsor. Its primary responsibility is to be a committee for monitoring safety data. It may also have additional responsibilities, such as monitoring efficacy data, particularly if the protocol involves interim analyses.
The IDMC is referenced in Article L.1123-7 of the French Public Health Code.

The sponsor is responsible for justifying to the competent authority (ANSM) and the Research Ethics Committee (REC) whether an IDMC has or has not been established.

In practice, an IDMC is systematically established for studies in which the additional risk related to the research is classified as Category D. It may also be established for certain studies classified as Category C.

The IDMC provides recommendations to the sponsor regarding continuation, modification or termination of the study. Recommendations issued by the IDMC may include:

- continuation of the study without modification
- continuation of the study with modification of the protocol and/or participant monitoring
- temporary suspension of enrollment
- permanent termination of the study based on:
  - safety data, including serious adverse events,
  - efficacy data, including futility or demonstrated efficacy.

The IDMC is appointed by the sponsor and consists of at least three individuals who are independent of the study, including at least one clinician specializing in the disease under investigation and one specialist in the investigational medicinal product, or a clinical pharmacologist/pharmacovigilance expert. A methodologist/biostatistician may also be included, particularly if interim analyses are planned.

The IDMC serves in an advisory capacity when consulted by the sponsor on safety-related matters, such as safety and reassessment of the benefit-risk profile during the study.

A preliminary IDMC meeting must be held before the first participant is enrolled and ideally before submission of the protocol to the Competent Authority and the REC. The agenda should include, at a minimum:

Definition of the IDMC responsibilities:

- Validation of the study methodology:

The methodology proposed for the clinical trial should be reviewed and validated by the IDMC to ensure that it does not compromise participant safety, particularly with respect to enrollment and randomization procedures.

- Validation of safety follow-up procedures:
  - type of parameters to be assessed
  - frequency of assessments, visit schedule
- Validation of stopping criteria:
  - criteria for discontinuing an individual participant due to safety concerns
  - criteria for temporary or permanent study termination leading to the implementation of predefined recommendations (stopping rules)
- Protocol modifications and recommendations. Based on its review of the study safety data, the IDMC may, as appropriate:
  - recommend substantial protocol amendments to modify specific aspects of the protocol, including eligibility criteria, participant monitoring or additional assessments. The IDMC may also issue any recommendations it considers necessary to ensure participant safety and maintain a favorable benefit-risk profile throughout the study.

Definition of the IDMC operating procedures:

- meeting procedures (open session followed by closed session) and meeting frequency
- procedures and preferred format for transmission of SAEs from the sponsor to the IDMC

At its first meeting, the IDMC will elect its Chair.

The sponsor remains responsible for all final decisions. Where applicable, the sponsor will provide its reasoned decision, together with the IDMC meeting minutes, to the competent authority (ANSM) and the REC.

7.3.7.2. Protocol-specific requirements

An IDMC is planned for this study. The members of the IDMC will be appointed after the study begins.

All the responsibilities and detailed operating procedures of the IDMC will be set out in the study IDMC Charter. At the first IDMC meeting, a Chair will be elected and the members will establish the committee's operating procedures and meeting schedule.

7.4. Criteria for permanent or temporary discontinuation of bortezomib

7.4.1. Temporary treatment discontinuation

Bortezomib treatment must be temporarily interrupted in the circumstances below (see Section 5.2):

- **neutropenia** of between 500 and 1,000/mm³ until neutrophil recovery to >1,000/mm³
- **thrombocytopenia** of between 50,000 and 100,000/mm³ until platelet recovery to >100,000/mm³
- **liver function abnormalities**, including total bilirubin and/or transaminase levels >3 × the upper limit of normal, until normalization
- **neuropathy** (grade 2 with pain or grade 3) until symptoms improve.

7.4.2. Permanent treatment discontinuation

Treatment will be permanently discontinued in the event of:

- Withdrawal of participant consent
- Medical decision:
  - either due to intolerance to the investigational medicinal product (neutrophil count <500/mm³, platelet count <50,000/mm³, grade 4 neuropathy),
  - or due to initiation of kidney replacement therapy (hemodialysis or peritoneal dialysis)
- Temporary or permanent discontinuation of part or all of the study, regardless of the reason.

In the event of premature or temporary discontinuation of study treatment, the investigator must document the reason(s).

7.4.3. Collection methods and schedule for these data

Any participant who permanently or temporarily discontinues study treatment will continue to be followed through the end of the study in accordance with the protocol. In all cases, the Month 12 biopsy will be offered to the participant.

Available data will be included in the statistical analysis unless the participant objects following withdrawal of consent.

These data will be recorded in the case report form during protocol-specified study visits.

7.4.4. Expected adverse reactions associated with bortezomib

See the Summary of Product Characteristics in Appendix 4.

Neuropathy: see the neuropathy monitoring score.

7.4.5. Management of worsening kidney function in the control group

In the control group, participants with rapidly worsening kidney function, defined as an increase in serum creatinine of more than 20% between two assessments performed 3 months apart, may undergo a kidney allograft biopsy. In the

event of an acute worsening of antibody-mediated rejection lesions, treatment – the choice of which will be left to the medical team at the patient's center – may be offered to the patient.

7.5. Criteria for temporary or permanent discontinuation of plasma exchange and intravenous immunoglobulins

In both treatment arms, participants will receive a series of 5 plasma exchange sessions and 4 courses of intravenous immunoglobulins.

7.5.1. Criteria for discontinuation of plasma exchange

A plasma exchange session may be discontinued because of poor tolerability, generally due to an allergic reaction to plasma. Plasma exchange may be performed using 4% or 5% albumin alone for the treatment of antibody-mediated rejection. Lack of functional vascular access, either peripheral or central depending on the plasma exchange technique used, may require permanent discontinuation of plasma exchange treatment.

7.5.2. Criteria for discontinuation of intravenous immunoglobulins

If acute kidney injury develops during or following an intravenous immunoglobulin infusion administered at the recommended rate (2g/kg over at least 48 hours), together with kidney allograft biopsy findings consistent with tubular toxicity, intravenous immunoglobulin treatment will be permanently discontinued.

7.6. Study withdrawal

Participants may discontinue their participation in the study at any time for any reason, or at the investigator's decision.

7.6.1. Criteria for discontinuation of study participation

- Study participation may be discontinued because of poor protocol adherence or loss to follow-up,
- withdrawal of participant consent,
- or death.

7.6.2. Follow-up procedures after study withdrawal

All study withdrawals must be documented, and the investigator must specify the reason. The reason will be recorded in the case report form as one of the following:

- adverse event,
- loss to follow-up*,
- Repeated protocol violations,
- or administrative reasons.

After study withdrawal, participants may resume routine management of their condition at their usual treatment center if it is not the study assessment center. Participant information will be transferred to the physician of their choice. In the event of a serious adverse event, the participant must be monitored until complete clinical and laboratory recovery, or until the condition has stabilized, including after study withdrawal if necessary.

***Loss to follow-up**

It is considered unlikely that participants included in this study will be lost to follow-up. The rate of loss to follow-up is typically very low (<5%) in cohorts of kidney transplant recipients. Participants included in this study are already receiving regular follow-up care, have agreed to undergo a kidney allograft biopsy and therefore understand the risk of their allograft. By selection, this study population is expected to have relatively good treatment adherence. Once the initial treatment has been administered in the experimental arm, it is considered unlikely that participants will fail to attend the routine follow-up visits required by the study.

Nevertheless, for participants considered lost to follow-up, the case report form must be completed through the last completed study visit. The investigator will make every effort to contact the participant to determine the reason for study withdrawal and the participant's health status. Documentation of all contact attempts, including letters and dates of telephone calls, must be maintained.

Participants who withdraw from the study will not be re-enrolled or replaced. They will continue to be followed at their treating center outside the study protocol.

7.7. Criteria for early termination of part or all of the study

The study may be terminated if a significant increase in serious adverse events is observed in the bortezomib group compared with the control group.

8. DATA MANAGEMENT AND STATISTICAL CONSIDERATIONS

8.1. Sample size

The percentage of participants achieving treatment success at 1 year, defined as a reduction of more than 50% in DSA titer(s) together with stabilization or improvement of histologic lesions, is estimated to be 5% in the control group. The required sample size was calculated to demonstrate a 20% increase in the percentage of participants achieving treatment success with bortezomib (i.e. 25% in the experimental group). Assuming a type I error rate of 5% and 80% power, 50 participants per treatment group, for a total of 100 participants, are required.

The target enrollment of 50 patients per group can be achieved during the 2-year recruitment period. The participating transplant centers perform between 100 and 150 kidney transplants annually, corresponding to approximately 1,000 to 1,500 kidney transplants per year across the 10 participating centers. Approximately one-third of transplant recipients are pre-sensitized and therefore approximately 800 patients per year may be at risk of developing de novo DSA. Based on the literature reviewed in Section 2.1.3.2, a conservative estimate of the annual incidence of de novo DSA is 7% to 8%, corresponding to approximately 60 patients per year developing dnDSA, assuming 800 kidney transplants are performed annually across the 10 participating centers. No upper limit on the time elapsed since transplantation has been established for study inclusion. Assuming, for convenience, a stable incidence of de novo DSA, it is estimated that 30 patients could be enrolled among those transplanted between 6 months and 1 year, 60 patients among those transplanted between 1 and 2 years, 60 patients between 2 and 3 years, 60 patients between 3 and 4 years and so forth. Regarding the frequency of histologic lesions, an internal survey was conducted across 6 centers to determine the frequency of chronic active antibody-mediated rejection diagnosed on kidney allograft biopsies during 2009-2010, corresponding to the planned 2-year enrollment period of the PHRC study. Across these 6 centers, 95 biopsies met the

histologic inclusion criteria for chronic active AMR, with a g+ptc score of >2 and a cg score of <3. These biopsies were performed because of graft dysfunction or proteinuria. In addition, the protocol provides for enrollment of patients upon the development of de novo DSA before the onset of clinical signs, which should further increase recruitment capacity.

8.2. Statistical analysis strategy

Statistical analyses will be performed by Dr. Caroline Elie (Clinical Research Unit, Paris Centre, Hôpital Necker) using the R statistical software (http://cran.r-project.org/).
Analyses of the primary endpoint and all secondary endpoints will be conducted according to the intention-to-treat (ITT) principle. All statistical tests will be two-sided with a significance level of 5%.

A descriptive analysis of baseline clinical and biologic characteristics will first be performed for each treatment group. Quantitative data will be summarized as mean ± standard deviation or median with range, and qualitative data will be expressed as frequencies and percentages.

8.2.1. Analysis of the primary endpoint

The percentage of patients achieving treatment success at 1 year will be compared between the two treatment groups using the chi-square test.

8.2.2. Analysis of the secondary endpoints

For all histologic and immunologic secondary endpoints and safety endpoints, comparisons of the two means will be performed using Student's t-test or, if required, the Wilcoxon rank-sum test. Comparisons of categorical variables will be performed using the chi-square test or, if required, Fisher's exact test.
The mean fluorescence intensity of DSA at 6 months will be compared between the two treatment groups using Student's t-test.
Kidney function will be compared between the treatment groups at each follow-up time point.
Changes in DSA and kidney function over time will be described using methods appropriate for each variable type (absolute values or percentage change per unit time). These longitudinal data may also be analyzed using linear mixed-effects regression models or marginal regression models to account for within-patient correlations.

8.3. Data management

Statistical analysis will be performed by Dr. Caroline Elie (Clinical Research Unit, Paris Centre, Hôpital Necker) using the R statistical software (http://cran.r-project.org/).
Analyses of the primary endpoint and all secondary endpoints will be conducted according to the intention-to-treat (ITT) principle. All statistical tests will be two-sided with a significance level of 5%.

9. RIGHT OF ACCESS TO DATA AND SOURCE DOCUMENTS

9.1. Case report form

All information required by the protocol must be recorded in the case report form, and the investigator must provide an

explanation for each missing data item. Data must be entered into the electronic case report forms as they are obtained, whether clinical or paraclinical. Erroneous data identified in the case report forms will be subject to a data correction query. With the electronic case report form, all modifications will be tracked by the IT system. The anonymity of patients will be ensured by assigning a code number and recording their initials on all documents required for the research, or by appropriately removing identifying information from copies of the source documents intended for the documentation of the research.

Electronic data contained in a file will be declared to the CNIL (France's data protection authority) in accordance with the procedure applicable to the study.

9.2. Identification of data considered to be source data

The data considered to be source data will include the medical record, consultation reports, nursing observation charts, laboratory results, electrocardiograms and their corresponding reports.

The patient monitoring diaries provided to the parents will also be considered source data.

9.3. Right of access to data and source documents

Persons having direct access in accordance with the applicable legislative and regulatory provisions, in particular Articles L.1121-3 and R.5121-13 of the French Public Health Code (including investigators, persons responsible for quality control, monitors, clinical research associates, auditors and all persons involved in the conduct of the trial), shall take all necessary precautions to ensure the confidentiality of information relating to the investigational medicinal products, the clinical trials, the persons participating in them, particularly with regard to their identity, and the results obtained. Data collected by these persons during quality control procedures or audits will be anonymized.

9.4. Data processing and retention of documents and data

Documents relating to research conducted under the French law governing biomedical research must be archived by all parties for a period of 15 years after completion of the research.

(See GCP, Section 8: Essential documents)

The indexed archive shall include:

- Copies of the ANSM authorization letter and the mandatory opinion of the REC
- Successive versions of the protocol (identified by version number and version date)
- Correspondence with the sponsor
- Signed informed consent forms kept in sealed envelopes (for minors, signed by the holders of parental authority), together with the corresponding inclusion list or register.
- The completed and validated case report form for each included subject
- All study-specific appendices
- The final study report resulting from the statistical analysis and quality control of the study (submitted in duplicate to the sponsor).
- Any audit certificates issued during the conduct of the research.

The database used for the statistical analysis must also be archived by the person responsible for the analysis (hard copy or electronic format).

10. QUALITY CONTROL AND QUALITY ASSURANCE

The research will be conducted according to the sponsor's standard operating procedures.

The conduct of the research at the investigational centers and the management of subjects will be performed in accordance with the Declaration of Helsinki and the applicable Good Clinical Practice guidelines.

10.1. Monitoring procedures

The clinical research associates representing the sponsor will perform visits to the investigational centers according to the patient follow-up schedule set out in the protocol, the number of inclusions at the different centers and the level of risk assigned to the research.

- **Opening visit of each center:** before inclusion, to implement the protocol and become familiar with the various persons involved in the biomedical research.

- **During subsequent visits**, the case report forms will be reviewed progressively by the clinical research associates according to the progress of the research. The principal investigator at each center, as well as the other investigators who enroll or ensure the follow-up of participants in the research, undertake to receive the clinical research associates at regular intervals.

During these on-site visits and in accordance with Good Clinical Practice, the following elements will be reviewed:

- Compliance with the protocol and the procedures defined for the research.
- Verification of patients' informed consent forms.
- Review of source documents and comparison with the data reported in the case report forms regarding accuracy, missing data and data consistency. These should be reviewed in accordance with the rules established by the CRID procedures and the monitoring guide established between the coordinating investigator and the clinical research unit.
- Verification of product accountability within the department or pharmacy and through the product accountability records completed by the investigator.

- **Close-out visit:** collection of case report forms, pharmacy review, biomedical research documents and archiving.

10.2. Quality control by an audit team

Any study may be audited by the sponsor at any time. During an auditor visit, the investigator and their team will make themselves available and authorize access to the technical facilities, study equipment and patient records. Patient anonymity must be respected and the information verified during these controls must remain confidential.

10.3. Quality control by health authorities

During a possible inspection by the health authorities, the following points may be reviewed:

- The general organization of the study

- The qualifications of the personnel responsible for its conduct
- The quality of the equipment
- The informed consent forms
- Obtaining the opinion of the CPP
- The procedures for dispensing and storing products
- The conduct of the study
- The archiving of documents relating to the study

In the event of an inspection by the authorities, the investigator shall inform the sponsor as soon as the request has been made.

11. LEGAL AND ETHICAL CONSIDERATIONS

The sponsor is defined by Law 2004-806 of August 9, 2004. In this research, AP-HP is the sponsor and the Clinical Research and Innovation Department (CRID) is responsible for ensuring the regulatory missions.

Before the start of the research, each investigator will provide the sponsor's representative with a copy of their ***dated and signed curriculum vitae****, including their registration number with the Medical Council (Ordre des Médecins) or their ADEL number or RPPS number.*

11.1. Application for authorization from ANSM

In order to start the research, AP-HP, as the sponsor, must submit an application for authorization to the competent authority, ANSM. The competent authority, as defined in Article L.1123-12, issues its decision with regard to the safety of persons participating in biomedical research, taking into consideration, in particular, the safety and quality of products used during the research, in accordance, where applicable, with the applicable standards, their conditions of use and the safety of persons with regard to the procedures performed and methods used, as well as the procedures planned for participant follow-up.

11.2. Application for an opinion from the research ethics committee

In accordance with Article L.1123-6 of the French Public Health Code, the research protocol must be submitted by the sponsor to a Research Ethics Committee (*Comité pour la Protection des Personnes*) for this protocol, the CPP Ile-de-France II). The opinion of this committee is notified to the competent authority by the sponsor before the start of the research.

11.3. Procedures relating to data protection and information technology regulations

The computerized file used for this research will be implemented in accordance with French regulations (the amended Data Protection Act, "Loi Informatique et Libertés") and European regulations (General Data Protection Regulation - GDPR).

This research falls within the scope of the "Reference Methodology for the processing of personal data implemented in the context of research in the field of health" (amended MR-001). AP-HP, as the sponsor of the research, has signed a compliance commitment with this "Reference Methodology".

11.4. Amendments

The CRID must be informed of any proposed modification to the protocol by the coordinating investigator. Modifications must be classified as substantial or non-substantial.

A substantial modification is a modification that may, in one way or another, alter the guarantees provided to persons participating in biomedical research (modification of an inclusion criterion, extension of the inclusion period, participation of new centers, etc.).

After the start of the research, any substantial modification initiated by the sponsor must obtain, prior to implementation, a favorable opinion from the committee and authorization from the competent authority. In this case, if necessary, the committee ensures that new consent from persons participating in the research is obtained.

Furthermore, any extension of the research (major modification of the therapeutic scheme or of the populations included, extension of treatments and/or therapeutic procedures not initially planned in the protocol) must be considered as a new research study.

Any substantial modification must be the subject of an application for authorization to ANSM and/or an application for an opinion from the REC **by the sponsor**.

11.5. Information sheet and informed consent

Written consent must be obtained from every person participating in the research before performing any procedure required by the biomedical research.

After verification of the inclusion and exclusion criteria during the prescreening phase, the investigator will provide patients with detailed information regarding, in particular, the purpose, potential benefits, risks, and study-related requirements of the research for which they are being invited to participate.

After signing the informed consent form, the patient will undergo the examinations required to confirm inclusion in the study. **The patient will be considered included in the trial on the date of randomization.**

11.6. Final research report and conclusions

The final research report will be written jointly by the coordinator and the biostatistician for this research. This report will be submitted to each investigator for review. Once consensus has been reached, the final version must be approved by the signature of each investigator and submitted to the sponsor as soon as possible after the actual completion of the research. A report prepared according to the reference format of the competent authority must be submitted to the competent authority and to the REC within one year after the end of the research, defined as the last follow-up visit of the last subject included. This period is reduced to 90 days in the event of premature termination of the research.

12. INSURANCE AND SCIENTIFIC COMMITMENT

12.1. Insurance

Assistance Publique–Hôpitaux de Paris (AP-HP) is the sponsor of this research. In accordance with the law on biomedical research, it has taken out insurance with the company HDI-Gerling for the entire duration of the research,

covering its own civil liability as well as that of all persons involved (physicians or staff involved in the conduct of the research) (Law No. 2004-806, Article L.1121-10 of the French Public Health Code).
Assistance Publique–Hôpitaux de Paris reserves the right to interrupt the research at any time for medical or administrative reasons; in such an event, notification will be provided to the investigator.

12.2. Scientific commitment

Each investigator undertakes to comply with the obligations of the law and to conduct the research in accordance with GCPs, while respecting the terms of the current Declaration of Helsinki. For this purpose, a copy of the **scientific commitment (CRID template document)**, dated and signed **by each investigator** from each clinical department of a participating center, will be provided to the sponsor's representative.

13. PUBLICATION RULES

AP-HP is the owner of the data and no use or transfer to a third party may be made without its prior approval.
The first authors of publications will be the persons who have actually participated in the development of the protocol and its conduct, as well as in the drafting of the results.
Assistance Publique–Hôpitaux de Paris must be mentioned as the sponsor of the biomedical research and, where applicable, as financial support. The terms "Assistance Publique–Hôpitaux de Paris" must appear in the authors' affiliations. The Clinical Research Unit Necker-Cochin must be mentioned in the article.

14. LIST OF APPENDICES

APPENDIX 1: Bibliography

APPENDIX 2: Number of investigational centers

APPENDIX 3 : Bush Classification 2013

APPENDIX 4 : Bortezomib (Velcade®) SmPC

APPENDIX 5 : Table of medication neurotoxicity grades

APPENDIX 6 : Other medications used in the study

APPENDIX 7 : SAE reporting form to the sponsor

APPENDIX 8: Follow-up form for a pregnancy occurring during biomedical research

APPENDIX 9 : Ancillary tests

APPENDIX 1: Bibliography

Anglicheau D, Loupy A, Suberbielle C, Zuber J, Patey N, Noel LH et al. Posttransplant prophylactic intravenous immunoglobulin in kidney transplant patients at high immunological risk: a pilot study. Am J Transplant **2007**;7(5):1185-1192.

Ashton-Chess J, Mai HL, Jovanovic V, Renaudin K, Foucher Y, Giral M et al. Immunoproteasome beta subunit 10 is increased in chronic antibody-mediated rejection. Kidney Int **2010**;77(10):880-890.

Billing H, Rieger S, Ovens J, Susal C, Melk A, Waldherr R et al. Successful treatment of chronic antibody-mediated rejection with IVIG and rituximab in pediatric renal transplant recipients. Transplantation **2008**;86(9):1214-1221.

Cascio P, Oliva L, Cerruti F, Mariani E, Pasqualetto E, Cenci S et al. Dampening Ab responses using proteasome inhibitors following in vivo B cell activation. Eur J Immunol **2008**;38(3):658-667.

Colvin RB. Antibody-mediated renal allograft rejection: diagnosis and pathogenesis. J Am Soc Nephrol **2007**;18(4):1046-1056.

Colvin RB, Hirohashi T, Farris AB, Minnei F, Collins AB, Smith RN. Emerging role of B cells in chronic allograft dysfunction. Kidney Int **2010**;78 Suppl 119:S13-17.

Einecke G, Sis B, Reeve J, Mengel M, Campbell PM, Hidalgo LG et al. Antibody-mediated microcirculation injury is the major cause of late kidney transplant failure. Am J Transplant **2009**;9(11):2520-2531.

El-Zoghby ZM, Stegall MD, Lager DJ, Kremers WK, Amer H, Gloor JM et al. Identifying specific causes of kidney allograft loss. Am J Transplant **2009**;9(3):527-535.

Everly JJ, Walsh RC, Alloway RR, Woodle ES. Proteasome inhibition for antibody-mediated rejection. Curr Opin Organ Transplant **2009**;14(6):662-666.

Everly MJ, Everly JJ, Susskind B, Brailey P, Arend LJ, Alloway RR et al. Bortezomib provides effective therapy for antibody- and cell-mediated acute rejection. Transplantation **2008**;86(12):1754-1761.

Fehr T, Rusi B, Fischer A, Hopfer H, Wuthrich RP, Gaspert A. Rituximab and intravenous immunoglobulin treatment of chronic antibody-mediated kidney allograft rejection. Transplantation **2009**;87(12):1837-1841.

Flechner SM, Fatica R, Askar M, Stephany BR, Poggio E, Koo A et al. The role of proteasome inhibition with bortezomib in the treatment of antibody-mediated rejection after kidney-only or kidney-combined organ transplantation. Transplantation **2010**;90(12):1486-1492.

Furness PN, Philpott CM, Chorbadjian MT, Nicholson ML, Bosmans JL, Corthouts BL et al. Protocol biopsy of the stable renal transplant: a multicenter study of methods and complication rates. Transplantation **2003**;76(6):969-973.

Gaston RS, Cecka JM, Kasiske BL, Fieberg AM, Leduc R, Cosio FC et al. Evidence for antibody-mediated injury as a major determinant of late kidney allograft failure. Transplantation **2010**;90(1):68-74.

Gertz MA, Zeldenrust SR. Treatment of immunoglobulin light chain amyloidosis. Curr Hematol Malig Rep **2009**;4(2):91-98.

Gill JS, Landsberg D, Johnston O, Shapiro RJ, Magil AB, Wu V et al. Screening for de novo anti-human leukocyte antigen antibodies in nonsensitized kidney transplant recipients does not predict acute rejection. Transplantation **2010**;89(2):178-184.

Han M, Rogers JA, Lavingia B, Stastny P. Peripheral blood B cells producing donor-specific HLA antibodies in vitro. Hum Immunol **2009**;70(1):29-34.

Hidalgo LG, Campbell PM, Sis B, Einecke G, Mengel M, Chang J et al. De novo donor-specific antibody at the time of kidney transplant biopsy associates with microvascular pathology and late graft failure. Am J Transplant **2009**;9(11):2532-2541.

Hourmant M, Cesbron-Gautier A, Terasaki PI, Mizutani K, Moreau A, Meurette A et al. Frequency and clinical implications of development of donor-specific and non-donor-specific HLA antibodies after kidney transplantation. J Am Soc Nephrol **2005**;16(9):2804-2812.

Jagannath S, Richardson PG, Barlogie B, Berenson JR, Singhal S, Irwin D et al. Bortezomib in combination with

dexamethasone for the treatment of patients with relapsed and/or refractory multiple myeloma with less than optimal response to bortezomib alone. Haematologica **2006**;91(7):929-934.

Jordan SC, Toyoda M, Vo AA. Intravenous immunoglobulin a natural regulator of immunity and inflammation. Transplantation **2009**;88(1):1-6.

Kamar N, Milioto O, Puissant-Lubrano B, Esposito L, Pierre MC, Mohamed AO, et al. Incidence and predictive factors for infectious disease after rituximab therapy in kidney-transplant patients. Am. J. Transplant. 2010;10(1):89-98.

Kastritis E, Anagnostopoulos A, Roussou M, Toumanidis S, Pamboukas C, Migkou M et al. Treatment of light chain (AL) amyloidosis with the combination of bortezomib and dexamethasone. Haematologica **2007**;92(10):1351-1358.

Lachmann N, Terasaki PI, Budde K, Liefeldt L, Kahl A, Reinke P et al. Anti-human leukocyte antigen and donorspecific antibodies detected by luminex posttransplant serve as biomarkers for chronic rejection of renal allografts. Transplantation **2009**;87(10):1505-1513.

Lefaucheur C, Nochy D, Hill GS, Suberbielle-Boissel C, Antoine C, Charron D, et al. Determinants of poor graft outcome in patients with antibody-mediated acute rejection. Am. J. Transplant. 2007;7(4):832-41

Lefaucheur C, Nochy D, Andrade J, Verine J, Gautreau C, Charron D et al. Comparison of combination Plasmapheresis/IVIg/anti-CD20 versus high-dose IVIg in the treatment of antibody-mediated rejection. Am J Transplant **2009**;9(5):1099-1107.

Lefaucheur C, Loupy A, Hill GS, Andrade J, Nochy D, Antoine C, et al. Preexisting donor-specific HLA antibodies predict outcome in kidney transplantation. J. Am. Soc. Nephrol. 2010;21(8):1398-406.

Loupy A, Suberbielle-Boissel C, Zuber J, Anglicheau D, Timsit MO, Martinez F et al. Combined posttransplant prophylactic IVIg/anti-CD20/plasmapheresis in kidney recipients with preformed donor-specific antibodies: a pilot study. Transplantation **2010**;89(11):1403-1410.

Loupy A, Hill GS, Suberbielle C, Anglicheau D, Zuber J, Timsit MO et al. Significance of C4d Banff scores in early protocol biopsies of kidney transplant recipients with preformed donor specific antibodies (DSA). Am J Transplant ***in press***

Luo H, Wu Y, Qi S, Wan X, Chen H, Wu J. A proteasome inhibitor effectively prevents mouse heart allograft rejection. Transplantation **2001**;72(2):196-202.

Mao Q, Terasaki PI, Cai J, Briley K, Catrou P, Haisch C et al. Extremely high association between appearance of HLA antibodies and failure of kidney grafts in a five-year longitudinal study. Am J Transplant **2007**;7(4):864-871.

Neubert K, Meister S, Moser K, Weisel F, Maseda D, Amann K et al. The proteasome inhibitor bortezomib depletes plasma cells and protects mice with lupus-like disease from nephritis. Nat Med **2008**;14(7):748-755.

Perry DK, Burns JM, Pollinger HS, Amiot BP, Gloor JM, Gores GJ et al. Proteasome inhibition causes apoptosis of normal human plasma cells preventing alloantibody production. Am J Transplant **2009**;9(1):201-209.

Perry DK, Pollinger HS, Burns JM, Rea D, Ramos E, Platt JL et al. Two novel assays of alloantibody-secreting cells demonstrating resistance to desensitization with IVIG and rATG. Am J Transplant **2008**;8(1):133-143.

Rajkumar SV. Multiple myeloma: 2011 update on diagnosis, risk-stratification, and management. Am J Hematol **2011**;86(1):57-65.

Ramos EJ, Pollinger HS, Stegall MD, Gloor JM, Dogan A, Grande JP. The effect of desensitization protocols on human splenic B-cell populations in vivo. Am J Transplant **2007**;7(2):402-407.

Rostaing L, Guilbeau-Frugier C, Fort M, Mekhlati L, Kamar N. Treatment of symptomatic transplant glomerulopathy with rituximab. Transpl Int **2009**;22(9):906-913.

Sberro-Soussan R, Zuber J, Suberbielle-Boissel C, Candon S, Martinez F, Snanoudj R et al. Bortezomib as the sole post-renal transplantation desensitization agent does not decrease donor-specific anti-HLA antibodies. Am J Transplant **2010**;10(3):681-686.

Schwarz A, Gwinner W, Hiss M, Radermacher J, Mengel M, Haller H. Safety and adequacy of renal transplant protocol biopsies. Am J Transplant **2005**;5(8):1992-1996.

Sis B, Campbell PM, Mueller T, Hunter C, Cockfield SM, Cruz J et al. Transplant glomerulopathy, late antibody-

mediated rejection and the ABCD tetrad in kidney allograft biopsies for cause. Am J Transplant **2007**;7(7):1743- 1752.

Smith RN, Colvin RB. Chronic alloantibody mediated rejection. Semin Immunol. 2011;23(4):244-250.

Snanoudj R, Candon S, Legendre C. Targeting B cells in sensitized kidney transplant patients: state of the art and future perspectives. Curr Opin Organ Transplant. 2010;15(5):581-587.

Solez K, Colvin RB, Racusen LC, Haas M, Sis B, Mengel M et al. Banff 07 classification of renal allograft pathology: updates and future directions. Am J Transplant **2008**;8(4):753-760.

Solez K, Colvin RB, Racusen LC, Sis B, Halloran PF, Birk PE et al. Banff '05 Meeting Report: differential diagnosis of chronic allograft injury and elimination of chronic allograft nephropathy ('CAN'). Am J Transplant **2007**;7(3):518-526.

Terasaki PI, Ozawa M. Predictive value of HLA antibodies and serum creatinine in chronic rejection: results of a 2-year prospective trial. Transplantation **2005**;80(9):1194-1197.

Trivedi HL, Terasaki PI, Feroz A, Everly MJ, Vanikar AV, Shankar V et al. Abrogation of anti-HLA antibodies via proteasome inhibition. Transplantation **2009**;87(10):1555-1561.

Vo AA, Lukovsky M, Toyoda M, Wang J, Reinsmoen NL, Lai CH et al. Rituximab and intravenous immune globulin for desensitization during renal transplantation. N Engl J Med **2008**;359(3):242-251.

Waiser J, Budde K, Schutz M, Liefeldt L, Rudolph B, Schonemann C, et al. Comparison between bortezomib and rituximab in the treatment of antibody-mediated renal allograft rejection [Internet]. Nephrol. Dial. Transplant. 2011; 26(12):3929-3935.

Walsh RC, Everly JJ, Brailey P, Rike AH, Arend LJ, Mogilishetty G et al. Proteasome inhibitor-based primary therapy for antibody-mediated renal allograft rejection. Transplantation **2010**;89(3):277-284.

Walsh et al, American Transplant Congress, San Diego, 1-5 May 2010; abstract # 425

Walsh RC, Brailey P, Girnita A, Alloway RR, Shields AR, Wall GE, et al. Early and late acute antibody-mediated rejection differ immunologically and in response to proteasome inhibition. Transplantation 2011;91(11):1218- 1226.

Wu Y, Han B, Luo H, Shi G, Wu J. Dipeptide boronic acid, a novel proteasome inhibitor, prevents islet-allograft rejection. Transplantation **2004**;78(3):360-366.

Zachary AA, Kopchaliiska D, Montgomery RA, Leffell MS. HLA-specific B cells: I. A method for their detection, quantification, and isolation using HLA tetramers. Transplantation **2007**;83(7):982-988.

Kuan HS, Gupta N, Venkatakrishnan K, Milton A, Girgis S et al. Population Pharmacokinetic (PK) Modelling of Bortezomib After Bolus Intravenous Injection in Cancer Patients. PAGE 2012 Abstr 2308 [www.page-meeting.org/?abstract=2308].

Nakade S, Ohno T, Kitagawa J, Hashimoto Y, Katayama M et al. Population pharmacokinetics of aprepitant and dexamethasone in the prevention of chemotherapy-induced nausea and vomiting. Cancer Chemother Pharmacol. 2008;63(1):75-83.

APPENDIX 2: Number of participating sites (investigational centers, laboratories)

Investigator list, version no. 7 of 16/04/2018

Center no.	AP-HP / Outside AP-HP	Principal investigator at the center (name, title, function)	Department	Head of department	Hospital site address	Telephone no. Fax no. Email
**001**	**AP-HP**	**Dr Rebecca SBERRO-SOUSSAN**	Transplantation Renale Adulte	Pr Christophe LEGENDRE	Hôpital Necker<br>149 rue de Sevres<br>75015 Paris	Tel : **+33 (0)1 44 49 42 51**<br>Fax : +33 (0)1 44 49 54 30<br>rebecca.sberro@nck.aphp.fr
**002**	**Outside AP-HP**	**Pr Emmanuel MORELON**<br>MD, PhD	Unite Transplantation Renale et Pancreatique	Pr Emmanuel MORELON	Hospices Civils de Lyon, Hôpital Edouard Herriot,<br>Pavillon P Place d'Arsonval<br>69003 Lyon	Tel : **+33 (0)4 72 11 01 50**<br>Fax : +33 (0)04 72 11 02 71<br>emmanuel.morelon@chu-lyon.fr
**003**	**Ouside AP-HP**	**Pr Magali GIRAL**<br>MD, PhD	Institut de transplantation, urologie et nephrologie	Pr Maryvonne HOURMANT	CHU de Nantes<br>30 Bd Jean Monnet<br>44035 Nantes Cedex 05	Tel : **+33 (0)2 40 08 74 43**<br>Fax : +33 (0)2 40 08 74 48<br>mgiral@chu-nantes.fr
**004**	**Outside AP-HP**	**Pr Nassim KAMAR**<br>MD, PhD	Departement de Nephrologie, et Transplantation d'Organes	Pr Nassim KAMAR	CHU Rangueil<br>1, avenue Jean Poulhes TSA 50032<br>31059 Toulouse Cedex 09.	Tel : **+33 (0)5 61 32 23 35**<br>Fax : 05 61 32 39 89<br>kamar.n @chu-toulouse.fr
**005**	**Outside AP-HP**	**Dr Vincent PERNIN**	Nephrologie<br>Transplantation<br>Dialyse Peritoneale	Pr Moglie LE QUINTREC	Hôpital Lapeyronie, 371, avenue du Doyen Gaston Giraud 34295 Montpellier 05	Tel: **+33 (0)4 67 33 84 76**<br>Fax : +33 (0)4 67 33 87 98<br>g-mourad@chu-montpellier.fr
**006**	**Outside AP-HP**	**Pr Pierre MERVILLE**<br>MD, PhD	Unite de Transplantation Renale et pancreatique	Pr Pierre MERVILLE	Hôpital Pellegrin,<br>Place Amelie Raba-Leon<br>33076 Bordeaux	Tel : **+33 (0)5 56 79 55 38**<br>Fax : +33 (0)5 56 79 61 07<br>pierre.merville@chu-bordeaux.fr

Center no.	AP-HP / Outside AP-HP	Principal investigator at the center (name, title, function)	Department	Head of department	Hospital site address	Telephone no. Fax no. Email
**007**	**AP-HP**	**Dr Nadia ARZOUK**<br>MD	Transplantation renale	Pr Benoit BARROU	Hôpital Pitie Salpetriere<br>47-83 Boulevard de l'Hôpital<br>75651 Paris Cedex 13	Tel : **+33 (0)1 42 17 71 14**<br>Fax : +33 (0)1 42 17 71 93<br>nadia.arzouk@psl.aphp.fr
**008**	**AP-HP**	**Pr Eric RONDEAU**<br>MD, PHD	Urgence nephrologiques et Transplantation renale	Pr Eric RONDEAU	Hôpital Tenon<br>4 rue de la Chine<br>75020 Paris	Tel : **+33 (0)1 56 01 65 10**<br>Fax : +33 (0)1 56 01 69 22<br>eric.rondeau@tnn.aphp.fr
**009**	**Outside AP-HP**	**Dr Michel DELAHOUSSE, Investigateur principal Dr Renaud SNANOUDJ** MD, PhD, Investigateur coordinateur	Nephrologie et transplantation renale	Dr Michel DELAHOUSSE	Hôpital Foch<br>40 rue Worth<br>92151 Suresnes	Tel : **+33 (0)1 46 25 20 86**<br>Fax : +33 (0)1 46 25 28 79<br>michel.delahousse@hopital-foch.org<br>**Tel : +33 (0)1 46 25 30 95**<br>Fax : +33 (0)1 46 25 28 79<br>r.snanoudj@hopital-foch.com
**010**	**AP-HP**	**Pr Philippe GRIMBERT**<br>Md, PHD	Nephrologie	Pr Philippe LANG	CHU Henri Mondor<br>Avenue de Lattre de Tassigny<br>94000 Creteil	Tel : **+33 (0)1 49 81 24 60**<br>Fax : +33 (0)1 49 81 24 52<br>philippe.grimbert@hmn.aphp.fr
**011**	**Outside AP-HP**	**Pr Bruno MOULIN**<br>MD, PhD	Nephrologie	Pr Bruno MOULIN	Nouvel Hôpital Civil-1<br>place de l'Hôpital, BP 426<br>67 091- Strasbourg	Tel : **+33 (0)3 69 55 05 07**<br>Fax : +33 (0)3 69 55 17 21<br>moulin@unistra.fr
**012**	**Outside AP-HP**	**Dr Hakim MAZOUZ**<br>MD	Nephrologie	Pr Gabriel CHOUKROUN	CHU Amiens-Picardie - Site Sud<br>80054 Amiens Cedex 1	Tel : +33 (0)3 22 45 58 65<br>Fax : +33 (0)3 22 45 56 51
**013**	**Outside AP-HP**	**Dr Celine ESTOURNET**<br>**MD**	Nephrologie	Dr Helene REBOUX	CHU Reunion site Saint-Denis<br>Allee des topazes<br>97405 Saint-Denis	Tel : 02 62 90 55 50<br>Celine.estournet@chu-reunion.fr

Center no.	AP-HP / Outside AP-HP	Principal investigator at the centre (name, title, function)	Department	Head of department	Address of hospital site	Telephone no. Fax no. Email
**014**	**Outside AP-HP**	**Pr Marc HAZZAN**	Nephrologie	Pr Christian NOEL	CHRU de Lille, Hôpital Huriez, Bd M Polonovski 59 000 Lille	Tel : +33 (0)3 20 44 67 70 marc.hazzan-2@univ-lille2.fr
**015**	**Outside AP-HP**	**Dr Dominique BERTRAND**	Nephrologie	Pr Dominique GUERROT	CHU Rouen, 147 avenue du Marechal Juin, 76230 Bois Guillaume	Tel : +33 (0)2 32 88 90 09 dominique.bertrand@chu-rouen.fr
**016**	**Outside AP-HP**	**Pr Matthias BUCHLER**	Nephrologie	Pr Jean-Michel HALIMI	CHU Bretonneau 2 boulevard Tonnelle, 37044 Tours	Tel : +33 (0)2 47 47 82 29
**501**		**Dr RENAUDIN**	Anatomie et cytologie pathologiques		Hotel Dieu, Nantes	Tel : +33 (0)2 40 08 73 90 Fax : +33 (0)2 40 08 73 99 Karine.renaudin@chu-nantes.fr
**502**		**Dr Valerie DUBOIS**	Laboratoire HLA Lyon		Etablissement Français du Sang Rhone Alpes a Lyon	Tel : +33 (0)4 72 71 17 36 Fax : +33 (0)4 72 71 17 62 Valerie.dubois@efs.sante.fr

APPENDIX 3: Banff 2013 classification and c4d staining scores (Haas 2013)

Table 2: Revised (Banff 2013) classification of antibody-mediated rejection (ABMR) in renal allografts

Acute/active ABMR; all three features must be present for diagnosis [1,2]

1. Histologic evidence of acute tissue injury, including one or more of the following:
   Microvascular inflammation (g > 03 and/or pct. > 0)
   Intimal or transmural arteritis (v > 0) [4]
   Acute thrombotic microangiopathy, in the absence of any other cause
   Acute tubular injury, in the absence of any other apparent cause
2. Evidence of current/recent antibody interaction with vascular endothelium, including at least one of the following:
   Linear C4d staining in peritubular capillaries (C4d2 or C4d3 by IF on frozen sections, or C4d > 0 by IHC on paraffin sections)
   At least moderate microvascular inflammation ([g + ptc] ≥ 2) [5]
   Increased expression of gene transcripts in the biopsy tissue indicative of endothelial injury, if thoroughly validated
3. Serologic evidence of donor-specific antibodies (HLA or other antigens)

Chronic, active ABMR; all three features must be present for diagnosis [1,6]

1. Morphologic evidence of chronic tissue injury, including one or more of the following:
   Transplant glomerulopathy (TG) (cg > 0),[7] if no evidence of chronic thrombotic microangiopathy
   Severe peritubular capillary basement membrane multilayering (requires EM) [8]
   Arterial intimal fibrosis of new onset, excluding other causes [9]
2. Evidence of current/recent antibody interaction with vascular endothelium, including at least one of the following:
   Linear C4d staining in peritubular capillaries (C4d2 or C4d3 by IF on frozen sections, or C4d > 0 by IHC on paraffin sections)
   At least moderate microvascular inflammation ([g + ptc] ≥ 2) [5]
   Increased expression of gene transcripts in the biopsy tissue indicative of endothelial injury, if thoroughly validated
3. Serologic evidence of DSAs (HLA or other antigens)

C4d staining without evidence of rejection; all three features must be present for diagnosis[10]

1. Linear C4d staining in peritubular capillaries (C4d2 or C4d3 by IF on frozen sections, or C4d > 0 by IHC on paraffin sections)
2. g = 0, ptc = 0, cg = 0 (by light microscopy and by EM if available), v = 0; no TMA, no peritubular capillary basement membrane multilayering, no acute tubular injury (in the absence of another apparent cause for this)
3. No acute cell-mediated rejection (Banff 1997 type 1A or greater) or borderline changes.

cg, Banff chronic glomerulopathy score; EM, electron microscopy; ENDAT, endothelial activation and injury transcript; g, Banff glomerulitis score; GBM, glomerular basement membrane; IF, immunofluorescence; IHC, immunohistochemistry; ptc, peritubular capillary; TCMR, T cell-mediated rejection; v, Banff arteritis score.

[1] For all ABMR diagnoses, it should be specified in the report whether the lesion is C4d-positive (C4d2 or C4d3 by IF on frozen sections; C4d > 0 by IHC on paraffin sections) or without evident C4d deposition (C4d0 or C4d1 by immunofluorescence (IF) on frozen sections; C4d0 by IHC on paraffin sections).
[2] These lesions may be clinically acute, smoldering or subclinical. Biopsies showing two of the three features, except those with donor-specific antibodies (DSA) and C4d without histologic abnormalities potentially related to ABMR or T cell-mediated rejection (TCMR) (C4d staining without evidence of rejection; see footnote 10) may be designated as “suspicious” for acute/active ABMR.
[3] Recurrent/de novo glomerulonephritis should be excluded.
[4] It should be noted that these arterial lesions may be indicative of ABMR, TCMR or mixed ABMR/TCMR. “v” lesions are scored in arteries having continuous media having two or more smooth muscle layers.
[5] In the presence of acute TCMR, borderline infiltrates or evidence of infection, ptc ≥ 2 alone is not sufficient to define moderate microvascular inflammation and g must be ≥ 1.
[6] At present the only validated molecular marker meeting this criterion is ENDAT expression (4), and this has only been validated in a single center (University of Alberta). The use of ENDAT expression at other centers or other test(s) of gene expression within the biopsy as evidence of ABMR must first undergo independent validation as was done for ENDAT expression by Sis et al (4).
[7] Lesions of chronic, active ABMR can range from primarily active lesions with early TG evident only by EM (cg 1a) to those with advanced TG and other chronic changes in addition to active microvascular inflammation. In the absence of evidence of current/recent antibody interaction with the endothelium (those features in the Second Section), the term active should be omitted; in such cases DSA may be present at the time of biopsy or at any previous time posttransplantation.
[8] Includes GBM duplication by EM only (cg1a) or GBM double contours by light microscopy.
[9] ≥7 layers in one cortical peritubular capillary and ≥5 in two additional capillaries (17), avoiding portions cut tangentially.
[10] While leukocytes within the fibrotic intima favor chronic rejection, these are seen with chronic TCMR as well as chronic ABMR, and are therefore helpful only if there is no history of TCMR. An elastic stain may be helpful as absence of elastic lamellae is more typical of chronic rejection and multiple elastic lamellae are most typical of arteriosclerosis, although these findings are not definitive.
[11] The clinical significance of these findings may be quite different in grafts exposed to anti-blood-group antibodies (ABO-incompatible allografts), where they do not appear to be injurious to the graft (18), (19) and may represent accommodation. However, with anti-HLA antibodies such lesions may progress to chronic ABMR (20) and more outcome data are needed.

Table 2: Scoring of C4d staining (% of biopsy or 5 high-power fields)

C4d0:	Negative:	0%
C4d1:	Minimal C4d stain/detection:	1 <10%
C4d2:	Focal C4d stain/positive:	10-50%
C4d3:	Diffuse C4d stain/positive:	>50%

APPENDIX 4: Bortezomib (Velcade®) SmPC

EMA SmPC

http://www.ema.europa.eu/docs/fr FR/document library/EPAR -Product Information/human/000539/WC500048471.pdf

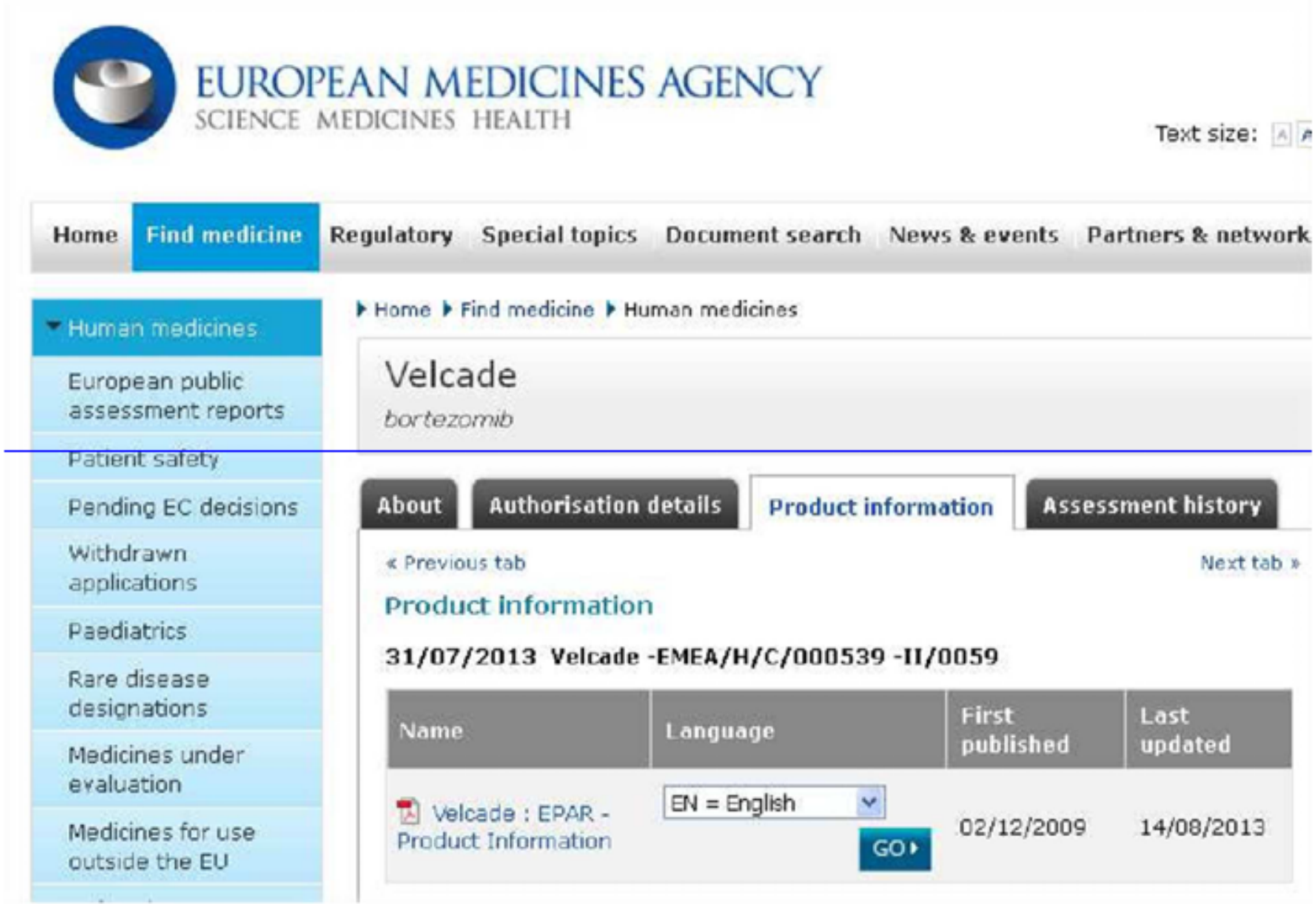

APPENDIX 5: Bortezomib-related neurotoxicity grades

Proposed in the Bortezomib (Velcade®) SmPC and adapted from version 4.0 of the "NCI Common Toxicity Criteria CTCAE" classification.

**Grade 0**	No symptoms
**Grade 1**	Paresthesia, muscle weakness and/or loss of reflexes, with no loss of function
**Grade 2**	Objective sensory and/or motor loss resulting in functional impairment and limiting instrumental activities of daily living[1]
**Grade 3**	Loss of autonomy[2]
**Grade 4**	Disabling sensory neuropathy and/or motor neuropathy that is life-threatening or leads to paralysis and/or severe autonomic neuropathy

1. Instrumental ADL: refers to preparing meals, shopping for groceries or clothes, using the telephone, managing money, etc.

2. Self care ADL: refers to bathing, dressing and undressing, feeding oneself, using the toilet, taking medicinal products, and not being bedridden.

APPENDIX 6: Other non-experimental treatments used during the study

TACROLIMUS

Refer to the SmPC for the INN used

CELLCEPT (Mycophenolate Mofetil)

EMA, SmPC

http:/ec.europa.eu/health/documents/community-register/2012/20120720123830/anx 123830 fr.pdf

MA no.: 359529.3 (rev 28/02/08)

MA holder: Roche

Pharmaceutical form: 250 mg capsules: Blue and brown oblong capsules, printed in black with "CellCept 250" on the cap and the company logo on the body. Box of 100 capsules, packaged in blister packs of 10 capsules.

500 mg film-coated tablets: Lavender, capsule-shaped tablets, marked "CellCept 500" on one side and the company logo on the other. Box of 50 capsules, packaged in blister packs of 10 capsules.

Powder for oral suspension, 1 g/5 mL: Bottle containing powder with a child-resistant cap + 2 oral dosing syringes graduated in mL and 1 bottle adapter. After reconstitution, the suspension volume is 175 mL, corresponding to a deliverable volume of 160–165 mL.

Route of administration: Oral

Duration of Treatment: Immunosuppressive therapy must be maintained to prevent graft rejection. Therefore, no maximum duration of oral treatment can be specified.

Composition:

Capsule:	*(per capsule)*
Mycophenolate mofetil	*250 mg*

Excipients: Pregelatinized maize starch, croscarmellose sodium, povidone (K-90), magnesium stearate. Capsule shell: Gelatin, indigo carmine (E132), yellow, red and black iron oxides (E172), titanium dioxide (E171), potassium hydroxide, and shellac.

Tablet:	*per capsule*
Mycophenolate mofetil	*500 mg*

Excipients: Tablet core: Microcrystalline cellulose, povidone (K-90), croscarmellose sodium, and magnesium stearate. Film coating: Hypromellose, hydroxypropyl cellulose (hyprolose), titanium dioxide (E171), polyethylene glycol 400, indigo carmine aluminium lake (E132), and red iron oxide (E172).

Powder for oral suspension	*per 5 ml**
Mycophenolate mofetil	*1 g*

*Excipients: Sorbitol, colloidal anhydrous silica, sodium citrate, soy lecithin, mixed fruit flavoring, xanthan gum, aspartame** (E951), methyl parahydroxybenzoate (E218), and anhydrous citric acid. Each bottle contains 35 g of mycophenolate mofetil in 110 g of powder for oral suspension.*

** of reconstituted suspension*

*** Aspartame contains phenylalanine equivalent to 2.78 mg per 5 mL of suspension.*

CORTANCYL (prednisone)

ANSM, SmPC 1 mg

. **http://base-donnees-publique.medicaments.gouv.fr/affichageDoc.php?specid=60118715&typedoc=R**

ANSM, SmPC 5 mg

. **http://base-donnees-publique.medicaments.gouv.fr/affichageDoc.php?specid=60492702&typedoc=R**

ANSM, SmPC 20 mg

. **http://base-donnees-publique.medicaments.gouv.fr/affichageDoc.php?specid=62390849&typedoc=R**

MA no. 332838.5 (rev 26/07/07)

MA holder: SANOFI-AVENTIS

Pharmaceutical form: 1 mg tablet (white): Box with 30 tablets in blister packs.

5 mg scored tablet (white): Box of 30 tablets in blister packs.

20 mg scored tablet (white): Box with 20 tablets in blister packs.

Route of administration: Oral

Duration of Treatment: 1 mg/kg/day on post-transplant days 1–4, followed by 0.5 mg/kg/day on days 3–4, then taper by 5 mg every 7 days. Minimum maintenance dose is 10 mg/day. Discontinue at 3 months.

Composition:

Prednisone (INN)	*1 mg*
or	*5 mg*
or	*20 mg*

Common excipients: Lactose, maize starch, talc, and magnesium stearate.

MYFORTIC (sodic mycophenolic acid)

ANSM, SmPC 180 mg

. **http://base-donnees-publique.medicaments.gouv.fr/affichageDoc.php?specid=69678277&typedoc=R**

ANSM, SmPC 360 mg

. **http://base-donnees-publique.medicaments.gouv.fr/affichageDoc.php?specid=67858369&typedoc=R**

MA no.: 363091.9 (rev 05/03/08)

Ma holder: Novartis Pharma SAS

Pharmaceutical form: 180 mg gastro-resistant film-coated tablets: Round, beveled edges, greenish-yellow tablets debossed with the imprint "C" on one side. Box of 120 tablets, in blister packs of 10 tablets.

360 mg gastro-resistant film-coated tablets: Oval, pale orange-red tablets with the imprint "C" on one side. Box of 120 tablets, in blister packs of 10 tablets.

Route of administration: Oral

Duration of treatment: Immunosuppressive therapy should be maintained to prevent graft rejection. Therefore, no maximum duration of oral treatment can be specified.

Mycophenolic acid (INN)	180 mg
or	360 mg

(as mycophenolate sodium: 192.4 mg/tablet at 180 mg; 384.8 mg/tablet at 360 mg)

Composition:

Common excipients: Maize starch, povidone K30, crospovidone, anhydrous lactose, colloidal anhydrous silica, magnesium stearate. *Film coating*: 180 mg tablet: Hypromellose phthalate, titanium dioxide (E171), yellow iron oxide (E172), and indigo carmine (E132). 360 mg tablet: Hypromellose phthalate, titanium dioxide (E171), yellow iron oxide (E172), and red iron oxide (E172).

Lactose content: 180 mg tablet: 45 mg lactose per tablet; 360 mg tablet: 90 mg lactose per tablet.

|

DEXAMETHASONE

MYLAN 20mg/5ml inj sol. MA holder: Mylan - RH
ANSM, SmPC
http://base-donnees-publique.medicaments.gouv.fr/affichageDoc.php?specid=65822036&typedoc=R

PHARMACEUTICAL FORM

Injectable solution

COMPOSITION

Dexamethasone sodium phosphate
Strength: Equivalent to 20 mg dexamethasone phosphate per 5 mL ampoule.

Excipients: Creatinine, sodium metabisulfite, sodium citrate, methyl parahydroxybenzoate, propyl parahydroxybenzoate, sodium hydroxide, water for injections.

CTRS Laboratory, NEODEX 40 mg scored tablets [French cohort Temporary Authorisation for Use (ATUc)] – Centralized Marketing Authorisation: 16/03/2016 (NEOFORDEX)

http://base-donnees-publique.medicaments.gouv.fr/extrait.php?specid=68572869

PHARMACEUTICAL FORM

40 mg scorable tablet

COMPOSITION

Each tablet contains dexamethasone acetate equivalent to 40 mg of dexamethasone.

Excipients: Lactose monohydrate, microcrystalline cellulose, magnesium stearate, colloidal anhydrous silica.

APPENDIX 7: SAE Reporting Form

Version 4.0 of 12/12/2018

APPENDIX 8: Follow-up form for a pregnancy occurring during biomedical research

(Version 2.0 of 12/12/2018)

FOLLOW-UP FORM FOR A PREGNANCY OCCURRING DURING BIOMEDICAL RESEARCH

ASSISTANCE PUBLIQUE HOPITAUX DE PARIS

FOR SPONSOR USE ONLY: DO NOT COMPLETE

|_|_| - |_|_| - *DRCD* - |_|_|_|_| - |_|_|_|_|

<u>As soon as the investigator becomes aware of the pregnancy</u>, this form must be duly completed (3 pages), signed, and returned <u>without delay</u> to the DRCD Headquarters Vigilance Unit by <u>fax</u> to: au +33 (0)1 44 84 17 99

Date of end-of-pregnancy follow-up: |__|__| (dd) |__|__| (mm) |__|__|__|__| (yyyy)

Research Code: P120119/TRIBUTE **N° EudraCT : 2013-002531-15**

Title of the Biomedical Research: TRIBUTE : Bortezomib for the treatment of chronic active antibody-mediated rejection

Center name and address : ______________________________

Centre no. : |_|_|_| Investigator (Name – Surname – Function) : ______________________________

1: PARTICIPANT IDENTIFICATION:

Participant ref. : |_|_|_| (center no.) |_|_|_|_| (Enrollment no.) |_| (Surname (initial)) |_| (First name (initial))

Date of birth: |_|_| |_|_| |_|_|_|_| Enrollment date: |_|_| |_|_| |_|_|_|_|

Date of last menstrual period: : |_|_| |_|_| |_|_|_|_| Pregnancy start date: |_|_| |_|_| |_|_|_|_|

Exposures: Smoking ☐ Alcohol use ☐ Drug use☐ Other ☐

If yes, specify the amounts: ______________________________

2: HISTORY:

Medical ______________________________

Surgical ______________________________

Obstetric: SAB ☐ IAB ☐ MTOP ☐ EP ☐ IUFD ☐
|_|_| gravida |_|_| para

3: COURSE OF THE PREGNANCY:

3.1- <u>Investigational</u> medicinal product(s) administered during pregnancy

Brand name (preferably) or International Nonproprietary Name (INN)	Route of administration	Dose /24h	Start date	Ongoing	End date	Indication
	|_|_|		|_|_| |_|_| |_|_|_|_|	☐	|_|_| |_|_| |_|_|_|_|	
	|_|_|		|_|_| |_|_| |_|_|_|_|	☐	|_|_| |_|_| |_|_|_|_|	
	|_|_|		|_|_| |_|_| |_|_|_|_|	☐	|_|_| |_|_| |_|_|_|_|	
	|_|_|		|_|_| |_|_| |_|_|_|_|	☐	|_|_| |_|_| |_|_|_|_|	

3.2- Concomitant medication(s) administered during pregnancy:

Brand name (preferably) or International Nonproprietary Name (INN)	Route of administration	Dose /24h	Start date	Ongoing	End date	Indication
	|_|_|		|_|_| |_|_| |_|_|_|_|	☐	|_|_| |_|_| |_|_|_|_|	
	|_|_|		|_|_| |_|_| |_|_|_|_|	☐	|_|_| |_|_| |_|_|_|_|	
	|_|_|		|_|_| |_|_| |_|_|_|_|	☐	|_|_| |_|_| |_|_|_|_|	
	|_|_|		|_|_| |_|_| |_|_|_|_|	☐	|_|_| |_|_| |_|_|_|_|	

3.3- Description of the medical device (MD):

Type	Model/type/reference	Serial no./Lot no.	Supplier name	Manufacturer name	**If the MD is sterile**: Sterilization date and Expiry date
					|_|_| |_|_| |_|_|_|_| and |_|_| |_|_| |_|_|_|_|

Date of implantation or MD use: |__|__| |__|__| |__|__|__|__|

3.4- If accessory, consumable:

Name	Model	Serial no./Lot no.	Manufacturer name	Expiry date
				|_|_| |_|_| |_|_|_|_|

Date of implantation or accessory use: |__|__| |__|__| |__|__|__|__|

3.5- Other:

____

____

____

____

____

3.6- Additional information:

Hospitalization during pregnancy: no ☐ yes ☐

If yes, why?

____

HTN ☐ Diabetes ☐ Infection ☐ TPL ☐ Other ☐

Polyhydramnios ☐ Oligohydramnios ☐

Intrauterine growth restriction: no ☐ yes ☐

Ultrasound(s): |_|_| |_|_| |_|_|_|_|: Results: ______________________________

|_|_| |_|_| |_|_|_|_|: Results: ______________________________

Karyotype : no ☐ yes ☐

Specific laboratory tests (abnormal

Participant ref. : |_|_|_| center no. |_|_|_|_| Enrollment no. |_| Surname (initial) |_| First name (initial)

Research code: P120119/TRIBUTE

BIRTH:

Date : |_|_| |_|_| |_|_|_|_|: Term : |_|_| Weeks of gestation Premature: no ☐ yes ☐ Multiple pregnancy: no ☐ yes ☐

Delivery: Normal ☐ Induced ☐ Cesarean section ☐

Amniotic fluid: clear ☐ stained ☐

Placenta: normal ☐ abnormal ☐

Acute fetal distress: no ☐ yes ☐

Chronic fetal distress: no ☐ yes ☐

Medications during labor: General anesthesia ☐ Epidural ☐ Spinal anesthesia ☐

Comments:

5: NEWBORN :

Weight : |_|_|_| kg

Height: |_|_|_| cm

Sex: male ☐ female ☐

APGAR score: 1 minute 5 minutes: 10 minutes:

HC: |_|_|_| cm Normal ☐ Stillbirth ☐ Dysmature ☐ Small for gestational age ☐

Malformations: ____

Non-malformative neonatal pathology: ____

Comments: ____

6: COMMENTS:

APPENDIX 9: Ancillary tests

Methodology for study selection

To improve understanding of the mechanisms underlying chronic antibody-mediated rejection, a national call for proposals for ancillary studies of the TRIBUTE study was launched by the CENTAURE Foundation at the beginning of 2013. The proposals were scored, and ranked by the independent International Scientific Committee of the CENTAURE Foundation (www.fondation-centaure.org). The four highest-scoring ancillary studies were selected and awarded equivalent funding.

Description of ancillary studies

The main objective of these ancillary studies is to improve understanding of the pathogenesis of anti-HLA antibodies. The recent introduction of Luminex Single Antigen technology has led to a redefinition of the concept of anti-HLA sensitization. Unlike conventional lymphocytotoxicity assays, the majority of patients awaiting transplantation are now found to carry anti-HLA antibodies (approximately 80%). The presence of donor-specific antibodies, detected by Luminex Single Antigen testing before or after transplantation, is a risk factor for antibody-mediated rejection and graft dysfunction. However, antibody detection alone and antibody quantification remain poorly predictive of rejection occurrence. It is therefore important to characterize the features of anti-HLA antibodies that confer pathogenic potential beyond their purely quantitative aspects. The aim of these tests is, among other objectives, to identify new markers of pathogenicity that may help to better identify patients and clinical situations at highest risk.

The four ancillary studies are summarized in Table 1, at the end of the document.

1) **In situ Detection of Donor-Specific Anti-HLA Antibodies at Diagnosis and Following Treatment of Chronic Antibody-Mediated Rejection in Patients from the TRIBUTE Study**

Prinicpal Investigator: Prof. JL Taupin, Bordeaux University Hospital (CHU de Bordeaux), France

Rationale:

Anti-HLA antibodies are currently detected in the peripheral blood; however, their ability to bind to the graft and exert a potential pathogenic effect remains unknown. In a preliminary study involving 51 kidney transplant recipient biopsies performed for graft dysfunction, we recently demonstrated that in situ detection of donor-specific anti-HLA antibodies (DSAs) was a stronger predictor of antibody-mediated rejection-associated renal lesions and poor graft function outcomes than simple detection of DSA in serum (Bachelet et al.; Am J Transplant, 2013). These findings suggest that in situ detection of DSAs could be an important tool for improving the diagnosis of rejection and predicting its clinical course.

Objectives:

These findings need to be confirmed in an independent cohort, and the impact of rejection treatment on this parameter should be investigated in order to validate its potential use in routine clinical practice. The cohort of 100 patients included in the TRIBUTE study is particularly suitable for addressing these two objectives, as it consists of patients with antibody-mediated rejection and includes two biopsies for follow-up: the first at diagnosis and the second at one year to assess treatment efficacy.

Methodology:

At diagnosis and at 1 year, a kidney biopsy sample and a serum sample (250 µL) collected on the same day will be stored frozen at −80°C for all 100 patients included in the study. The biopsy samples will additionally be unfixed. DSAs will be assessed using the One Lambda Single Antigen assay, for both HLA Class I and Class II, on kidney biopsy eluates and serum samples. Samples will be shipped, frozen on dry ice, to the Immunology Laboratory of Bordeaux University Hospital (*Laboratoire d'Immunologie du CHU*) every 3 to 6 months depending on recruitment, for batch analysis. It is essential that serum collected on the same day as the biopsy is available in order to interpret the results. This study does not require any additional sampling, as graft biopsies are already planned within the protocol at D0 and M12, and only a biopsy fragment will be dedicated to the detection of in situ anti-HLA antibodies.

2) **Study of the Sialylation of Donor-Specific Anti-HLA Antibodies**

Prinicpal Investigator: Dr Sophie Brouard, Dr Nicolas Degauque, Nantes, France

Rationale

The presence of donor-specific anti-HLA antibodies (DSAs) is associated with poorer long-term graft function and an increased prevalence of chronic rejection. However, despite the presence of these antibodies, some kidney transplant recipients maintain stable graft function for many years after transplantation. An important current question is whether the nature of these antibodies and their biochemical structure could explain a "non-aggressive" or even protective biological activity towards the kidney graft. The glycosylation of antibodies influences their biological activity and could contribute to variability in the pathogenic potential of anti-HLA antibodies, particularly by affecting their susceptibility to proteolytic degradation. Our team has demonstrated that stable kidney transplant recipients had higher levels of sialylated IgG at the time of transplantation (Day 0) compared with patients who subsequently developed rejection.

Objective

We have developed a method for analyzing the sialylation of total antibodies (ELISA) and anti-HLA antibodies (Luminex Single Antigen Beads assay) in order to study the kinetics of this specific glycosylation pattern. This technique has been validated in a small patient cohort (n = 45). The objective of this project within the framework of the national PHRC (Hospital Clinical Research Programme) TRIBUTE study is to compare the level of sialylation of donor-specific and non-donor-specific anti-HLA antibodies in a cohort of 100 patients who all develop de novo anti-HLA antibodies after transplantation. These patients will receive treatment with IVIg, plasma exchanges, and dexamethasone, with (treatment arm) or without (control arm) Velcade® for the treatment of histologically proven chronic active antibody-mediated rejection. This study may determine whether the use of a treatment targeting the proteasome, and therefore the plasma cells responsible for anti-HLA antibody production, can modify the glycosylation of anti-HLA antibodies, particularly those directed against the donor (DSAs). It will also assess whether the presence of sialylated antibodies in this cohort of patients treated with or without Velcade® is associated with improved prognosis, assessed by stabilization of histological lesions and kidney function over the medium term.

Methodology

This study is possible due to the recruitment of patients into the TRIBUTE trial. Briefly, an ELISA based on the use of a biotinylated lectin will be used to detect total sialylated IgG and to determine the ratio of sialylated to total IgG. Subsequently, the sialylated antibodies will be purified and analyzed using the Luminex Single Antigen assay to determine whether these antibodies are anti-HLA antibodies and, more

specifically, donor-specific antibodies. This analysis will be performed in parallel with flow cytometric phenotyping of B and T lymphocytes from the same patients.

All 100 patients enrolled in the study will be analyzed. The following samples will be collected at baseline (Day 0) and

at the end of the study (Month 12):

- 2 mL of serum (in a 6 mL serum tube) for analysis of the sialylation of de novo anti-HLA antibodies.
- One 2.5 mL PAXgene® tube for analysis of the T-cell repertoire.
- 10 million cells, collected in an ACD tube, isolated by Ficoll density-gradient centrifugation, frozen in DMSO, together with a complete blood count (one 8.5 mL ACD tube; Nantes and Paris patients only; n = 40 [20 per treatment arm]).

3) **Study of the Molecular and Cellular Basis of the Pathogenicity of Anti-HLA Antibodies**

Principal Investigator: Prof. L. Chatenoud (Hôpital Necker), Prof. D. Charron (Hôpital Saint-Louis)

Rationale

The factors determining the pathogenicity of anti-HLA antibodies remain poorly understood. At present, only donor specificity and antibody titer are routinely characterized. Other structural characteristics of the antibodies, including features of the variable region (specificity, clonality, and affinity) and the constant region (isotype and complement-fixing capacity), may also contribute to their pathogenic potential. Furthermore, the pathogenic mechanisms of anti-HLA antibodies associated with endothelial injury and transplant glomerulopathy remain poorly understood. The aim of this study is therefore to investigate the molecular characteristics of de novo donor-specific anti-HLA antibodies (DSAs) that develop after transplantation and are associated with pathogenicity, as well as their impact on the phenotype and function of endothelial cells and monocytes.

Objectives

The pathogenicity of anti-HLA antibodies will be investigated at three levels:

- Biochemical characterization of the constant region (isotype) and the variable region (antigenic and epitope specificity, affinity).
- Identification of donor-specific alloreactive memory B cells and characterization of their epitope specificity (clonality), together with assessment of the effect of bortezomib on this lymphocyte population.
- Investigation of the mechanisms underlying anti-HLA antibody-mediated endothelial injury, and the effects of anti-HLA antibodies on the regulation of endothelial cell–monocyte interactions.

Ten patients from each group will be evaluated at Day 0 and Month 3 after enrolment in the study.

Methodology

- Biochemical analysis of DSAs will be performed following a preliminary purification step using recombinant HLA molecules. Their molecular heterogeneity will be assessed by analyzing their epitope specificity (analysis of isolated specificities identified using the Luminex Single Antigen assay with the HLAMatchmaker software), their isotype, and by isoelectric focusing and 2D electrophoresis methods. The affinity of DSAs will be evaluated using surface plasmon resonance.
- Detection of alloreactive memory B lymphocytes will be performed using an in vitro polyclonal stimulation approach, previously tested in the Immunology Laboratory of Necker Hospital, followed by analysis of the specificity of the antibodies produced using Matchmaker software.
- The impact of DSAs on endothelial cells will be evaluated in in vitro culture models by studying intracellular signaling pathways in endothelial cells and their ability to activate and expand CD4+ lymphocyte subpopulations. Endothelial cell–monocyte interactions will also be investigated in these in vitro models.

The structural and functional parameters identified will be correlated with the clinical and biological

parameters of therapeutic response to bortezomib in the patients studied.

Methodology for structural analysis:

Twenty patients with DSAs will be studied at enrolment in the protocol (10 mL blood sample collected in a serum tube).

Methodology for cellular analysis:

The same 20 patients will be studied: a 34 mL blood sample (four 8.5 mL ACD tubes) will be sent to the Immunology Laboratory of Necker Hospital for analysis of alloreactive B lymphocytes (plasma will also be collected after separation of mononuclear cells). These must be transported within 24 hours after collection and at room temperature. Cellular analysis will be repeated at the 3-month visit (four ACD tubes – 34 mL).

Methodology for functional analysis:

The following samples will be used:

- 10 serum samples (volume: 100 µL) from patients with de novo anti-HLA-DR12 or DR18 DSAs;
- 5 serum samples (volume: 100 µL) containing class II anti-HLA antibodies other than DR12 or DR18;

and

- 5 serum samples (volume: 100 µL) containing only class I HLA antibodies.

Ancillary tests: Summary table

**Ancillary test**<br>**Principal Investigator**	**Number of patients**	**Sample type**	**First time**	**Second time point**
**1) In situ Detection of Anti-HLA Antibodies**<br>**JL Taupin**	100	- biopsy fragment	D0	M12
**2) Sialylation of Anti-HLA Antibodies**<br>**S Brouard / N Degauque**	n=100<br><br>n=40 (20 per arm)	- 6 mL serum tube<br>- 2.5 mL PAXgene® tube<br><br>- 8.5 mL ACD tube (Nantes and Paris centers)	D0	M12
**3) Molecular and Cellular Basis of the Pathogenicity of Anti-HLA Antibodies**<br>**L Chatenoud / D Charron**	10 per arm = 20	- 10 mL serum tube<br>- 4 ACD tubes = 34 mL	D0	M3 (ACD tubes)

Blood samples for time point 1 (Day 0) of the ancillary studies may be collected on the day of the first plasma exchange, before the start of the exchange, in order to avoid taking too many samples on the day of the enrolment visit.
