# Supplementary material for: Bortezomib in combination with standard-of-care for late active antibody-mediated rejection with de novo donor-specific antibodies after kidney transplantation: a multicenter randomized trial
Source: Transpl Int. 2026 Sep 23;39:16702. doi: 10.3389/ti.2026.16702 (PMC13642227; doi:10.3389/ti.2026.16702)
Supplement: Supplementary file 2 [file Table1.docx]

**Supplementary Materials**

**Supplementary Table 1****.** Primary endpoint among patients with MFI of idDSA at baseline > 15,000

| Endpoint (each one assessed at 12 months) | Patients | | RR  [95% CI] ^†^ | p-value |
| --- | --- | --- | --- | --- |
|  | **Bortezomib group** | **Standard-of-care group** |  |  |
| Primary endpoint (ITT) | 8/19 (42.1) | 1/12 (8.3) | 5.05 [0.72; 35.48] | 0.10 |
| Secondary endpoints |  |  |  |  |
| *>50% reduction in idDSA MFI (ITT analysis)* | 10/19 (52.6) | 2/12 (16.7) |  | 0.065 |
| *Stabilization or improvement of histological AMR lesions*  *(ITT analysis)* | 11/19 (57.9) | 6/12 (50.0) |  | 0.67 |

ITT; intention to treat, PP; per protocol, DSA; donor specific antibodies, idDSA; immunodominant DSA, dnDSA; de novo DSA, MFI; mean fluorescence intensity, RR; risk ratio, CI; confidence interval. All values according to central reading expressed as n/ntot (%). ^†^Considering standard-of-care group as reference

**Supplementary Table 2.** Association between treatment arm and primary criterion, adjusted for baseline imbalances

| Association between treatment arm and primary endpoint |  | |
| --- | --- | --- |
|  | **RR [95%CI] ^†^** | **p-value** |
|  |  |  |
| Without adjustment | **1.20 [0.61; 2.34]** | **0.59** |
| Adjusted for sex | 0.96 [0.46; 2.00] | 0.37 |
| Adjusted for class of idDSA at baseline | 1.06 [0.54; 2.08] | 0.87 |
| Adjusted for rank of transplantation | 1.27 [0.65; 2.50] | 0.49 |
| Adjusted for MFI of the idDSA | 1.12 [0.59; 2.11] | 0.74 |

idDSA; immunodominant DSA, MFI; mean fluorescence intensity, RR; risk ratio. ^†^RR and 95% CI obtained by log-binomial regression models, considering standard-of-care group as reference
